# Structure of the NAT10 acetyltransferase and mechanism of tRNA acetylation

**DOI:** 10.1038/s41467-026-74222-6

**Published:** 2026-06-20

**Authors:** Mingyang Zhou, Supuni Thalalla Gamage, Khoa A. Tran, David Bartee, Xuepeng Wei, Boyu Yin, Shelley Berger, Jordan L. Meier, Ronen Marmorstein

**Affiliations:** 1 Department of Chemistry, University of Pennsylvania, Philadelphia, PA USA; 2 Abramson Family Cancer Research Institute, Perelman School of Medicine, University of Pennsylvania, Philadelphia, PA USA; 3 Chemical Biology Laboratory, National Cancer Institute, Frederick, MD USA; 4 Department of Cell and Developmental Biology, Perelman School of Medicine, University of Pennsylvania, Philadelphia, PA USA; 5 Epigenetics Institute, Perelman School of Medicine, University of Philadelphia, Philadelphia, PA USA; 6 Department of Biochemistry and Biophysics Perelman School of Medicine, University of Pennsylvania, Philadelphia, PA USA; 7 Department of Biology, School of Arts and Sciences, University of Pennsylvania, Philadelphia, PA USA; 8 Department of Genetics, Perelman School of Medicine, University of Pennsylvania, Philadelphia, PA USA; 9 Present Address: Guangzhou National Laboratory, Guangzhou, China

**Keywords:** Cryoelectron microscopy, Transferases

## Abstract

NAT10 is the sole eukaryotic acetyltransferase that catalyzes N4-acetylcytidine (ac^4^C) modification of RNA. While dysregulation of NAT10 is associated with cancer and premature aging syndromes, the requirement for its acetyltransferase activity and how NAT10 coordinates catalysis and RNA binding remain poorly understood. Here, we report single particle cryo-electron microscopy structures of eukaryotic (*Chaetomium thermophilum*) NAT10 in complex with a designer cytidine-CoA cofactor-based ligand in the presence and absence of ADP. NAT10 forms a symmetrical heart-shaped dimer where a Gcn5-related N-acetyltransferase (GNAT) domain with an atypically opened active site is flanked by conserved helicase and RNA-binding domains. Biochemical reconstitution of NAT10 in the presence of the adapter protein THUMPD1 reveals that tRNA acetylation is enabled by two conserved active site residues (His548 and Tyr549 in *Ct*NAT10) and two basic patches: one proximal and one distal from the active site, and suggests that binding orientation rather than affinity drives catalysis. Finally, we harness structure-guided mutations in cellular studies to demonstrate the necessity for NAT10 catalytic acetyltransferase activity in fungal thermoadaptation and mammalian etoposide-induced cellular senescence, respectively. Our findings provide a structural foundation for understanding NAT10-catalyzed cytidine acetylation, with implications for regulation and therapeutic targeting of its distinct RNA acetyltransferase activity.

## Abstract (web summary)

RNA acetylation by NAT10 is linked to disease, but its catalytic mechanism is unclear. Here, the authors report cryo-EM structures of *Chaetomium thermophilum* NAT10 in complex with a cytidine-CoA probe with and without ADP.

## Introduction

Nature uses chemical modifications to create functional diversity from a limited repertoire of biological building blocks. One prototypical example of this is acetylation. In mammalian cells, the vast majority of proteins are acetylated on the epsilon amine of lysine^1,2^ or at their N-terminus^3–5^. Nucleic acid acetylation is also found in all domains of life, occurring in humans as N4-acetylcytidine (ac^4^C) in RNA^6^.

Eukaryotic RNA acetylation is catalyzed by a single enzyme, *N*-acetyltransferase 10 (NAT10)^7–11^. NAT10 is essential in all known eukaryotes, ranging from vertebrates to yeast^7,10,12,13^. The vast majority of eukaryotic ac^4^C is found in 18S ribosomal RNA (rRNA) and transfer RNA (tRNA)^10,14^. 18S rRNA modification occurs at C1280/C1337 on helix 34, and C1773/C1842 on helix 45 in *S. cerevisiae* and *H. sapiens*, respectively, and is facilitated by specific box C/D snoRNAs (snR4 for C1280 and snR45 for C1773 in yeast, with SNORD13 guiding modification at C1842 in humans)^15–18^. Acetylation of tRNA occurs at C12 in eukaryotic leucine and serine tRNA and is assisted by the adapter protein THUMPD1 (Tan1 in yeast)^7^. Biophysical analyses have shown that N4-acetylation of cytidine thermodynamically stabilizes duplex RNA, including within tRNA D-arm surrogates^19^. Consistent with this, disruption of eukaryotic tRNA acetylation has been found to decrease Leu/Ser tRNA stability, increase ribosome pausing, and activate stress response pathways associated with ribosome collisions^14,20^. In addition to its catalytic acetyltransferase activity, NAT10 appears to contribute to the proper assembly and maturation of the 90S pre-ribosome via non-catalytic roles, including making interactions with other ribosome biogenesis factors^21–23^. Although rRNA acetylations are associated with ribosome biogenesis, they are not strictly required for this process^7,9,11,12^. Previous mutational analysis in yeast indicates an essential role for NAT10’s helicase domain, potentially due to its role in ribosome assembly. In contrast, the catalytic RNA acetyltransferase domain is required for optimal fitness at elevated temperatures, which is attributed to its role in tRNA modification^7,24–27^. Context-dependent roles in enhancing translation efficiency through mRNA acetylation have also been proposed^8,28^.

The biological relevance of NAT10 is further supported by its associations with various diseases. Genetic disruption of NAT10-catalyzed RNA acetylation can correct nuclear architecture defects and phenotypes associated with premature aging in models of Hutchinson-Gilford Progeria Syndrome (HGPS)^29^, and reduces metastatic spread in preclinical models of breast cancer^24,30^. While these findings raise the possibility of targeting NAT10 for therapy, defining the druggable role of NAT10-catalyzed acetylation remains challenging. Eukaryotic NAT10 enzymes are large ( ~ 120 kDa) multidomain proteins. In addition to the Gcn5-related N-acetyltransferase (GNAT) domain directly responsible for ac^4^C formation, NAT10 also possesses an N-terminal ATP-dependent helicase domain, a C-terminal RNA-binding domain, and several other regions of notable sequence homology. These regions interact with ribosome biogenesis factors and may contribute to the non-catalytic role of NAT10 in the proper assembly and maturation of the 90S pre-ribosome^21–23^. Furthermore, mutation of NAT10’s helicase and acetyltransferase domains causes distinct growth defects in *S. cerevisiae*^7,24–27^. Notably, many studies of NAT10 in human cells have employed genetic techniques that target the entire gene, leaving the specific contribution of NAT10’s acetyltransferase activity to phenotypes unknown. A putative small molecule inhibitor of NAT10 has been reported, but is unsuitable for use as a chemical probe due to its pleiotropic off-target effects^31^. In addition, the substrate spectrum of NAT10 is unclear. While NAT10’s ability to catalyze the deposition of ac^4^C in ribosomal RNA (rRNA) and transfer RNA (tRNA) is well-validated, other protein and RNA substrates remain topics of inquiry^32,33^. Outside of the association of tRNA acetylation with neurodevelopmental defects, what specific RNA substrates mediate individual disease phenotypes is largely unknown^34^. Altogether, these data highlight a need for data capable of relating structure and biochemical activity on NAT10 acetylation to specific cellular phenotypes.

Initial structural studies of RNA cytidine acetyltransferases have focused on related bacterial enzymes, which primarily modify the wobble base of the initiator tRNA_Met_. Crystal structures of the bacterial (*Escherichia coli*) homolog of NAT10 (TmcA) in complex with acetyl-CoA and ADP^35^ revealed a monomeric L-shaped morphology, with the helicase and structured DUF699 mimicking DEAD-box RNA helicase abutting the GNAT, followed by a tail region proposed to contribute to tRNA binding^12^. Structural analyses of eukaryotic NAT10 have been limited to cryo-electron microscopy (cryo-EM) structures of the 90S pre-ribosome from *Chaetomium thermophilum* (*Ct*) and *Saccharomyces cerevisiae* (*Sc*). Although this structure only resolved the NAT10 portion to ~ 4.5 Å, it revealed that NAT10 (fungal Kre33) forms a head-to-head and tail-to-tail homodimer on the ribosome and cooperates with Bms1, Enp2, Brf2 and Lcp5 proteins to mediate the final compaction of the 18S rRNA subdomain onto the 90S pre-ribosome^36,37^. This complex is likely distinct from those involved in NAT10-catalyzed rRNA and tRNA acetylation, since (as described above) the former requires specific box C/D snoRNAs and may belong to a larger C/D box snoRNP complex, while the latter is assisted by THUMPD1 and expected to occur outside the nucleolus^15–18^.

Given that the molecular features of eukaryotic NAT10 responsible for its cytidine-specific RNA acetylation activity remain unresolved, we set out to provide new molecular insights into this area. Here, we report the structure of *Chaetomium thermophilum* NAT10 (*Ct*NAT10) bound to a cytidine-CoA probe with and without ADP. Our data reveal that stand-alone NAT10 forms a symmetrical heart-shaped dimer with conserved functional domains from both subunits surrounding two acetyltransferase active sites. Structure-based mutagenesis and tRNA acetylation activity measurements indicate a critical role for THUMPD1, two conserved active site residues, and two basic patches proximal and distal to the active site for tRNA-specific acetylation. Applying these insights in functional studies, we corroborate the importance of NAT10 catalytic activity in yeast fitness under temperature stress and demonstrate this property can also impact cellular senescence in mammalian cells. Finally, we compare NAT10 to histone and N-terminal acetyltransferase structures, which reveals an open active site that appears more tailored for RNA-specific acetylation. Overall, our studies provide molecular-level insights into eukaryotic RNA acetyltransferase activity, providing a foundation for domain-selective inhibition and functional studies of NAT10 in human disease.

## Results

### Expression, reconstitution, and cytidine-CoA binding of CtNAT10

**Fig. 1:**
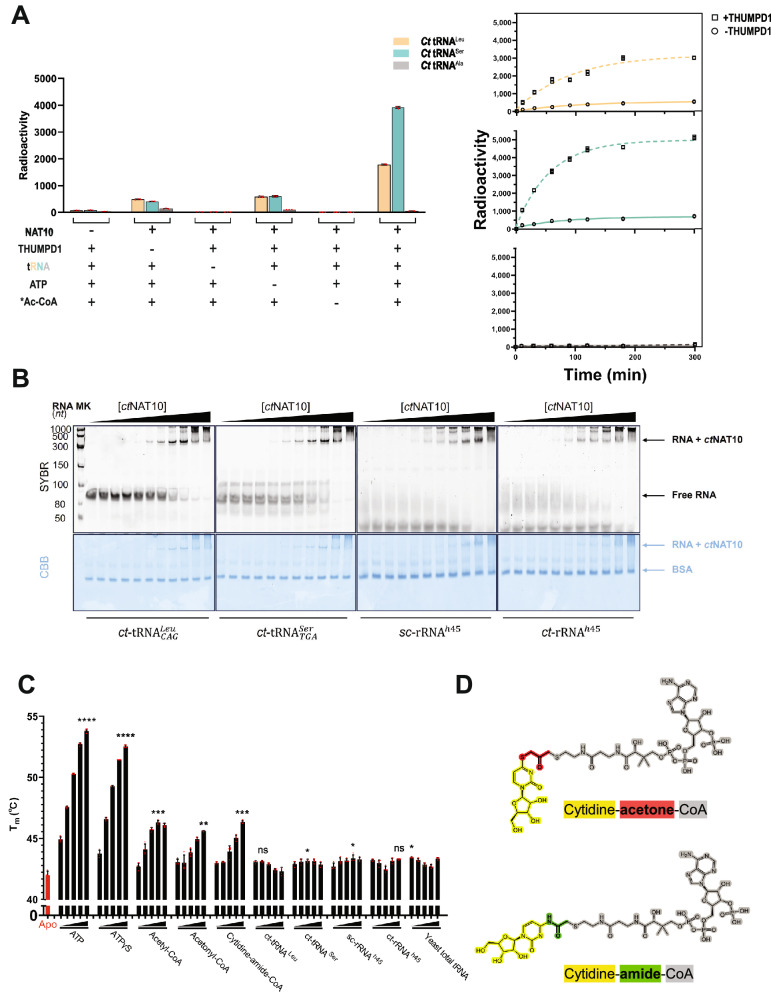
Biochemical characterization of recombinant NAT10 and cytidine-CoA probe compounds. **A** Radioactive acetylation assays to monitor incorporation of radiolabeled acetyl groups into the indicated tRNA substrates. Left panel – Endpoint assay with all or lacking components (“+” indicates added and “−” indicates not added). Reactions were incubated at 45 °C for 90 min. Right panels - Time course reactions carried out in the presence (+) or absence (-) of *Ct*THUMPD1 in the presence of cognate (tRNA^Ser^ and tRNA^Leu^) or non-cognate (tRNA^Ala^) RNA. Radioactivity reflects the incorporation of the radiolabeled acetyl group over time. Data points represent the mean ± SD of 3 technical replicates, with individual data points overlaid as red dots. Source data are provided as a **Source Data** file. **B** Electrophoretic mobility shift assays of *Ct*NAT10 binding to tRNAs and rRNAs (6% polyacrylamide native gels) stained with SYBR™ Gold (upper panel) and Coomassie Brilliant Blue (lower panel) to visualize RNA and protein, respectively. The experiment was performed in a single replicate. RNA input was fixed at 5 pmol, and *Ct*NAT10 was titrated at 0, 0.2, 0.4, 0.7, 1.5, 3, 6, 11, 23, 45, 90 pmol (from left to right), with 0.2 mg/ml BSA added as a loading control and to prevent non-specific binding. RNA-*Ct*NAT10 complex, free RNA, and BSA bands are indicated with arrows. **C** Differential Scanning Fluorimetry of *Ct*NAT10 (1 μM) in the absence (red) and presence of substrates, metabolic ligands, non-hydrolyzable analogs, and cytidine-CoA probe (black). Ligands were evaluated from low to high concentration as follows: ATP, ATPɣS, Acetyl-CoA and acetonyl-CoA: 0.25, 2.5, 25, 250, 2500 μM; Cytidine-amide-CoA: 0.20, 2.0, 20, 200, 2000 μM; ct-tRNA^Leu^, ct-tRNA^Ser^, sc-rRNA^h45^ and ct-rRNA^h45^: 0.625, 1.25, 2.5, 5, 10 μM; Yeast total RNA: 2.5, 5, 10, 20, 40 μM. Averaged values of three technical replicates with S.D. are shown. *P*-values are indicated for the melting temperature of each ligand at the concentration that maximizes the thermal stability relative to the apo protein.* P*-values were calculated by Brown-Forsythe and Welch ANOVA with Dunnett’s T3 multiple comparisons test (one-sided), with individual variances computed for each comparison (ns: not significant, ∗: *P* < 0.05, ∗∗: *P* < 0.01, ∗∗∗: *P* < 0.001, ∗∗∗∗: *P* < 0.0001, the exact *P*-value from left to right: < 0.0001, < 0.0001, 0.0004, 0.0030, 0.0003, 0.0863, 0.0491, 0.0239, 0.0561, 0.0416). Individual data points are overlaid as red dots. Source data are provided as a **Source Data** file. **D** The sub-structure portions of cytidine-CoA bisubstrate probes are highlighted (cytidine in yellow, bridging linkers in red and green, and CoA in gray).

Human and fungal NAT10 enzymes share highly similar domain architecture and sequence conservation (Supplementary Fig. 1), leading us to pursue *Chaetomium thermophilum* NAT10 (*Ct*NAT10) as an initial target for structural and biochemical characterization. *C. thermophilum* is widely used for structural studies of challenging eukaryotic proteins due to the enhanced thermostability of its protein products^38,39^. Accordingly, recombinant full-length *Chaetomium thermophilum* NAT10 (*Ct*NAT10) was expressed from both *Escherichia coli* (*E.coli*) and *Spodoptera frugiperda* (*Sf9*) (Supplemental Fig. 2A, B). The bacterial- and *Sf9*-produced proteins were used interchangeably for structural studies, while the *Sf9*-produced protein was used exclusively for functional studies, as it was slightly more active. To evaluate the catalytic activity of recombinant *Ct*NAT10 towards tRNA, we performed a radioactive activity assay using cognate *Ct*tRNA^Ser^ and *Ct*tRNA^Leu^ substrates. When stand-alone *Ct*NAT10 was incubated with tRNA, acetyl-CoA, and ATP and assayed for transfer of radiolabeled-acetyl group to tRNA, very little activity was observed (Fig. 1A). Given that THUMPD1 is required for tRNA acetylation in all eukaryotes characterized to date, we hypothesized that its addition may accelerate the biochemical reaction, leading us to express and purify recombinant full-length *Ct*THUMPD1 (Supplemental Fig. 3). Indeed, the presence of *Ct*THUMPD1 markedly enhanced *Ct*NAT10 activity while maintaining the same time-dependent increase with saturation (Fig. 1A, left). In contrast, when a non-cognate *Ct*tRNA^Ala^ substrate was tested as a NAT10 substrate, no detectable activity was observed regardless of the presence or absence of THUMPD1 (Fig. 1A, right). This result underscores the rate acceleration conferred by THUMPD1 as well as the substrate specificity of the NAT10–THUMPD1 complex towards tRNA.

Evaluating the RNA-binding activity of *Ct*NAT10 via a gel shift assay in the presence of three cognate substrates (tRNA^Leu^, tRNA^Ser^, rRNA^h45^) and one non-cognate substrate (tRNA^Ala^) RNAs revealed evidence of concentration-dependent complex formation in all cases (Fig. 1B and Supplementary Fig. 4A). This is consistent with the ability of *Ct*NAT10 to non-specifically bind RNA. Addition of *Ct*THUMPD1 did not alter cognate versus non-cognate NAT10-tRNA complex formation, and additional binding order experiments confirmed that cognate and non-cognate tRNAs bind with similar affinity regardless of whether THUMPD1 is pre-incubated with NAT10 or tRNA (Supplemental Fig. 4A). These results are consistent with a model whereby THUMPD1 accelerates tRNA acetylation through a post-binding mechanism, for example by optimally positioning tRNA substrates for catalysis, rather than by enhancing cognate versus non-cognate substrate affinity. The mechanistic implications of these findings are described further in the Discussion in the context of our structural analyses.

As a precursor to structural studies of NAT10, we probed NAT10’s thermal stability as a function of binding to its substrates and small molecule ligands. To do this, we employed differential scanning fluorimetry (DSF) (Fig. 1C). While apo *Ct*NAT10 displayed a 42.0 ± 0.3 °C melting temperature (T_m_), statistically significant increases in thermal stabilization were observed in the presence of Mg^2+^ - ATP (53.8 ± 0.1 °C), acetyl-CoA (46.1 ± 0.1 °C), and their non-hydrolyzable analogs. Interestingly, binding of tRNA and rRNA substrates as well as THUMPD1 (Supplemental Fig. 4B) did not significantly increase *Ct*NAT10 thermostability, suggesting these interactions may occur at surface sites rather than within stabilizing active site cavities occupied by the small molecule cofactors.

Bisubstrate probes, designed to simultaneously occupy cofactor and substrate sites, have proven valuable tools for studying protein acetyltransferase enzyme function^40–42^. To test whether this strategy could be potentially extended to RNA acetyltransferases, we designed and synthesized two compounds as possible mimics of the transition state, cytidine-acetone-CoA and cytidine-amide-CoA (Fig. 1D), whose structures differ only in the presence of an acetone or amide linker, respectively. The cytidine-amide-CoA probe (46.4 ± 0.1 °C) demonstrated thermal stabilization comparable to acetyl-CoA, suggesting a similar mode of interaction and a minimal contribution of the cytidine to binding energy (Fig. 1C). These studies set the stage for structural studies by establishing the biochemical activity and ligand-binding properties of a reconstituted eukaryotic RNA acetyltransferase enzyme.

### Architecture of a liganded eukaryotic NAT10 acetyltransferase

**Fig. 2:**
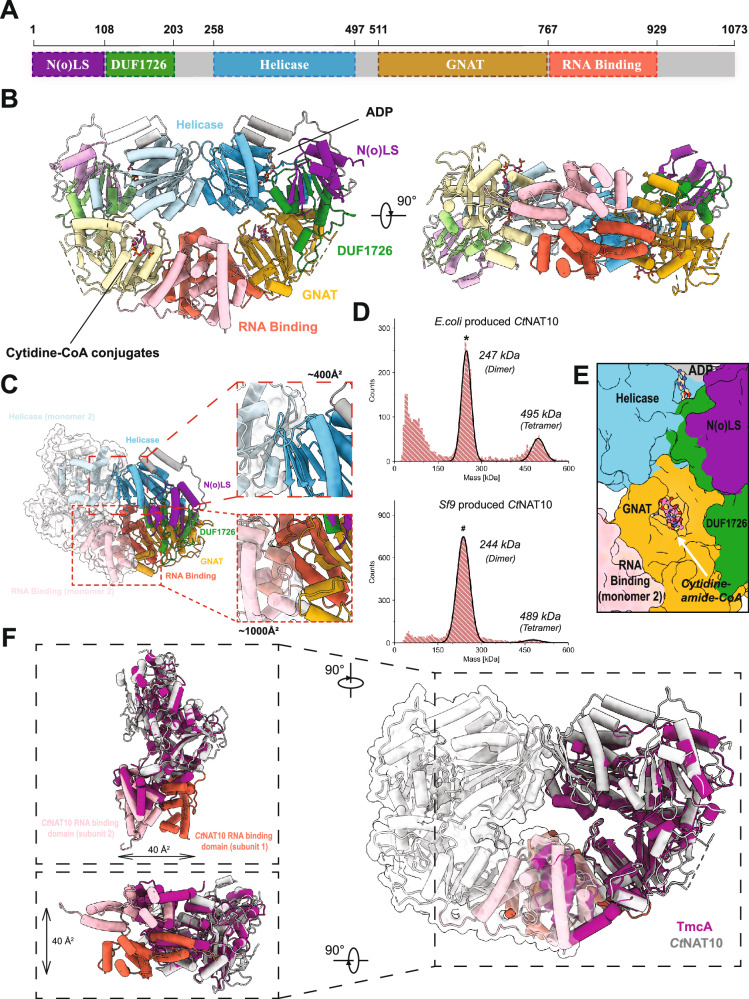
Overall structure of *Ct*NAT10 and comparison to TmcA. **A** Scaled schematic representation of *Ct*NAT10 domain organization, with nomenclature and residue count. **B** Color-coded cartoon of overall *Ct*NAT10 dimer structure (subunits shown in light and dark shades of color) using the *Sf9* produced recombinant *Ct*NAT10/cytidine-amide-CoA/ADP structure as the representative. Each domain is colored and named corresponding to the domain schematic representation in (**A**). **C** Structural features associated with *Ct*NAT10 dimerization. One subunit of the *Ct*NAT10 dimer is colored like B, while the domains of the other subunit are depicted as a ghostly white cartoon and surface representation, with the exception of the RNA-binding (light salmon) and helicase (light sky blue) domains that directly participate in dimerization. **D** Characterization of the oligomeric state of recombinant *Ct*NAT10 by mass photometry. Histograms in panels show the particle counts of *E.coli* (up) and *Sf9* (bottom) produced recombinant *Ct*NAT10 at the indicated molecular mass. Black lines are Gaussian fits to the peaks, and the mass of the Gaussian fit are indicated above each peak. In both samples, *Ct*NAT10 was mostly present as a dimer (theoretical MW of *Ct*NAT10 dimer: 241.02 kDa), and the dimer peaks for *E.coli* (*) and *Sf9* (^#^) samples are indicated. **E** Close-up view of the GNAT active site. Each domain is colored and named corresponding to the domain schematic representation in A. ADP and cytidine-amide-CoA are depicted in ball-and-stick representation. **F** Superimposition of *Ct*NAT10 and *E. coli* TmcA. TmcA is colored in purple. *Ct*NAT10 subunit 1 is shown in gray except for the tRNA-binding domain (orange); subunit 2 is depicted in ghostly white with transparent surface except for the tRNA-binding domain (light salmon). The aligned structures are rotated by 90° to the left and upward to more clearly display side (left-up panel) and bottom (left-bottom panel) views, respectively, highlighting the relative spatial positions of the *Ct*NAT10 and *E. coli* TmcA tRNA-binding domains. All *Ct*NAT10 schematic diagrams in Fig. 2 are derived from the Sf9 produced *Ct*NAT10/cytidine-amide-CoA/ADP structure (PDB: 9B0I).

With the active recombinant *Ct*NAT10 in hand, we next set out to prepare the liganded protein for single-particle cryo-EM structure determination. Three structures were produced: *E.coli*-derived recombinant *Ct*NAT10/cytidine-acetone-CoA, *E.coli*-derived recombinant *Ct*NAT10/cytidine-amide-CoA/ADP, and *Sf9*-derived recombinant *Ct*NAT10/cytidine-amide-CoA/ADP at overall resolutions of 3.3 Å, 3.2 Å, and 3.0 Å, respectively (Table 1 and Supplemental Figs. 5, 6, and 7). The *Ct*NAT10/cytidine-acetone-CoA structure was initially modeled using *E. coli* TmcA (*Ec*TmcA) as a template. As the micrographs suggested two-fold symmetry, C_2_ symmetry was applied during refinement (Supplemental Fig. 5). The *Ct*NAT10/cytidine-acetone-CoA model was then used to build both the *E.coli* and *Sf9-*produced *Ct*NAT10/cytidine-amide-CoA/ADP models. The three structures superimpose well, showing no significant conformational changes due to the presence of ADP or cytidine-CoA probes, with an RMSD of less than 1.30 Å between all Cα atoms (Supplemental Fig. 8). All the previously reported functional domains are well-resolved in the three structures analyzed, and the regions corresponding to these functional domains exhibit minor variations from the residue ranges documented in prior reports (Fig. 2A). In addition, a novel N(o)LS domain was defined, which contains a putative nuclear localization signal (NLS) and nucleolar localization signal (NoLS) motifs that are unique to the eukaryotic NAT10 proteins^32^. Each subunit of the two-fold symmetric *Ct*NAT10 dimer contains 31 helices and 22 strands forming three sheets within the N(o)LS/DUF1726, helicase, and GNAT domains, while the RNA binding domain is exclusively helical (Fig. 2B and Supplementary Fig. 9).

**Table 1:** Cryo-EM data collection, refinement, and validation statistics

	*Ct*NAT10_EC_ /cytidine-acetone-CoA EMD-43996 PDB: 9AYM	*Ct*NAT10_EC_ /cytidine-amide-CoA/ADP EMD-44038 PDB: 9B0E	*Ct*NAT10_Sf9_ /cytidine-amide-CoA/ADP EMD-44042 PDB: 9B0I
**Data collection and processing**			
Magnification	105,000	105,000	105,000
Voltage (keV)	300	300	300
Electron exposure (e/Å^2^)	45	45	42.9
Defocus range (μm)	− 1.0 to − 2.5	− 1.0 to − 3.0	− 1.0 to − 3.0
Pixel size (Å)	0.415	0.415	0.43
Number of images	5500	7351	5986
Symmetry imposed	C2	C2	C2
Initial particles (no.)	1,228,342	407,808	6,212,400
Final particles (no.)	160,385	73,181	162,769
Map resolution (Å)	3.29	3.19	3.02
FSC threshold	0.143	0.143	0.143
**Refinement**			
Initial model used (PDB code)	2ZPA	2ZPA	2ZPA
Model resolution (Å)	3.4	3.4	3.2
FSC threshold	0.5	0.5	0.5
Map sharpening *B* factor (Å^2^)	− 151.2	− 109.9	− 128.6
**Model composition**			
Non-hydrogen atoms	12,950	13,158	13,040
Protein residues	1624	1642	1628
Ligands	2	4	4
***B*** **factors (Å**^**2**^**)**			
Protein	71.02	112.55	45.32
Ligand	69.68	107.17	37.18
**R.M.S. deviations**			
Bonds lengths (Å)	0.005	0.002	0.003
Bond angles (°)	0.678	0.642	0.624
**Validation**			
MolProbity score	1.58	1.41	1.32
Clash score	10.09	7.55	4.21
Poor rotamers (%)	0.28	0.28	0.14
**Ramachandran plot**			
Favored (%)	97.76	98.21	97.39
Allowed (%)	2.24	1.79	2.61
Disallowed (%)	0	0	0

Unlike *Ec*TmcA, which is an L-shaped monomer, the *Ct*NAT10 is a head-to-head and tail-to-tail heart-shaped dimer that is stabilized by head domain interactions between helicase domains and tail domain interactions between the RNA binding domains that bury solvent-excluded surfaces of ~ 400 Å^2^ and ~ 1000 Å^2^, respectively (Fig. 2C). This dimeric configuration is also observed in the 90S pre-ribosome-bound form of *Ct*NAT10 (also known as Kre33) (RMSD of 1.185 Å, Supplementary Fig. 8)^36^. Notably, the residues involved in dimerization are ~90% conserved through eukaryotic NAT10 proteins, but not in *Ec*TmcA (Supplementary Fig. 1). Mass photometry of the *Ct*NAT10 at a concentration of 10 nM also shows the presence of a *Ct*NAT10 dimer at physiological concentrations, together supporting the biological relevance of a *Ct*NAT10 dimer (Fig. 2D), even in the absence of ribosome binding^36^. Importantly, the *Ct*NAT10 dimer places each of the functional domains around the symmetric GNAT active sites, with the RNA-binding domain coming from the symmetry-related subunit (Fig. 2E).

Although *Ct*NAT10 and *Ec*TmcA share only 24.8% sequence identity (calculated by Clustal Omega^43^), the functional domains align well (RMSD of 2.471 Å over 301 common Cα atoms), with the exception of the RNA-binding domain (Fig. 2F). Compared to *Ec*TmcA, in the *Ct*NAT10 structure, the RNA-binding domain shifts ~ 40 Å towards the symmetry-related *Ct*NAT10 subunit. Interestingly, the RNA-binding domain of the symmetry-related subunit superimposes much more closely to the position of the RNA-binding domain of *Ec*TmcA (Fig. 2F), thus further supporting the biological importance of the dimeric state of NAT10.

### ADP binds at the helicase-DUF domain interface without altering the overall CtNAT10 structure

**Fig. 3:**
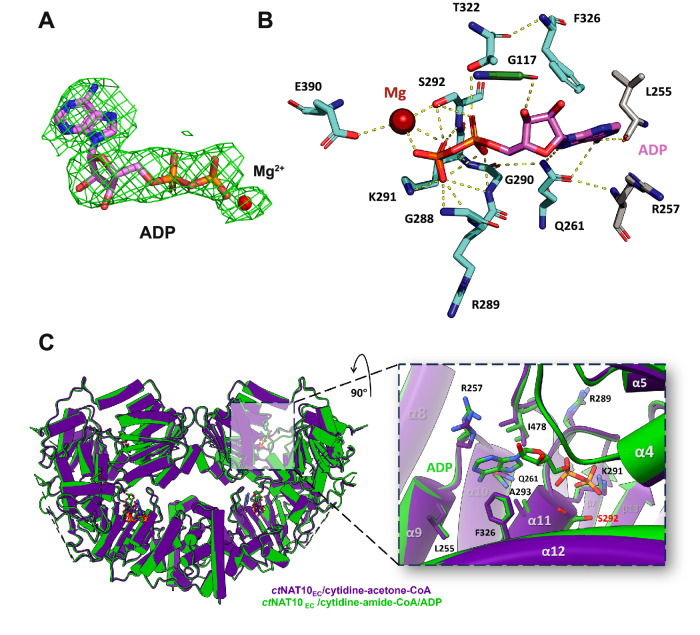
ATP binding site of *Ct*NAT10. **A** Cryo-EM density of ADP and Mg^2+^ ion. **A** contour level of 8 σ was applied (figure derived from PDB: 9B0I). **B** Close-up view of ADP/Mg^2+^ - protein interactions (figure derived from PDB: 9B0I). Bound ADP (magenta) and key residues involved in interactions with ADP/Mg^2+^ are drawn as sticks and are labeled. The Mg^2+^ ion (maroon) is shown as a spherical ball. Hydrogen bonds are indicated by yellow-colored dashed lines, with pi-pi interaction and hydrophobic interaction not explicitly shown. Atoms are colored as follows: C -the same as the domain color where the residue is located, N -blue, S -orange and O -red. **C** Left - Structure comparison of *E.coli* produced *Ct*NAT10 bound to bisubstrate cytidine-CoA probe analog with and without ADP. *Ct*NAT10/cytidine-acetone-CoA is shown in purple, and *Ct*NAT10/cytidine-amide-CoA/ADP is shown in green. Right - Selected residues involved in interactions with ADP are drawn as sticks and are labeled in the zoom-in view and shown with the same color-coding as in the left. Ser292, the only residue that changes orientation to accommodate the binding of ADP, is highlighted with a red label. The α-helices and β-sheets involved in ADP coordination are labeled and numbered as in Supplementary Fig. 8.

While ATP was added to the cryo-EM samples with *Ct*NAT10 (*Sf9* produced) and the cytidine-amide-CoA, in our modeling attempts, ATP could not be refined to satisfactory stereochemistry within the density, whereas ADP, together with a coordinated Mg^2+^ ion, fit the map well and refined without stereochemical outliers. Based on this observation, we hypothesized that *Ct*NAT10 catalyzed ATP hydrolysis in the absence of a cognate tRNA substrate, resulting in ADP with bound Mg^2+^ (Fig. 3A). The observation that added ATP could only be modeled as ADP was also made in the structure of *Ec*TmcA^35^, where the residues that are involved in ADP coordination are largely conserved (Fig. 3B). In the *Ct*NAT10/cytidine-amide-CoA/ADP structure, the ADP and Mg^2+^ are wedged in between the helicase and DUF domains, and most of the protein interactions are mediated by the α-helix bundle (α9-α12) from the helicase domain (Fig. 3C and Supplementary Fig. 9). The adenosine group makes hydrophobic interactions with either the side chain or main chain carbon atoms of residues Gly117, Arg257, Gln261, pi-pi interaction with Phe326, and hydrogen bond interactions with residues Leu255, and Gln261. The pyrophosphate moiety of ADP is mainly held by the side chain of Lys291, several backbone amide nitrogen atoms spanning residues Gly288-Ser292, and two hydrogen bonding interactions from Thr322 with the α-phosphate and the side chain of Ser292 with the β-phosphate. Mg^2+^ is chelated by the β-phosphate of ADP and the side chain of Ser292 and Glu390. A comparison of the *Ct*NAT10/cytidine-CoA probe structures with and without ADP reveals that ADP binding does not make significant changes to the overall structure of *Ct*NAT10, with an RMSD of 0.506 Å over 1,479 common Cα atoms (Fig. 3C and Supplementary Fig. 8). The only residue that changes its orientation upon ADP binding is Ser292, which rotates by ~ 180° (Fig. 3C). Together, these findings demonstrate that ATP binding and hydrolysis do not significantly change the NAT10 structure, at least in the absence of a cognate cytidine-containing RNA substrate.

### The active site tunnel is surrounded by conserved domains and implicates important residues for catalysis

**Fig. 4:**
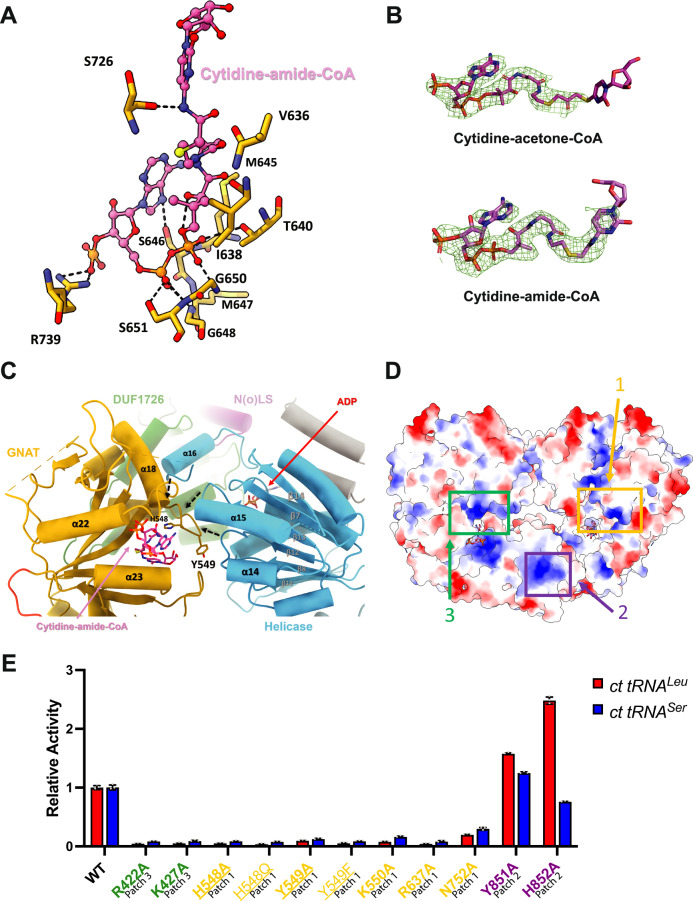
Acetyl-CoA, Cytidine and RNA binding sites of *Ct*NAT10. **A** Close-up view of interactions between Cytidine-amide-CoA and *Ct*NAT10 protein (figure derived from PDB: 9B0I). Cytidine-amide-CoA (pink) is drawn with ball-and-stick representation and labeled with the same color, and residues that make a hydrogen bond and with Cytidine-amide-CoA are shown by black-colored dashed lines. Van der Waals interaction and hydrophobic interaction are not explicitly shown but described in the main text. Atoms are color-coded as in Fig. 3. **B** Cryo-EM density of Cytidine-acetone-CoA (figure derived from PDB: 9AYM) and Cytidine-amide-CoA (figure derived from PDB: 9B0I). Contour levels of 7 σ and 8 σ was shown, respectively. **C** Putative catalytic residues His548 and Tyr549 (green) within the GNAT domain (gold) are shown proximal to the helicase domain (blue), where movement of the helicase domain can influence positioning of these residues. Bound ADP (red) and Cytidine-amide-CoA (lavender) are drawn as sticks and are labeled with the corresponding color. Dotted black arrows are drawn to suggest how the helicase domain may modulate the positioning of His548 and Tyr549. Figure derived from PDB: 9B0I. **D** Electrostatic surface diagram of the *Ct*NAT10 structure (derived from PDB: 9B0I), highlighting 3 color-coded regions (yellow, purple, and green) selected for mutagenesis and functional studies. Neutral, electropositive and electronegative surfaces of the *Ct*NAT10 structure are colored white, blue and red, respectively. **E** Relative enzyme activity of *Ct*NAT10 mutant constructs to wild type (WT). Residues are color-coded according to the regions in Fig. 4D. Putative catalytic residues (His548 and Tyr549) are underlined. Data presented as mean ± standard deviation, *n* =  3 technical replicates. Individual data points are overlaid as gray dots. Source data are provided as a **Source Data** file.

As expected, both cytidine-acetone-CoA and cytidine-amide-CoA probes are bound to the GNAT domain of *Ct*NAT10, which is located roughly in the middle of each of the protein subunits. Interestingly, our structural and biochemical analyses revealed no significant differences in binding mode or protein stabilization between the amide and acetone linkers, suggesting the nature of the connecting region has minimal impact on probe recognition. Given the structural similarity between the two probe-bound complexes, all subsequent analyses were performed using the *Ct*NAT10/cytidine-amide-CoA/ADP structure. In the *Ct*NAT10/cytidine-amide-CoA/ADP structure, the adenosine and pyrophosphate group of the cytidine-CoA probe is mainly clamped into place by van der Waals interactions and hydrogen bonds between Met647, the backbone amides of residues Ser646, Gly648, Gly650 and the side chains of residues Thr640, Ser646, Ser651, with the pyrophosphate group; and the side chains of residues Ser646 and Arg739 with the adenine base and 3′-phosphate group of the adenosine. The pantetheine arm rests inside a largely hydrophobic tunnel and is stabilized by several hydrophobic interactions from Ile638 and Met645 (Fig. 4A). Consistent with most GNAT superfamily members^44^, the thiol of the CoA portion protrudes between a V-shape gap, between 2 parallel beta-strands (β18 and β20) of *Ct*NAT10 (Supplementary Fig. 9).

Although the cryo-EM density for the cytidine-CoA probes clearly shows the CoA portion, the linkers and cytidine nucleotide are more poorly resolved (Fig. 4B), suggesting that the structures that we captured represent a pre-reaction state rather than a transition state mimic. Detailed inspection of this area reveals that the amide linker makes van der Waals interactions with the backbone of Val636 and hydrogen bond interactions with the sidechain -OH of Ser726 and the amide -NH, but no protein interactions are made with the cytidine portion (Fig. 4A). This observation explains the relatively poor density of the cytosine base, with no density observed at all for the terminal sugar ring, and suggests that the nucleotide is more flexibly positioned. This greater flexibility of the cytidine nucleotide could suggest that other regions of the RNA substrate are required to appropriately position the cytosine base for acetylation.

Inspection of the active site proximal to the cytidine nucleotide reveals a somewhat open pocket with no residues in proximity to make direct contact, suggesting that a structural rearrangement may be required for N4-cytidine acetylation. Consistent with this possibility, the only two residues within ~13 Å of the cytidine N4 that could play a role in chemistry are His548 and Tyr549, which sit on a loop between helices α18 and α19 of the GNAT domain. His, Tyr, and Glu residues are typically used as general base residues for catalysis by GNAT proteins^3,45^. Interestingly, the loop harboring the putative catalytic residues sits against several loops within the helicase domain, where a structural change in the helicase domain (due to ATP hydrolysis, RNA substrates binding, or both) could propagate to the loop harboring His548 and Tyr549 (Fig. 4C). Consistent with the potential importance of these residues to catalysis, they are evolutionarily conserved within eukaryotic NAT10 as well as within bacterial (TmcA) and archaeal (TK0754) homologs (Supplemental Fig. 2)^10,35^. Prior work has also found *E.coli tmc*AΔ strains supplied with a TmcA mutant in which the residues corresponding to His548 and Tyr549 of eukaryotic NAT10 are mutated to alanine are poorly able to rescue ac^4^C formation^35^. To directly assess the catalytic roles of His548 and Tyr549, we generated both alanine and conservative mutations (H548Q and Y549F) at each residue. While all mutants exhibited thermal stability comparable to the wild-type protein, none retained detectable catalytic activity. The complete loss of activity upon both alanine and conservative mutations strongly suggests that His548 and Tyr549 are essential for catalysis rather than for structural integrity. (Fig. 4D, E and Supplementary Fig. 10).

### Electropositive patches on the NAT10 surface participate in RNA acetylation

To gain further insights into the potential RNA-binding mode of NAT10, we attempted to generate AlphaFold3 models of *Ct*NAT10 bound to tRNA or rRNA substrates in the presence and absence of *Ct*THUMPD1. However, these studies did not yield high-confidence predictions, as inferred by chain-pair Predicted Aligned Error (PAE) values that were generally above 5 Å, except for the *Ct*NAT10 dimer itself (data not shown). Consequently, we considered these computational models unsuitable for guiding further experiments. To experimentally probe the functionality of potential RNA-binding surfaces on *Ct*NAT10, we targeted three electropositive patches for mutagenesis and assessed these mutants for acetyltransferase activity and RNA binding using cognate tRNA^Ser^ and tRNA^Leu^ substrates (Fig. 4D, E, Supplementary Figs. 4A, B and 10). Surface 1 was located on the front side of the GNAT domain and most proximal to the active site, surface 2 was located on the RNA binding domain of the opposing subunit and located about 20 Å away from the active site and surface 3 was located on the back side of the GNAT domain surface of the opposing subunit and located about 60 Å away from the active site (Fig. 4D). Both sites 2 and 3 were within distances that could conceivably participate in the binding of extended regions of tRNA and rRNA substrate molecules.

Mutations within all three patches had no major effects on protein integrity or thermal stability as assessed by size exclusion chromatography, mass photometry, and DSF analyses (Supplemental Fig. 4). However, their impact on catalytic activity varied substantially. In the presence of the *Ct*THUMPD1 cofactor, mutations in patches 1 and 3 significantly reduced acetyltransferase activity. In contrast, the patch 2 mutant retained catalytic activity comparable to, or even exceeding, that of the wild-type enzyme (Fig. 4E). To further investigate the relationship between these basic patches and tRNA recognition, we compared the ability of wild-type NAT10 and two representative basic-patch mutants (K427A and R637A) to form complexes with cognate (tRNA^Ser^ and tRNA^Leu^) and non-cognate (tRNA^Ala^) substrates by EMSA (Supplemental Fig. 4A). Remarkably, no detectable differences in tRNA-binding behavior were observed between them and wild-type protein.

To demonstrate that the EMSA assays shown in Supplementary Fig. 4A resulted in the formation of a ternary NAT10/THUMPD1/tRNA complex when all three components were present, we performed mass photometry of the complex with and without THUMPD1 or NAT10 at the concentrations comparable to those shown at the highest concentrations titrated in Supplementary Fig. 4A. Since Mass photometry experiments can only be carried out at low nanomolar concentrations, prior to diluting for mass photometry, we crosslinked the samples with glutaraldehyde to prevent dissociation of the complexes formed at the higher concentrations. These experiments demonstrate that a complex of calculated molecular weight of NAT10/THUMPD1/tRNA of 2:2:2 is only formed when NAT10, tRNA and THUMPD1 are present, but not with either NAT10 or THUMPD1 absent (Supplementary Fig. 4B). Notably, this complex is absent in the uncrosslinked sample, suggesting it is not stable at low nanomolar concentrations.

Notably, mutations within these basic patches did not affect the binding of substrate acetyl-CoA (Supplementary Fig. 10D). The observation that mutation of these basic patches reduces catalytic activity without affecting bulk RNA-binding, protein stability, or acetyl-CoA occupancy parallels our findings with THUMPD1 (Supplementary Fig. 4A), again suggesting that NAT10-catalyzed cytidine acetylation involves regulatory mechanisms that operate downstream of initial substrate binding. Whether this occurs through substrate positioning, conformational changes, or other catalytic mechanisms requires further investigation.

### Putative catalytic and RNA binding mutants exhibit temperature sensitivity in yeast

**Fig. 5:**
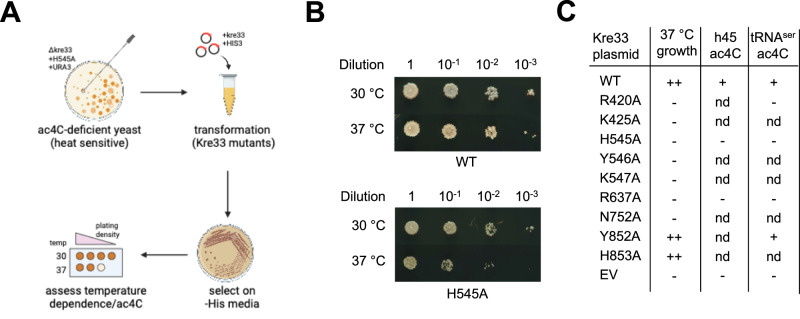
In vivo assessment of conserved NAT10 residues by functional complementation. **A** Scheme for yeast functional complementation assay. Assays were performed in a parent yeast strain (Δkre33 + pSH35) in which the endogenous kre33 allele has been deleted replaced with a *Sc*Kre33 H545A (*Sc*H545A, corresponding to *Ct*H548A) variant. The lack of Kre33 catalytic activity causes this strain to exhibit a growth defect at high temperatures^7^. Yeast were transformed individually with the kre33 plasmids expressing the indicated mutants under TDH3 control with a His3 selection marker. Transformants were grown on -His media and transformation confirmed by PCR amplification and sequencing. Serial 10-fold dilutions were plated on YPD media and incubated for 2 d at the indicated temperatures to assess rescue of temperature-sensitive growth. Created in BioRender. Thalalla Gamage, S. (https:BioRender.com/489v597). **B** Validation of assay demonstrating that temperature-sensitive growth is rescued by WT *Sc*Kre33 (top) but not *Sc*H545A mutant (bottom). Strains were grown, diluted, plated on YPD plates, and grown at the indicated temperatures as described in A. The same experimental strategy was subsequently used for all other mutant variants described in Fig. 5C. **C** Summary of rescue of temperature-sensitive growth defects and RNA acetylation by Kre33 mutants. ++, complete rescue of growth defect; +, partial rescue of growth defect or RNA acetylation; -, no rescue of growth defect or RNA acetylation. nd = not determined. Growth and RNA acetylation assay data is provided in the Supplementary Information.

To understand whether these biochemical observations extended to living organisms, we developed a functional complementation assay to assess the in vivo effects of NAT10 mutations in yeast. This assay is based on a previously developed strain of *Saccharomyces cerevisiae*, where endogenous NAT10 (*Sc*Kre33) has been deleted and rescued with a constitutively expressed inactive mutant (Δkre33 + *Sc*H545 A), which grows normally under optimal conditions (30 °C) but exhibits decreased fitness at elevated temperatures (37 °C; Fig. 5A)^7^. Our hypothesis was that ectopic expression of catalytically active *Sc*Kre33 would rescue this growth defect, while inactive variants would not. Since *Ct*NAT10 and *Sc*Kre33 are highly homologous, we used our biochemical studies to guide the design of expression vectors encoding wild-type (WT) *Sc*Kre33 as well as several putative acetyltransferase activity-disrupting variants. As expected, we observed that WT-*Sc*Kre33 increases the growth of this strain at 37 °C, while an empty vector does not (Fig. 5B). Transfections with mutant vectors corresponding to putative catalytic mutants of *Ct*NAT10 (*Sc*H545A/*Ct*H548A and *Sc*Y546A/*Ct*Y549A) as well as mutants in basic patches 1 and 3 failed to rescue the temperature sensitivity, while basic patch 2 mutants (*Sc*Y852A/*Ct*Y851A and *Sc*H853A/*Ct*H852A) did show some rescue (Fig. 5C and Supplementary Fig. 11).

To confirm that rescue of growth correlated with rescue of RNA acetylation, we applied nucleotide resolution ac^4^C sequencing to the total RNA extracted from yeast strains expressing WT *Sc*Kre33 or key mutants^46^. Assessing 18S rRNA helix 45 (*Sc*-h45), we observed efficient rescue of ac^4^C1773 by the WT plasmid, while neither the catalytic mutant ScH545A nor the patch 1 mutant ScR637A had any effect. To directly test whether these phenotypes also correlated with tRNA acetylation - the substrate for which in vitro activity was characterized – we extended this analysis to tRNA^Ser^_AGA_ across WT, ScH545A, ScR637A, ScR420A, and ScY852A strains. Only WT and the patch 2 mutant ScY852A restored tRNA ac4C to levels comparable to WT, while the active site mutant ScH545A and the patch 1 and 3 mutants ScR637A and ScR420A did not (Fig. 5C**and** Supplementary Fig. 12). The concordant effects of active site and basic patch mutations on biochemical activity, cellular ac^4^C, and organismal fitness establish that the acetyltransferase activity of NAT10 is the functional determinant of yeast thermoadaptation, and directly link the biochemically characterized residues to modification of both tRNA and rRNA substrates in a living cell.

### Catalytic activity of NAT10 promotes cellular senescence in human cells

**Fig. 6:**
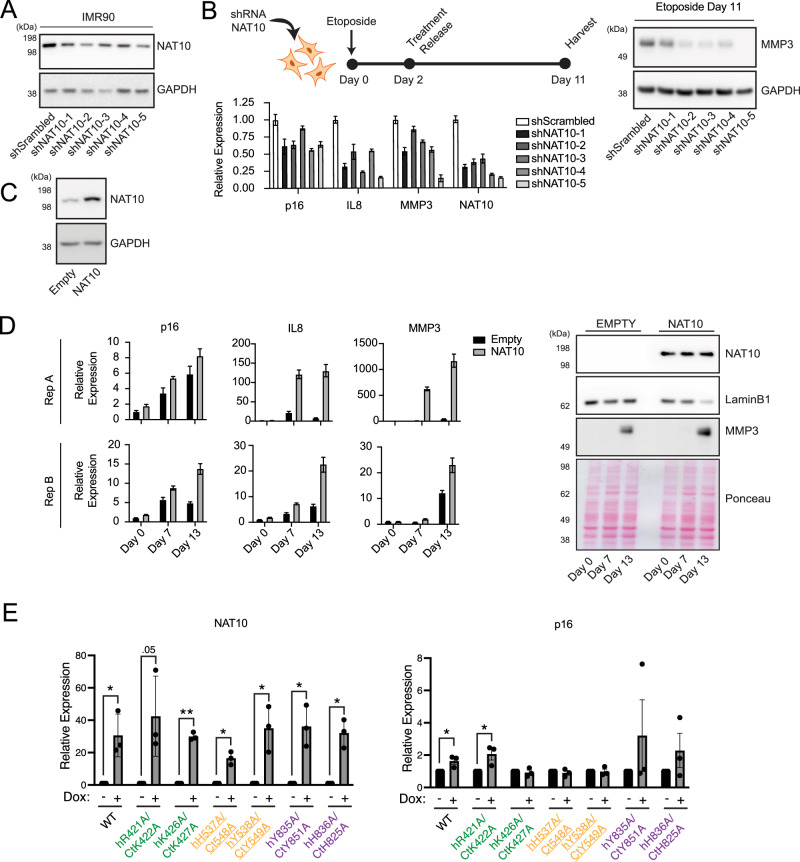
Role of human NAT10 and mutants in cellular senescence in human cells. **A** Immunoblot of IMR90 cells depleted of NAT10 via shRNA. GAPDH is used as a loading control. Representative image is of one of two biological repeats. **B** Top - Schematic of etoposide-induced senescence. NAT10 depleted IMR90 were treated with 100 µM of etoposide for 48 h and recovered in untreated media for an additional 9 days. Bottom - RT-PCR of NAT10 and senescence markers during etoposide induced senescence. Values are normalized to shScrambled control. Error bars represent the standard error of three technical repeats. Right - Immunoblot of MMP3 during etoposide induced senescence. GAPDH is used as a loading control. Error bars represent the standard deviation of three technical replicates. **C** Immunoblot of IMR90 cells overexpressing NAT10 or empty control. GAPDH is used as a loading control. **D** IMR90 overexpressing NAT10 or empty control were treated with 100 µM of etoposide for 48 h and recovered in untreated media and harvested on Day 7 and Day 13. Left - RT-PCR of NAT10 and senescence markers during etoposide induced senescence time course. Values are normalized to Day 0. Error bars represent the standard error of three technical repeats. Two biological replicates are shown Right - Immunoblot of Lamin B1 and MMP3 during etoposide induced senescence. Ponceau is used as a loading control. Error bars represent the standard deviation of three technical replicates. **E** RT-PCR of p16 in IMR90 cells expressing either WT or mutant NAT10 under tetracycline induction. Cells were treated with and without doxycycline and undergo etoposide treatment for 48 h and recovered in undertreated media for an additional 8 days. Values are normalized to non-dox treated cells. Residues are color-coded according to the regions in Fig. 4D. Putative catalytic residues (hH537A/*Ct*H548A and hY538A/*Ct*Y549A) are underlined. Error bars represent the standard deviation of three biological replicates. *p* < 0.05 by paired *t* test.

Previous studies have demonstrated that NAT10 promotes cellular senescence in colorectal cancer cells^47^. To evaluate if putative catalytic and RNA-binding mutants of NAT10 would exhibit a similar phenotype, we utilized an etoposide-induced senescent model in primary lung fibroblast, IMR-90 cells^48^. NAT10 knockdown IMR90 cells were generated using short hairpin RNAs (shRNAs) targeting endogenous NAT10 (Fig. 6A). Interestingly, we found that the depletion of NAT10 decreased etoposide-induced senescence and was accompanied by a reduction in senescence markers p16 and secretory senescence-associated phenotype (SASP) markers IL8 and MMP3 (Fig. 6B). This indicates that attenuation of NAT10 can mitigate senescence in this model system. To further explore this phenomenon, we assessed whether increased expression of NAT10 can exacerbate senescence. We engineered a NAT10 overexpressing cell line (NAT10^WT^-OE) and, upon treatment with etoposide, observed a significant increase in senescence associated with a substantial upregulation of messenger RNA (mRNA) levels of senescence-related genes, including p16, IL8, and MMP3 (Fig. 6C, D). In addition, we observed a distinct decrease in LaminB1 expression, another hallmark of senescence. Taken together, our findings demonstrate a strong positive correlation between NAT10 expression and the regulation of senescence.

Having substantiated NAT10’s ability to promote senescence in this model, we next aimed to evaluate the involvement of NAT10’s acetyltransferase activity in this process. To address this question, we generated IMR90 cell lines with tetracycline-inducible expression of either wild-type hNAT10 or point alanine mutants corresponding to conserved residues identified in our structural and biochemical studies as essential for NAT10 activity. Consistent with the above results, expression of WT-NAT10 showed increased expression of the senescence marker p16. In contrast, the hNAT10 mutants hR421A, hK426A, hH537A, and hY538A (corresponding to *Ct*R421A, *Ct*K427A-patch 3 mutants, *Ct*H548A, *Ct*Y549A-active site mutants) exhibited a markedly decreased p16 expression, while the mutants hY835A and hH836A (corresponding to *Ct*Y851A and *Ct*H852A) in patch 2 recapitulated the effect of WT-NAT10 (Fig. 6E). The results from the human cell-based senescence assays are highly consistent with those from the *Ct*NAT10 in vitro acetylation assays and the yeast cell-based fitness assay, with basic patch 2 mutants scoring as WT or near-WT. Overall, these studies implicate NAT10’s acetyltransferase domain and catalytic activity as a functional driver of cellular senescence.

## Discussion

Eukaryotic NAT10 RNA acetyltransferases are essential for life; however, the contributions of their catalytic and non-catalytic functions to organismal biology remain unclear. Barriers to understanding the catalytic functions have been the inability to prepare intact recombinant NAT10 protein alone in sufficient quantity to study it’s biochemical and structural properties, and our limited understanding of the molecular mechanisms that govern how its multiple domains coordinate catalysis. Here, we report cryo-EM structures of the eukaryotic RNA acetyltransferase *Ct*NAT10 in complex with cytidine-CoA probes, captured both with and without ADP. These structures reveal a striking heart-shaped dimer architecture that fundamentally differs from the L-shaped monomeric form of the bacterial ortholog TmcA^35^. This dimeric arrangement creates a unique active site where the conserved GNAT domain becomes encircled by functional helicase, DUF, and RNA-binding domains from both protomers, reinforcing the importance of the dimeric configuration of NAT10. Interestingly, recent work on programmable RNA acetylation revealed that when a human NAT10 variant lacking its C-terminal region - including the RNA binding domain and dimerization interface – is fused to dCas13 it gains activity toward non-cognate mRNA substrates^49^. This broadened specificity mirrors that of naturally C-terminal truncated archaeal RNA acetyltransferase enzymes^14^ and is consistent with our data indicating that NAT10’s dimeric architecture and RNA binding domains are essential determinants of selective substrate modification.

Our biochemical studies reveal that the THUMPD1 cofactor significantly stimulates cognate tRNA acetylation. Prior literature has demonstrated that THUMP domains employ a conserved molecular ruler mechanism, binding the 3′-CCA terminus to guide target nucleotides to the catalytic site of RNA modification enzymes^50^. Examples include the bacterial thiouridine synthase ThiI^51,52^, which contains an embedded THUMP domain, and the THUMPD3-TRMT112 methyltransferase complex^53^, which relies on trans-acting THUMP domain recognition, similar to NAT10. A recent study by Ma et al. reconstituted yeast Kre33-Tan1 (NAT10-THUMPD1) activity and demonstrated that Tan1’s THUMP and ferredoxin-like domains collectively capture the CCA terminus and 7-bp acceptor stem of tRNA substrates to precisely position the C12 modification site into Kre33’s active site, requiring a 5’-CCG-3’ motif, and correct D-arm orientation^54^. Our findings complement this finding by demonstrating that trans-acting THUMPD1 primarily enhances NAT10 catalytic activity rather than increasing tRNA binding affinity. In other studies, it has been observed that NAT10 binds RNA promiscuously^55^, can acetylate non-cognate mRNA substrates when overexpressed^10^ and maintains reduced tRNA modification activity in the absence of CCA binding^54^, suggesting NAT10 is able to sample a range of RNA-binding conformations. It is tempting to speculate that THUMPD1 acts as a positioning clamp that selectively stabilizes catalytically competent binding modes, converting low-efficiency binary (NAT10-RNA) acetylation complexes to high-efficiency ternary ones.

Beyond the trans-acting function of THUMPD1, our data also highlight NAT10 intrinsic molecular determinants that contribute to substrate modification. Structure-based mutagenesis validated the importance of two conserved active site residues (*Ct*H548 and *Ct*Y549) as well as two basic patches on the dimer surface for cognate RNA acetylation. Notably, mutations in two of these patches (patches 1 and 3) significantly reduced catalytic activity without affecting tRNA binding, while patch 2 mutations retained wild-type activity. The molecular basis for these differential effects remains to be determined, but could again reflect roles in efficient substrate positioning. The finding that non-cognate tRNAs, also containing a cognate 5’-CCG-3’ sequence but in a different position, bind NAT10 with similar affinity to cognate substrates but cannot be acetylated further underscores the importance of correct substrate positioning. Structural analysis of a ternary NAT10-THUMPD1-tRNA complex will be necessary to resolve the molecular mechanisms underlying substrate positioning and ac^4^C catalysis.

**Fig. 7:**
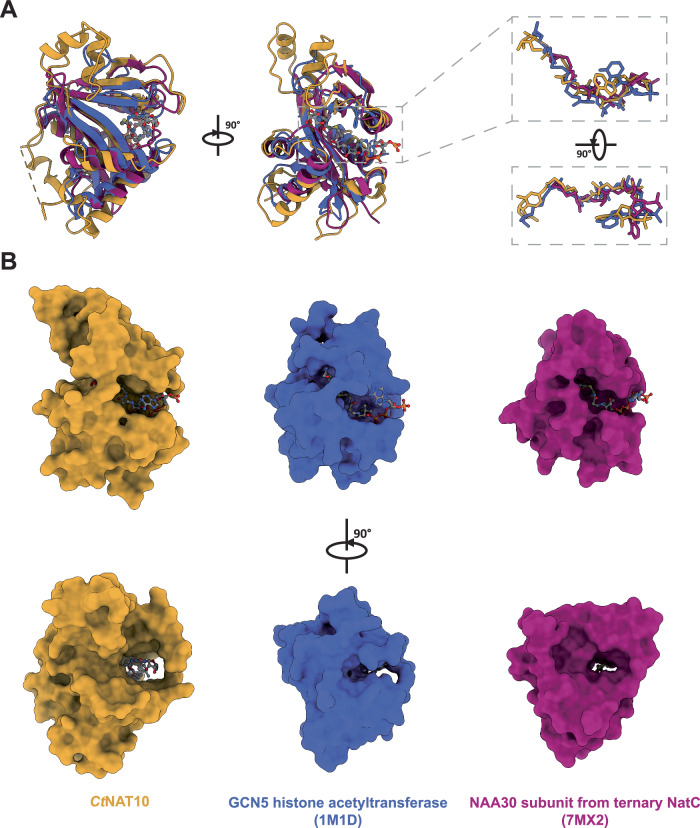
Architecture of the substrate binding tunnel among GNAT family members. **A** Superposition of the GNAT domain of *Ct*NAT10 with representative Histone acetyltransferase (GCN5) and N-terminal acetyltransferase (NAA30) proteins. *Ct*NAT10 (bound to cytidine-amide-CoA) is colored in yellow, *Tetrahymena thermophilus* GCN5 (bound to Lys-CoA, PDB: 1MID^41^) is colored in blue, and human NAA30 (bound to peptide-CoA, PDB: 7MX2^79^) is colored in magenta. In the structure alignment, only cytidine-amide-CoA is shown as a ball-and-stick representation to better demonstrate the bisubstrate CoA binding groove. The right panel shows the conformational comparison among 3 bisubstrate CoA analogs when the Cα of GNAT domains are aligned. **B** GNAT domain surfaces of *Ct*NAT10, GCN5 and NAA30 are shown in both front and side views corresponding to the color code in A, with their in-complexed bisubstrate CoA probes shown as ball-and-stick representation, respectively.

The GNAT domain of *Ct*NAT10, which anchors the cytidine-linked CoA probe, contains the same overall structure of the related GNAT folds as lysine-targeting histone acetyltransferases (HATs) and N-terminal acetyltransferases (NATs). However, a structural superposition of the three folds reveals that the substrate binding tunnel of *Ct*NAT10 is wider than that of HATs and NATs, consistent with the need to acetylate larger and more highly charged RNA substrates (Fig. 7). While our cytidine-CoA probes were designed to capture a transition state intermediate, the structure as observed likely represents a pre-reaction state, since the cytidine portion is partially disordered and the essential catalytic residues (*Ct*H548 and *Ct*Y549) are relatively far from the exocyclic N4 amine. This suggests that significant conformational rearrangements are required to bring the catalytic machinery into proper geometry for N4-cytidine acetylation. The nanoDSF data also demonstrate that tRNA and THUMPD1 binding are also likely insufficient to induce the required conformational changes. Unlike protein acetyltransferases, NAT10 contains an adjacent helicase domain, and our biochemical studies, as well as prior studies of bacterial (TmcA)^35,56^ and archaeal (TK0754)^14^ NAT10 enzymes, have all observed an absolute requirement for ATP in the acetylation reaction. Biochemical analyses of bacterial TmcA are consistent with a mechanism whereby ATP hydrolysis remodels the tRNA anticodon region to deliver the target cytidine for acetylation. We speculate that NAT10 may employ an analogous mechanism, where ATP hydrolysis drives coordinated structural rearrangements that position the catalytic residues His548 and Tyr549 closer to the substrate while simultaneously remodeling the RNA to expose cytidine nucleotides for modification.

Our ability to identify NAT10 mutants that were impaired in catalytic activity in vitro provided an opportunity to dissect the specific contribution of acetyltransferase activity to phenotypes, including yeast growth under heat stress and markers of cellular senescence in human cells. Critically, alanine mutation of two residues in the active site important for NAT10 catalysis in vitro (*Ct*H548 and *Ct*Y549) caused temperature sensitivity in yeast and blocked senescence in human cells, phenocopying NAT10 deletion in both systems. Given the known non-catalytic role of NAT10 in ribosome biogenesis, these results point to the particular importance of the catalytic cytidine N4 acetylation activity of NAT10 in driving these phenotypes. These studies further demonstrate that the ribosome biogenesis activity of NAT10 is not sufficient for maintaining the fungal thermoadaptation and mammalian cellular senescence processes studied here. While the specific tRNA and rRNA substrates (or both) or pathways that link the cytidine N4 acetylation activity of NAT10 to these phenotypes is beyond the scope of this study, these findings highlight the potential utility of targeting NAT10’s catalytic domain in aging-associated diseases such as Hutchinson-Gilford Progeria Syndrome, where selective inhibition of acetyltransferase activity could provide clinical benefit while preserving essential non-catalytic functions.

## Methods

### *Ct*NAT10 expression and purification

A gene encoding N-terminal 6xHis tagged WT-*Ct*NAT10 was synthesized, and codon optimized for overexpression in both *Sf9* and bacteria (BioBasic). Recombinant *Ct*NAT10 protein was expressed in both *Sf9* cells (ThermoFisher, cat #12659017) and homemade bGroESL cells (See **Experimental Model and Subject Details**). For *Ct*NAT10 expression in *Sf9*, the synthesized gene was subcloned into a pFastBacHT A vector (WT-*Ct*NAT10-pFastBacHT A plasmid). High-density (2 × 10^6^ cells * ml ^−1^) suspension cultures of *Sf9* cells were infected at a multiplicity of infection of ~ 1, and incubated for 48 h in Fernbach Shake flasks at 27 °C. All subsequent purification steps were carried out at 4 °C. Cell pellets were harvested and per liter of cell culture was resuspended in 50 ml lysis buffer containing 25 mM Hepes-NaOH pH 7.5, 500 mM NaCl, 10% (v/v) glycerol, 10 mM β-mercaptoethanol (β-ME), and an EDTA-free protease inhibitor tablet (Roche). After mild sonication (2-minute sonication time per liter of cell culture, 5 s “on”/30 s “off” at 40% amplitude, with sonication cup fully buried in ice), 2500 U benzonase nuclease (MilliporeSigma), 200 U RNAse cocktail enzyme mix (Invitrogen) and 0.25 mmol MgCl_2_ were added per liter of cell culture. To ensure that RNAse and benzonase nuclease could sufficiently remove endogenous nucleic acids, the lysate was incubated on a stir plate for 30 min. The cell lysate was then clarified with centrifugation and the supernatant was collected and loaded onto a gravity column manually loaded with TALON Metal Affinity Resin (Takara), washed 5 times with 10 column volumes (CV) of wash buffer containing 25 mM Hepes-NaOH pH = 7.5, 500 mM NaCl, 15 mM imidazole, 1 mM tris-(2-carboxyethyl)phosphine (TCEP), and eluted with elution buffer containing 25 mM Hepes-NaOH pH = 7.5, 500 mM NaCl, 200 mM imidazole, 1 mM TCEP. The eluent was concentrated using a 100 kDa MWCO centrifugal filter (Amicon Ultra, MilliporeSigma) and loaded onto a Superdex 200 Increase 10/300 GL gel filtration column (GE Healthcare) in sizing buffer containing 25 mM Hepes-NaOH pH = 7.5, 300 mM NaCl, and 1 mM TCEP. Only peak fractions with UV_260/280_ < 0.8 were collected and concentrated to ~ 1 mg/ml determined by Nanodrop 2000 (Thermo Fisher Scientific), and aliquoted into 20 μl aliquots and flash-frozen in LN_2_ for storage at − 80 °C until use.

For *Ct*NAT10 *E. coli* overexpression, the synthesized gene was subcloned into a pET30a(+) expression vector. Transformed cells were cultured at 37 °C until the optical density reached an 600 nm (OD_600_) of ~ 0.8, induced with 0.5 mM Isopropyl β-D-1-thiogalactopyranoside (IPTG) at 17 °C, and grown overnight (16-20 h). The cell pellet was harvested and resuspended in lysis buffer containing 25 mM Hepes-NaOH pH = 7.5, 500 mM NaCl, 10% (v/v) glycerol, 10 mM β-mercaptoethanol (β-ME), and an EDTA-free protease inhibitor tablet (Roche). The protein was purified to homogeneity using a combination of TALON Metal Affinity Resin (Takara) and gel filtration chromatography, essentially as described above for *Sf9*-expressed WT-*Ct*NAT10.

The WT-*Ct*NAT10-pFastBacHT A plasmid was used to prepare all *Ct*NAT10 mutants using the QuikChange protocol (Stratagene)^57^. All mutant proteins were overexpressed and purified to homogeneity following the same procedure described above for *Sf9*-expressed WT-*Ct*NAT10. Recombinant *Ct*NAT10 proteins produced from both *E. coli* and *Sf9* expression systems were analyzed by LC-MS/MS, and no significant differences in post-translational modifications (PTMs) were observed.

### *Ct*THUMPD1 expression and purification

The synthesized gene encoding *Ct*THUMPD1 was subcloned into a homemade pCDF GST expression vector (containing a Tobacco-etch virus (TEV)-cleavable N-terminal GST-tag). The plasnid was used to transform BL21 (DE3) competent *E. coli* cells, which was then cultured at 37 °C until the optical density reached an 600 nm (OD_600_) of ~ 0.8, and induced with 0.5 mM IPTG at 18 °C, and grown overnight (16–20 h). The cell pellet was harvested and resuspended in lysis buffer containing 50 mM Hepes-NaOH pH = 7.5, 500 mM NaCl, 10% (v/v) glycerol, 0.1% (v/v) Triton X-100, 10 mM β-ME, and an EDTA-free protease inhibitor tablet (Roche). After sonication (1.5 min sonication time per liter of cell culture, 10 s “on”/30 seconds “off” at 70% amplitude, with sonication cup fully buried in ice), 200 U RNAse cocktail enzyme mix (Invitrogen) and 0.25 mmol MgCl_2_ were added per liter of cell culture. To ensure that RNAse could sufficiently remove endogenous nucleic acids, the lysate was incubated on a stir plate for 30 min. The cell lysate was then clarified with centrifugation, and the supernatant was collected and loaded onto a gravity column manually loaded with Pierce™ Glutathione Agarose (ThermoFisher), washed 5 times with 10 column volumes (CV) of wash buffer containing 25 mM Hepes-NaOH pH = 7.5, 300 mM NaCl, 10 mM β-ME. 50 μl homemade 5 mg/ml TEV protease was diluted with 5 ml of wash buffer, then mixed well with the affinity resin and incubate in 4 °C overnight for the on-column cleavage performing. The following day, the protein was eluted with 5 CV of wash buffer and subsequently diluted with twice the elution volume of dilution buffer (25 mM Hepes-NaOH pH = 7.5, 10 mM β-ME), resulting in a final NaCl concentration of 100 mM. Ion-exchange was carried out using an SP ion-exchange column (GE Healthcare) with a salt gradient (100–1000 mM NaCl). Peak fractions were concentrated to ~ 0.5 mL with a 30 kDa concentrator (Amicon Ultra, Millipore), and loaded onto a Superdex 200 Increase 10/300 GL gel filtration column (GE Healthcare) in sizing buffer containing 25 mM Hepes-NaOH pH = 7.5, 300 mM NaCl, 10% (v/v) glycerol and 1 mM TCEP. Only peak fractions with UV_260/280_ < 0.8 were collected and concentrated to ~ 15 mg/ml determined by Nanodrop 2000 (Thermo Fisher Scientific), and aliquoted into 5 μl aliquots and flash-frozen in LN_2_ for storage at − 80 °C until use.

### Synthesis of cytidine-CoA structural probes



#### N4-bromoacetylcytidine

To a solution of 2-bromoacetic acid (187 mg, 0.71 mmol) in DMF (3 ml) was added diisopropylcarbodiimide (119 μl, 0.71 mmol). After stirring for 5 min at 23 °C, cytidine (187 mg, 0.71 mmol) was added, and the reaction mixture was stirred for another 45 min. DMF was removed in vacuo to provide the crude product as a light pink residue, which was purified by silica flash chromatography (8% MeOH in DCM) to yield N4-bromoacetylcytidine as a white solid (107 mg, 38% yield). ^1^H NMR (500 MHz, DMSO) δ 11.23 (s, 1H), 8.48 (d, J = 7.5 Hz, ^1^H), 7.12 (d, J = 7.4 Hz, 1H), 5.78 (d, J = 3.0 Hz, 1H), 4.14 (s, 2H), 4.02 – 3.94 (m, 2H), 3.90 (dt, J = 6.0, 2.9 Hz, 1H), 3.74 (dd, J = 12.2, 2.9 Hz, 1H), 3.60 (dd, J = 12.3, 3.0 Hz, 1H), 3.17 (s, 4H). LRMS (ESI/Single Quad) calcd for C_11_H_15_BrN_3_O_6_^+^ [M + H]^+^ 364.0, found 364.1.



#### Cytidine-amide-CoA

To a solution of N4-bromoacetylcytidine (25 mg, 68 μmol) in DMSO (85 μl) was added coenzyme A (26 mg, 34 μmol) in phosphate buffer (100 mM, 85 μl, pH 7.0). The reaction mixture was stirred at 23 °C for 2.5 h, at which time CoA had been consumed as monitored by HPLC. The crude reaction mixture was diluted with aqueous triethylammonium acetate (TEAA, 0.5 ml, 20 mM, pH 7), and then purified by C18 flash chromatography [Solvent A: TEAA (20 mM, pH 7); Solvent B: acetonitrile. Gradient: 0% B for 2 column volumes (CV), 0% to 15% B over 12 CV, 15% B for 10 CV, 15% to 30% B over 10 CV, 30% to 50% B over 1 CV, and 50% B over 5 CV] to yield the bisubstrate conjugate as a white solid (5.5 mg, 15% yield). ^1^H NMR (500 MHz, D_2_O) δ 8.53 (s, 1H), 8.31 (d, J = 7.6 Hz, 1H), 8.23 (s, 1H), 7.21 (d, J = 7.5 Hz, 1H), 6.13 (d, J = 6.6 Hz, 1H), 5.88 (d, J = 2.5 Hz, 1H), 4.63 – 4.50 (m, 1H), 4.34 – 4.28 (m, 1H), 4.23 (d, J = 3.8 Hz, 2H), 4.19 (d, J = 3.0 Hz, 2H), 4.03 (s, 1H), 4.01 (dd, J = 12.8, 1.9 Hz, 1H), 3.89 – 3.80 (m, 2H), 3.55 (dd, J = 9.7, 4.7 Hz, 1H), 3.51 – 3.43 (m, 3H), 3.38 (t, J = 6.5 Hz, 2H), 2.76 (t, J = 6.6 Hz, 2H), 2.45 (t, J = 6.5 Hz, 2H), 1.92 (s, 6H), 0.89 (s, 3H), 0.76 (s, 3H). 31 P NMR (202 MHz, D_2_O) δ 1.83, − 10.90 (d, J = 20.8 Hz), − 11.48 (d, J = 20.9 Hz). LRMS (ESI/Single Quad) calcd for C_32_H_50_N_10_O_22_P_3_S^+^ [M + H]^+^ 1051.2, found 1050.6. While the resulting compound is N4-acetylcytidine-amide-CoA, we refer to it in the text as Cytidine-amide-CoA for simplicity.



#### Cytidine-acetone-CoA

To a solution of bromoacetonyl-thiouridine (25 mg, 63 μmol) in DMSO (85 μl) was added coenzyme A (49 mg, 63 μmol) in phosphate buffer (100 mM, 3 ml, pH 7.0). The reaction mixture was stirred at 23 °C for 15 min. The crude reaction was then diluted with 0.1 % trifluoroacetic acid (TFA) and purified by reverse phase HPLC (0.1% TFA/CH_3_CN) to yield the acetone-linked CoA probe. ^1^H NMR (500 MHz, D_2_O) δ 8.57 (s, 1H), 8.34 (s, 1H), 8.22 (s, 1H), 6.68 (s, 1H), 6.16 – 6.09 (m, 1H), 5.75 (d, *J* = 2.4 Hz, 1H), 4.82 – 4.72 (m, 3H), 4.52 – 4.48 (m, 1H), 4.16 (dddd, *J* = 17.0, 11.7, 4.8, 2.9 Hz, 3H), 4.12 – 3.99 (m, 2H), 3.93 (s, 1H), 3.88 (dd, *J* = 12.9, 2.2 Hz, 1H), 3.80 – 3.65 (m, 2H), 3.63 (s, 2H), 3.56 – 3.47 (m, 1H), 3.38 (t, *J* = 6.5 Hz, 2H), 3.24 (t, *J* = 6.6 Hz, 2H), 2.59 (t, *J* = 6.6 Hz, 2H), 2.37 (t, *J* = 6.5 Hz, 2H), 0.84 (s, 2H), 0.70 (s, 3H). ^13^C NMR (126 MHz, D_2_O) δ 177.31, 174.70, 173.92, 149.85, 148.47, 144.63, 142.48, 141.75, 118.52, 91.69, 87.42, 83.82, 83.49, 74.40, 74.18, 74.14, 74.08, 73.92, 71.90, 68.34, 65.08, 59.88, 39.15, 38.35, 38.28, 38.06, 35.41, 35.32, 31.34, 30.82, 20.84, 18.16. LRMS (ESI/Single Quad) calcd for C_33_H_51_N_9_O_22_P_3_S_2_^+^ [M + H]^+^ 1082.2, found 1082.8. While the resulting compound is thiouridine-acetonyl-CoA, we refer to it in the text as Cytidine-acetone-CoA for simplicity.

### Differential scanning fluorimetry assay

To investigate the thermal stability of *Ct*NAT10 alone or in the presence of different cofactors, individual 20 μl reactions were prepared containing 1 μM of *ct*NAT10 and a 1:1000 dilution of a 5000x concentrated SYPRO orange dye (Thermo Fisher Scientific) in DSF buffer (37.5 mM Hepes-NaOH pH = 7.5, 150 mM NaCl, 6 mM KCl, 5 mM MgCl_2_, 0.5 mM DTT, 0.5 mM TCEP). Three replicate samples were prepared for each combination of protein and additives with the indicated concentration in the figure legends, and distributed to separate wells in a 384-well assay plate specifically designed for real-time qPCR measurements. Thermal melting curves were obtained by heating the samples from 20 to 95 °C using a ramping rate of 2%, with fluorescence detection at 580 nm, monitored using a 7900HT Fast Real Time PCR System (Applied Biosystems). The fluorescence was normalized based on the peak value, and Tm was defined as the temperature at the inflection point of the SYPRO fluorescence curve. To assess the variability of the results, we calculated the standard deviation of each condition and plotted error bars. Plot generation and *P*-values calculation was performed using GraphPad Prism version 9 for Windows, GraphPad Software, Boston, Massachusetts USA, www.graphpad.com.

### Nano differential scanning fluorimetry (nanoDSF) assay

To assess the thermal stability of *Ct*NAT10 in the absence or presence of tRNA and THUMPD1, 2 µM *Ct*NAT10 was pre-incubated at 4 °C for 1 h with the indicated combinations of tRNA or THUMPD1. When included, the final concentrations of added components were 5 µM *Ct*THUMPD1, 10 µM *Ct* tRNA (+/− 1 mM ATPɣS and 2 mM Ac-CoA). The assay buffer consisted of 50 mM HEPES-KOH (pH 7.6), 25 mM NaCl, 12 mM KCl, 10 mM MgCl₂, and 5 mM TCEP. For nanoDSF assays, 10 µl of each sample was loaded into high-sensitivity capillaries (NanoTemper Technologies) and placed in the Prometheus Panta instrument (NanoTemper Technologies). Samples were subjected to a thermal ramp from 15 to 95 °C at a rate of 1.0 °C/min with 68% LED excitation, as predetermined by a discovery scan. Data from each channel were analyzed using the PR.Panta Analysis software (version 1.6.3) to determine the melting temperature (Tm) under each condition. Three technical replicates were performed per condition. Final Tm plots were generated with GraphPad Prism version 9 for Windows (GraphPad Software, Boston, MA, USA; www.graphpad.com.).

### In vitro tRNA transcription

Predicted mature tRNA sequences were retrieved from GtRNAdb and converted into their corresponding genomic sequences to serve as DNA templates^58,59^. DNA fragments were synthesized as two overlapping oligonucleotides (forward primer containing the T7 promoter sequence and a reverse primer; Integrated DNA Technologies, IDT). The oligonucleotides were annealed and extended by PCR using 25 μl of PfuUltra II Hotstart PCR Master Mix (Agilent Technologies) with 5 μM of each primer in a total reaction volume of 50 μl. The PCR program: initial denaturation at 95 °C for 5 min, followed by 40 cycles of denaturation at 95 °C for 50 s, annealing at (Tm − 5) °C for 1 min, elongation at 72 °C for 30 s, and a final extension at 72 °C for 5 min. The extended primers were resolved on a 3% agarose gel, and the desired DNA bands were excised and recovered by gel extraction. The purified DNA was subsequently used as a template for a second round of PCR with shorter, non-overlapping primers under similar cycling conditions. The final PCR products were purified using the QIAquick PCR Purification Kit (QIAGEN) and served as in vitro transcription templates.

In vitro transcription was performed using the MEGAscript™ T7 Transcription Kit (Invitrogen) according to the manufacturer’s instructions. Reactions were incubated at 37 °C for 14 h. To remove unincorporated nucleotides and other impurities, RNA products were purified using the RNA Clean & Concentrator Kit (RCC-25, Zymo Research) following the manufacturer’s protocol. The quality of the transcribed tRNA was assessed by electrophoresis on 10% Novex™ TBE-Urea gels (Thermo Fisher Scientific) in 1 × TBE buffer, followed by staining with SYBR Gold (Invitrogen). Imaging was performed using a ChemiDoc Imaging System (Bio-Rad). For refolding, tRNA samples were heated at 95 °C for 15 min and gradually cooled to room temperature. Refolded RNAs were immediately flash-frozen in liquid nitrogen and stored at − 80 °C until further use.

### Electrophoretic mobility shift assays (EMSA)

For testing the purified *Ct*NAT10 wild-type binding with synthesized tRNA and rRNA substrates, all the RNA probes were synthesized, purified by RNase-free HPLC, and dissolved in DNase/RNase-free water at 1 mM by Genescript (for sequence detail see **Key Resources** Supplementary Table S1). All RNA probes were reannealed before utilizing for EMSA. To conduct binding assays, samples containing 0.5 μM of the indicated RNA substrate with various concentrations (indicated in the figure legends) of purified *Ct*NAT10 proteins were incubated overnight (15 h) at 4 °C in a buffer containing 50 mM Hepes-KOH pH = 7.6, 90 mM NaCl, 12 mM KCl, 10 mM MgCl_2_, 1 mM DTT, 0.3 mM TCEP, 1.0 U/μl SUPERase-In RNAse inhibitor (Invitrogen), 0.5 mM ATP, and 0.1 mM acetyl-CoA. The next day, samples were incubated for 1.5 h at 45 °C, and the entire 10 μl RNA–protein mixture was then loaded onto DNA Retardation Gels (6%) (Invitrogen) with 2.5 μl Novex™ Hi-Density TBE Sample Buffer (5X) (Invitrogen). The gel was run in 0.5x TBE buffer at 120 V for 42 min and stained with 1x SYBR™ Gold Nucleic Acid Gel Stain (Invitrogen) diluted with 0.5x TBE buffer for 20 min. Imaging was performed using a ChemiDoc Imaging System (BIO-RAD), and quantification was carried out using Fiji ImageJ^60^ to measure the intensity of the free RNA band. For protein band visualization, the same gels were subjected to three Milli-Q water washes to remove excess SYBR Gold, and subsequently stained with Coomassie Blue for 20 min, followed by de-staining overnight with agitation in Milli-Q water.

For evaluating *Ct*NAT10 WT/alanine-mutants binding with cognate and non-cognate tRNA substrates (Supplemental Fig. 4A), all the RNA probes were in vitro transcribed as described above (for sequence detail see **Key Resources** Supplementary Table S1). To conduct binding assays for columns 1 and 2, 0.25 μM of the indicated tRNA probes were assembled with various concentrations (indicated in the figure legends) of purified *Ct*NAT10 or *Ct*THUMPD1 proteins; For column 3 samples, *Ct*NAT10 and *Ct*THUMPD1 proteins were pre-assembled in a 1:1 molar ratio and incubated at 4 °C for 2 h. 0.25 μM of the indicated tRNA probes were then assembled with various concentrations (indicated in the figure legends) of *Ct*NAT10 + *Ct*THUMPD1 complex; For column 4–6 samples, 0.25 μM of the indicated tRNA probes were pre-incubated with 0.75 μM of *Ct*THUMPD1 protein on ice for 2 h, then various concentrations (indicated in the figure legends) of *Ct*NAT10 WT/alanine-mutants were titrated into tRNA + *Ct*THUMPD1 complex. All samples were incubated at 37 °C for 1 h in a buffer containing 50 mM Hepes-KOH pH = 7.6, 75 mM NaCl, 12 mM KCl, 10 mM MgCl_2_, 1 mM DTT, 1.0 μ/μl SUPERase-In RNAse inhibitor (Invitrogen), 0.2 mg/ml BSA, 0.5 mM ATP, and 0.1 mM acetyl-CoA. The entire 10 μl RNA–protein mixture was then loaded onto DNA Retardation Gels (6%) (Invitrogen) with 2.5 μl Novex™ Hi-Density TBE Sample Buffer (5X) (Invitrogen). The gel was run in 0.5x TBE buffer at 100 V for 65 min and stained with 1x SYBR™ Gold Nucleic Acid Gel Stain (Invitrogen) diluted with 0.5x TBE buffer for 10 min. The RNA gel imaging and band visualization were carried out essentially as described above.

### In vitro RNA acetylation assay

Reaction mixtures (20 µl) containing 50 mM Hepes-KOH pH = 7.6, 50 mM NaCl, 12 mM KCl, 10 mM MgCl_2_, 5 mM TCEP, 1.0 U/μl SUPERase-In RNAse inhibitor (Invitrogen), 1 mM ATP, 0.1 mM [1-^14^C] acetyl-CoA (PerkinElmer, 60 mCi/mmol), 15 µM indicated tRNA probes, 15 µM recombinant *Ct*THUMPD1 protein (if applicable) and 3 µM recombinant WT or mutant *Ct*NAT10 were incubated at 45 °C for the times indicated in the figure legends. After reaction, the tRNAs were cleaned and extracted by RNA Clean & Concentrator Kit (RCC-25, Zymo Research) following the manufacturer’s protocol. Eluted tRNA were transferred to 4 mL scintillation fluid for signal measurement (Tri-Carb A4810TR scintillation counter, Revvity). GraphPad Prism (version 9.5.1) was used for all data fitting and normalization. The relative activity of *Ct*NAT10 mutants were normalized to the WT-*Ct*NAT10 activity. Three technical replicates were performed for all the activity assays.

### Mass photometry

Mass photometry sample slides were manually assembled by placing a 6-wells silicone cassette onto the sample carrier cover glass. For analysis of NAT10 and THUMPD1 proteins, samples and standard proteins were diluted with dilution buffer (300 mM NaCl, 25 mM Hepes-NaOH pH = 7.5, 1 mM TCEP) at room temperature. The dilution buffer was freshly filtered by a 0.22 µm syringe filter before the experiment. For calibration, 2 μl of homemade BAM-TG protein standard mix (300 nM thyroglobulin, 1 μM beta-amylase in PBS with 0.5% glycerol) was added into the droplet of 18 μl dilution buffer and mixed thoroughly before measurement. Before testing each protein sample, 19 μl dilution buffer was loaded into the sample well for finding focus and quality metrics checking, then 1 μl of 200 nM protein stock was added into the sample droplet of 19 μl dilution buffer and mixed thoroughly before measurement. For anlysis of protein complexes with Leu tRNA, the tRNA was reannealed and assembled with protein by incubating ctRNA^Leu^ (0.25 µM), ctTHUMPD1 (7 µM), and ctNAT10 (7 µM). The incubation conditions for both the complex and the controls were the same as those used for the electrophoretic mobility shift assays (EMSA) described above. Samples were incubated overnight (15 h) at 4 °C in buffer containing 50 mM HEPES-KOH (pH 7.6), 90 mM NaCl, 12 mM KCl, 10 mM MgCl₂, 1 mM DTT, 0.3 mM TCEP, 1.0 μ/µL SUPERase•In RNase inhibitor (Invitrogen), 0.5 mM ATP, and 0.1 mM acetyl-CoA. The following day, samples were incubated for 1.5 h at 45 °C. For the crosslinked sample (XL), the complex was treated with 1% glutaraldehyde for 10 min at room temperature (RT) and then quenched with 750 mM glycine for 10 min at RT. Mass photometry measurements were performed as described above, except that the dilution buffer was adjusted to 50 mM HEPES-KOH (pH 7.6), 90 mM NaCl, 12 mM KCl, 10 mM MgCl₂, and 1 mM DTT. The samples were subjected to mass photometry analysis as described above. For data analysis, a calibration curve was created by manually putting in the molecular weight of the Gaussian fit for each peak of the corresponding standard protein (thyroglobulin: 670 kDa, beta-amylase: 224 kDa and 112 kDa) to make sure the R^2^ value for the calibration curve > 0.999. Movies for each protein sample were acquired in 60 s (3000 frames in total) with all the other settings at the default values. All data collection was performed using a commercial TwoMP mass photometer with the software AcquireMP, version 2022 R1 (Refeyn Ltd.). Data analysis was performed by DiscoverMP (Refeyn Ltd.).

### Cryo-EM sample preparation and data collection

For all *E.coli* and *Sf9* produced *Ct*NAT10 with cytidine-CoA conjugate compound samples, freshly purified protein was first diluted with sizing buffer (25 mM Hepes-NaOH pH = 7.5, 300 mM NaCl, and 1 mM TCEP) to 0.8 mg/ml. For *E.coli* produced *Ct*NAT10 with the cytidine-acetone-CoA conjugate sample, the protein was incubated with 2 mM of the cytidine-acetone-CoA and 5 mM TCEP pH = 7.5 on ice for 1 h; and for both the *E.coli* and *Sf9* produced *Ct*NAT10 with the cytidine-amide-CoA conjugate sample, the protein was incubated with 2.5 mM ATP, 5 mM MgCl_2_, 2 mM of the cytidine-amide-CoA conjugate and 5 mM TCEP pH = 7.5 on ice for 1 h. For the preparation of grids, 3 μl of protein-cofactor complex sample was applied to glow-discharged (PELCO easiGlow™) Quantifoil R1.2/1.3 300 mesh copper grids, and a FEI Vitrobot Mark IV was used for blotting (Time = 2.5 s, force = 3) under 100% humidity at 7 °C, then grids were plunged frozen in liquid ethane. Grid screening was performed with a FEI Titan Krios transmission electron microscope equipped with a K3 Summit direct detector (Gatan) at a magnification of 105,000x, and the most promising grids were used for data collection under the same magnification at super-resolution mode, with defocus values set from − 1.0 to − 3.0 μm. The details of cryo-EM data collection parameters are described in Table 1.

### Cryo-EM data processing and 3D model reconstruction

All three cryo-EM datasets were processed with similar workflows, described as follows. After importing the data into Relion 3.1.3^61^, beam-induced motions were corrected for the original image stacks using MotionCor2^62^, while the pixel was binned 2-fold. After motion correction, all micrographs were imported into Cryosparc2^63^, where they were processed further. CTFFIND4^64^ was used to determine the defocus value of each micrograph, and micrographs with contrast transfer function (CTF) information poorer than 5 Å were discarded. For the particle picking step, blob picker was utilized to pick ~ 50,000 particles from ~ 200 micrographs, with a “blob” diameter of 100-200 Å. After detailed particle inspection, selected particles were used for reference-free 2D classifications, and “good” classes were chosen as a “template” for template picking on entire datasets. About 3,000,000 particles were picked for each dataset and went through several rounds of 2D classification to remove the particles that did not appear to be protein particles. Only the best 2D classes with different orientations were selected, and 80,000 particles were randomly selected by the algorithm and extracted (binned 2-fold again at the extraction step) to perform Ab-initio modeling.

For 3D models reconstruction, 4 initial models were created using Ab-initio modeling. All the “good” particles that were selected by 2D classifications went through two rounds of heterogeneous refinement using all 4 initial models as input volume, to further remove junk particles. After a reasonable volume class was obtained, the particles were re-extracted without binning and unbinned particle stacks were then used for all high-resolution refinement steps, including homogeneous refinement and final non-uniform refinement (Nu-refinement), to obtain the final high-resolution structures. Maps with overall resolutions of 3.37 Å, 3.56 Å and 3.37 Å were generated for *E.coli* produced recombinant *Ct*NAT10/cytidine-acetone-CoA, *E.coli* produced recombinant *Ct*NAT10/cytidine-amide-CoA/ADP and *Sf9* produced recombinant *Ct*NAT10/cytidine-amide-CoA/ADP datasets, respectively, with no symmetry imposed during initial homogeneous refinement. The map resolutions were further improved to 3.35 Å, 3.48 Å and 3.23 Å after non-uniform refinement^65^, per-particle global CTF refinement and local CTF refinement. C2 symmetry was imposed only at the final non-uniform refinement step. This decision was based on a detailed inspection of the homogeneous refinement map in C1, which revealed no significant differences between the two halves of the dimer in the densities for ATP/ADP and the bisubstrate probes. On this basis, we applied C2 symmetry in the final step to obtain an improved overall map. Furthermore, the C1 reconstruction indicates that both ATP/ADP and acetyl-CoA are bound simultaneously in both monomers. The final maps with overall resolutions of 3.29 Å, 3.19 Å and 3.02 Å were generated with C_2_ symmetry applied.

### Cryo-EM model building and refinement

For the *E.coli* produced recombinant *Ct*NAT10/cytidine-acetone-CoA dataset, the density map was reconstituted from 160,385 particles chosen from 5500 raw electron micrographs, and the *Ec*TmcA crystal structure (PDB: 2ZPA) was utilized as the initial model for building the *Ct*NAT10 structure. The residues in the *Ec*TmcA structure model were first mutated to corresponding residues of *Ct*NAT10 according to sequence alignment by CHAINSAW^66,67^ (CCP4 supported program^68^), then fit into the map as a rigid body utilizing UCSF Chimera^69^. The atomic coordinates was manually built using COOT^70^ according to the cryo-EM map, while guided by the secondary structure prediction from XtalPred^71^. For cytidine-acetone-CoA ligand model building, the initial chemical structure was manually depicted by “Ligand Builder” in COOT^70^ and roughly placed into the map based on the density. The geometry restraint information was generated by phenix.elbow^72^, and the information was provided to the phenix.real_space_refine program during protein structure auto-refinement. The complete model was auto refined using real-space refinement in PHENIX then manually adjusted with COOT^70^ according to the model evaluation by MolProbity^73^ for several rounds, until no significant outliers were reported in the phenix.real_space_refine program. The structure is well ordered with all four functional domains well resolved, except for residues 1-5, 63-93, 437–466, 498–510, 667–704, 929–1073, which were not visible in the cryo-EM map.

For the *E.coli* produced recombinant *Ct*NAT10/cytidine-amide-CoA/ADP and *Sf9* produced recombinant *Ct*NAT10/cytidine-amide-CoA/ADP datasets, the structure were build using the *E.coli* produced *Ct*NAT10/cytidine-acetone-CoA structure as the starting model, and refined as described above. Both structures are well ordered. For *E.coli* produced recombinant *Ct*NAT10/cytidine-amide-CoA/ADP structure, residues 1-5, 63–93, 437–466, 498–510, 667–695 and 929–1073, were not visible in the cryo-EM map. For *Sf9* produced recombinant *Ct*NAT10/cytidine-amide-CoA/ADP structure, residues 1–4, 64–93, 437–466, 498–510, 667–704 and 930–1073, were not visible in the cryo-EM map.

All representations of cryo-EM density and structural models were prepared with UCSF ChimeraX^74^ and PyMol^75^. The dimer interface area between the two helicases domain and RNA binding domain were calculated using ‘Protein interfaces, surfaces and assemblies’ service PISA at the European Bioinformatics Institute. (http://www.ebi.ac.uk/pdbe/prot_int/pistart.html)^76^.

### Yeast cell transformation

Transformation of the parent strain (Δkre33 + pSH35-C, which harbors a genomic KRE33 deletion complemented by a catalytically deficient H545A allele on a URA3-marked plasmid) was performed with each HIS3-marked plasmid construct as previously described^77^. Briefly, a single colony of the parent strain was inoculated in 5 ml of YPD medium and incubated on a rotary shaker at 200 rpm at 30 °C for 16–20 h. To a flask containing 50 ml YPD medium, 2.5 × 108 cells (a suspension containing 1 × 107 cells ml^−1^ will give an OD_600_ of 1) were added to obtain a titer of 5 × 106 cells ml^−1^, and the flask was incubated at 30 °C, 200 rpm until the titer reached at least 2 × 107 cells ml^−1^. Cells were harvested by centrifuging for 5 min at 3000 × *g*. The pellet was washed 2 times by resuspending in 25 ml sterile water and centrifuging for 5 min at 3000 × *g*. The pellet was then resuspended in 1 ml of sterile water and transferred into a 1.5 ml Eppendorf tube. Water was removed by centrifuging for 30 sec at 13000 × *g*, and the pellet was resuspended again in 1 ml of water. From this, 100 µl (10^8^ cells) was pipetted into 1.5 ml Eppendorf tubes, one for each transformation, and centrifuged at 13000 × *g* for 30 sec to remove the supernatant. The transformation mix (240 µl of PEG3350 (50% w/v), 36 µl of 1 M LiAc, 50 µl single-stranded salmon sperm carrier DNA (2 mg ml^−1^), and 34 µl plasmid DNA ( ~ 500 ng) in sterile water) was added to each cell pellet and mixed by vortexing. A transformation was done without a plasmid as a control. After incubation at 42 °C for 40 min, cells were pelleted, resuspended in 200 µl YPD medium, and spread on SC-His plates to select for transformants carrying the plasmid of interest. Plates were incubated at 30 °C incubator for 3-4 days until colonies appeared. All transformations (WT KRE33, H545A, and other mutants) were performed using the same parent strain, allowing direct comparison between KRE33 variants. Of note, “H545A” refers to the Δkre33 + ScH545A parent strain transformed with a plasmid carrying an additional copy of the H545A allele. We deliberately used a plasmid encoding the same H545A substitution rather than an empty vector to maintain consistency across experimental conditions, in which all strains were transformed with a plasmid expressing a Kre33 variant. Importantly, the plasmid-encoded H545A allele is distinguishable from the chromosomally encoded version by a silent codon difference (GCT versus GCA for Ala at position 545), allowing confirmation of plasmid expression. This design ensured that any observed phenotypes could be directly attributed to the biochemical properties of the expressed variants. Successful transformation was confirmed by PCR amplification and Sanger sequencing of the mutated region of each plasmid, which was isolated using the Zymoprep yeast plasmid miniprep II kit (Zymo Research). For H545A transformants, genomic and plasmid-based H545A alleles were distinguished by PCR based on the presence of different codons for alanine 545 (GCA in the genomic copy versus GCT in the plasmid).

### Yeast drop test assay

Yeast cells were grown in YPD medium at 30 °C until they reached an OD_600_ of 1 or higher. Starting with the OD 1 culture, a 1:10 dilution series was prepared for each yeast strain. From each dilution (1, 1:10, 1:100, and 1:1000), 2 µl was spotted onto two YPD plates, and each was incubated at 30 °C and 37 °C for 48–72 h. Images were taken to compare the growth of each strain at 30 °C versus 37 °C.

### Yeast total RNA extraction with hot acidic phenol method

Yeast cells were grown overnight in 5 ml of YPD medium and pelleted by centrifugation at 1500 g for 3 min. Total RNA was extracted from the cell pellets using the hot acidic phenol method, as previously described^78^. In brief, the cell pellets were resuspended in 1 ml of ice-cold water and transferred to a clean 1.5 ml Eppendorf tube. The cells were pelleted again by centrifugation at 1500 × *g* for 3 min at 4 °C. The pellet was then resuspended in 400 µl of TES buffer (10 mM Tris-HCl pH = 7.5, 10 mM EDTA, 0.5% SDS), and 400 µl of preheated (65 °C) acid phenol was added. The mixture was vortexed vigorously for 10 sec and then incubated for 60 min at 65 °C with brief vortexing every 15 min for 10 sec. After 60 min, the tubes were placed on ice for 5 min and then centrifuged for 5 min at 21,000 × *g* and 4 °C. The top aqueous layer was transferred to a clean 1.5 ml tube, and 400 µl of acidic phenol was added. The mixture was vortexed vigorously, kept on ice for 5 min, and then centrifuged for 5 min at 21,000 × *g* and 4 °C. The aqueous layer was transferred to a clean tube containing 400 µl of chloroform, vortexed vigorously, and then centrifuged for 5 min at 21,000 × *g*. Finally, the top aqueous layer, which contained the RNA, was transferred to a new tube. Ethanol precipitation was carried out by adding 40 ml of 3 M sodium acetate (pH = 5.3) and 1 ml of ice-cold 100% ethanol, incubating the mixture at − 20 °C overnight, and then centrifuging for 30 min at 21,000 × *g* and 4 °C. The pellet was washed with 70% ice-cold ethanol, dried, and resuspended in H_2_O.

### Ac4C analysis on yeast rRNA and tRNA^Ser^

Total RNA (5 µg) from each yeast transformant was treated with TURBO DNase (Invitrogen) by adding 1 µl of the enzyme, 5 µl of 10X buffer, and H_2_O in a 50 µl total volume and incubating at 37 °C for 1 h. RNA was then isolated using the Zymo RNA Clean and Concentrator-5 kit (Zymo Research) according to the manufacturer’s protocol.

Nucleotide resolution sequencing of ac4C was performed as previously described^17^. DNase-treated RNA samples (1 µg) were treated with 100 mM NaCNBH_3_ or vehicle (H_2_O) in a final reaction volume of 100 μl. Reactions were initiated by the addition of 1 M HCl to a final concentration of 100 mM and incubated for 20 min at room temperature. Reactions were stopped by neutralizing the pH with 30 μl 1 M Tris-HCl (pH 8.0). Reaction volumes were adjusted to 200 μl with H_2_O, ethanol precipitated, and quantified using a Nanodrop 2000 spectrophotometer.

For 18S rRNA h45 analysis, yeast total RNA from individual reactions (200 pg) was incubated with h45 reverse primer (4.0 pmol,) in a final volume of 20 μl, heated to 65 °C for 5 min, and transferred to ice for 3 min. Reverse transcription was performed in SuperScript III reaction buffer (Invitrogen) with 5 mM DTT, 200 units SuperScript III RT, and 500 μM dNTPs (250 μM dGTP) at 55 °C for 60 min, then quenched at 70 °C for 15 min.

For tRNA^Ser^ AGA analysis, RNA from individual reactions was heated to 65 °C for 5 min with tRNA^Ser^ AGA RT primer (10 pmol; TGGCGACAACTGCAGGACTC), 1.5 µL of 10 mM dNTPs, and nuclease-free water (total 13.5 µL), then transferred to ice for 3 min. Reverse transcription was performed with 1 × Induro buffer, 200 units Induro RT, 8 units RNasin, and nuclease-free water (final volume 20 µL) at 37 °C for 18 h, then terminated at 95 °C for 1 min.

For both targets, cDNA (2 μl) was amplified in 50 μl PCR reactions using Phusion Hot Start Flex DNA polymerase (New England Biolabs) with 1 × HF buffer, 2.5 pmol each primer (h45: forward CGTCGCTAGTACCGATTGAATGG, reverse TAATGATCCTTCCGCAGGTTCACCTAC; tRNA^Ser^ AGA: forward GCATATNNNNNNNNTGTATAAGGGCAACTTG, reverse GATGATNNNNNNNNTGGCGACAACTG; Ns indicate barcodes for sample pooling), 200 μM each dNTP, and 2 units enzyme. PCR products were resolved on 2% agarose gels, stained with SYBR Safe, visualized under UV (302 nm), excised, extracted using the QIAquick Gel Extraction Kit (Qiagen), and submitted for Sanger sequencing (Azenta) using the forward (h45) or reverse (tRNA^Ser^ AGA) PCR primer. Sequencing chromatograms were analyzed using 4Peaks software. Percent misincorporation was calculated as: (Peak intensity of T) / (Sum of C and T base peaks) × 100%.

### Human cell line culture and etoposide induction

IMR90 (Primary human lung fibroblast) and HEK293T (Human kidney cell line) were described previously. IMR90 and HEK293T cells were cultured in DMEM supplemented with 10% fetal bovine serum (FBS) and 100 units ml^−1^ penicillin and 100 µg ml^−1^ streptomycin (Invitrogen). IMR90 cells were cultured under physiological oxygen (3%). IMR90 cells with a population doubling of less than 40 were used for experiments. For etoposide-induced senescence, IMR90 cells were transiently treated with 100 μM of etoposide (Sigma-Aldrich Catalog# E1383) for 48 h. Cells were replaced with untreated media and assayed at designated time points.

### Immunoblot

IMR90 cells were lysed in buffer containing 20 mM Tris, pH = 7.5, 137 mM NaCl, 1 mM MgCl_2_, 1 mM CaCl_2_, 1% NP-40, 10% glycerol, and supplemented with Halt Protease Inhibitor Cocktail (ThermoFisher #78430) and Benzonase (MilliporeSigma #70746-3). Cells were incubated at 4 °C, shaking for at least 1 hour. Sodium dodecyl sulfate (SDS) was added to a final concentration of 1% and boiled for 10 min. Samples were centrifuged at 13000 RPM and the supernatant (lysate) was quantified for protein concentration. Lysates were subjected to electrophoresis and transferred to a 0.2 μm nitrocellulose membrane. The membrane was blocked with 5% Milk for at least 30 min shaking and rinsed 3 times using Tris-buffered saline (TBS) supplemented with 0.1% Tween (TBS-T). The membrane was incubated overnight at 4 °C with primary antibodies (See **Key Resource** Supplementary Table S4) in TBS-T in 5% Bovine Serum Albumin (BSA) and probed with horseradish peroxidase-conjugated secondary antibodies and developed with SuperSignal West Pico Plus chemiluminescent substrate (ThermoFisher #34580) and imaged using an Amersham Imager 600.

### Human total RNA Isolation and quantitative PCR

The harvested cells were washed and incubated with 1 ml of TRIzol reagent and incubated for 5 minutes. 200 μl of chloroform was added per sample and vortexed. Samples were centrifuged, and clear supernatant was harvested in a new Eppendorf tube containing 318 μl of 100% ethanol. Samples were transferred to the RNeasy column (Qiagen #74106) and centrifuged. The columns were washed with 500 μl of RW1 buffer and treated with DNase (Qiagen #79254) for 30 minutes at room temperature. The columns were then washed with 500 μl of RW1 buffer, followed by 500 μl of RPE buffer and eluted with 30 μl of RNase-free water. Isolated RNA was reversely transcribed using High-Capacity cDNA Reverse Transcription Kit (ThermoFisher #4368813). cDNA was diluted 1:10 in H_2_O and was used for quantitative PCR using Power SYBR Green PCR Master Mix (ThermoFisher #4368708) following manufacturer protocol and run on Applied Biosystem QuantStudio Real-Time PCR System using targeted primer and reference primer sets (See **Key Resource** Supplementary Table S5).

### Reporting summary

Further information on research design is available in the Nature Portfolio Reporting Summary linked to this article.

## Supplementary information

Supplementary information

Reporting Summary

Transparent Peer Review file

## Source data

Source data

## Data Availability

The cryo-EM maps and atomic coordinates for *E.coli* produced recombinant *Ct*NAT10/cytidine-acetone-CoA, *E.coli* produced recombinant *Ct*NAT10/cytidine-amide-CoA/ADP and *Sf9* produced recombinant *Ct*NAT10/cytidine-amide-CoA/ADP have been deposited to the Electron Microscopy Data Bank (EMDB) (accession codes: 43996, 44038 and 44042, respectively) and the Protein Data Bank (PDB) (accession codes: 9AYM, 9B0E and 9B0I, respectively). Other primary data files have been provided with the manuscript. Source data are provided in this paper.
