# Supplementary material for: Structure of the NAT10 acetyltransferase and mechanism of tRNA acetylation
Source: Nat Commun. 2026 Jun 20;17:7763. doi: 10.1038/s41467-026-74222-6 (PMC13433802; doi:10.1038/s41467-026-74222-6)
Supplement: Supplementary file 1 [file 41467_2026_74222_MOESM1_ESM.pdf]

SUPPLEMENTAL FILE

Structure of the NAT10 Acetyltransferase and Mechanism of tRNA Acetylation

Supplemental Figures (with Figure Legends) 1-12

Supplemental Tables S1-S5

Supplemental References 1-2

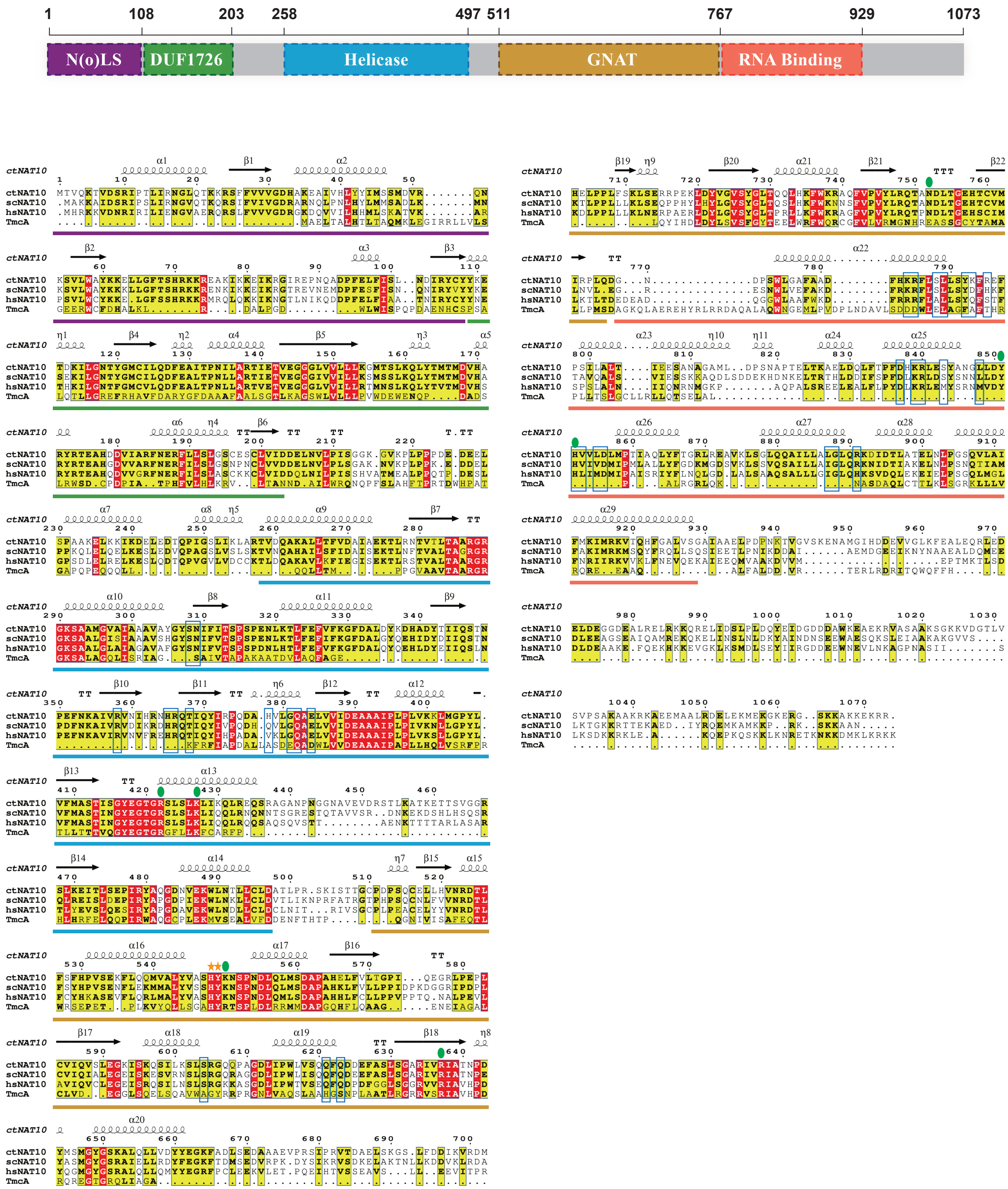

1
108
203
258
497
511
767
929
1073
N(o)LS
DUF1726
Helicase
GNAT
RNA Binding
ctNAT10
scNAT10
hsNAT10
TmcA

Supplemental Figure 1. Sequence alignment of NAT10 homologs. The aligned species are *Chaetomium thermophilum* (ct), *Saccharomyces cerevisiae* (sc), *Homo sapiens* (hs) and *Escherichia coli* (TmcA). Secondary structure elements are indicated for the *E.coli* produced recombinant *Ct*NAT10/cytidine-amide-CoA/ADP cryo-EM structure. Conserved amino acids are in white in red-filled rectangles. Similar residues are in bold in yellow-filled rectangles, surrounded by black lines. Residues that are involved in *Ct*NAT10 dimerization surface are boxed in blue rectangles. Domain boundaries are indicated below the sequence alignment, with colors corresponding to the scaled schematic representation of *Ct*NAT10 domain organization shown above. Catalytic residues are indicated with yellow stars (★) and putative RNA binding residues are marked with green ovals (○) above the alignment. Alignment was performed using ESPript 3 [1].

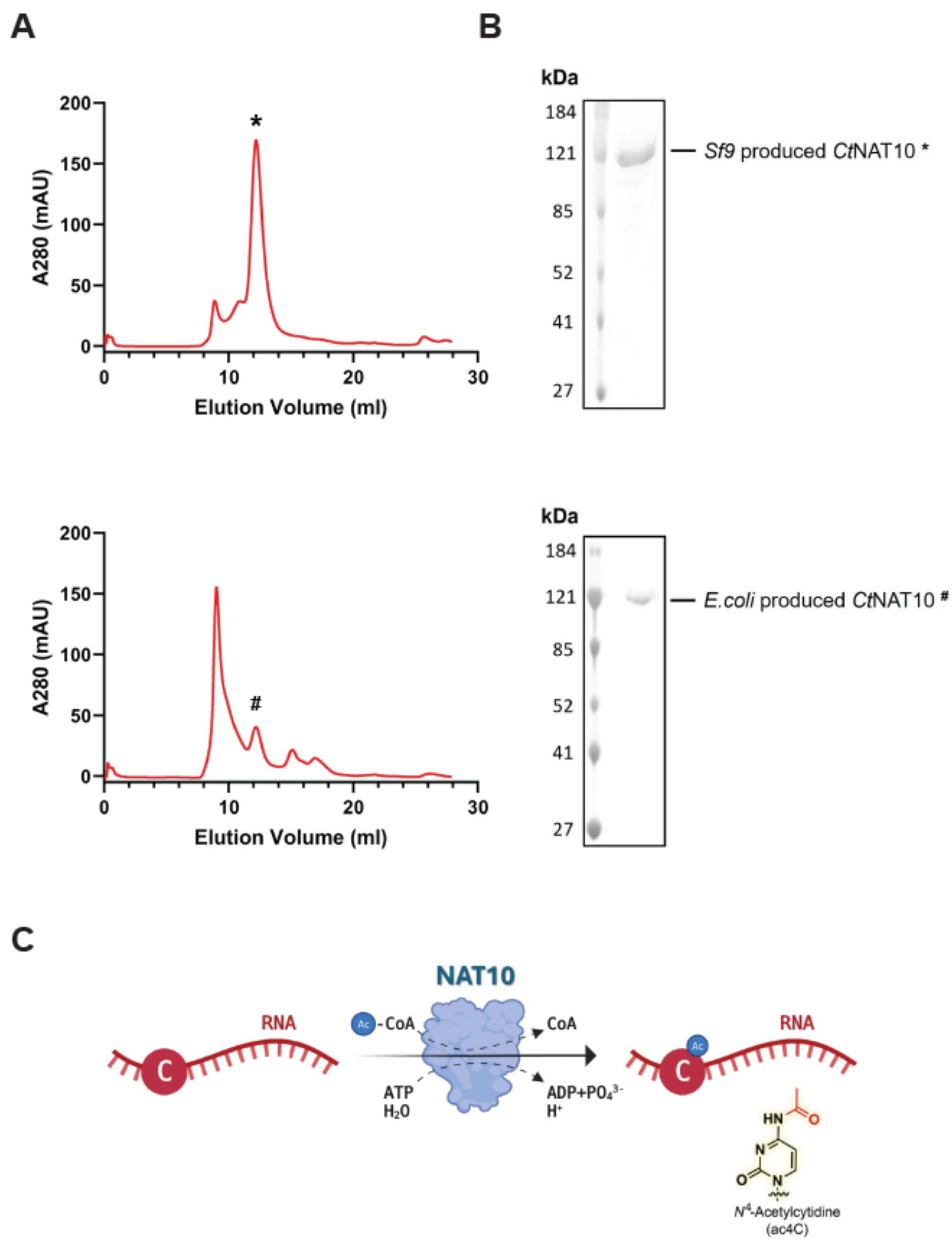

Supplemental Figure 2. Production and purification of recombinant *Ct*NAT10 from *Sf9* and *E.Coli*. (A) Superdex-200 size-exclusion chromatogram of dimeric N-terminally His-tagged *Ct*NAT10 purified from *Sf9* (*) and *E.Coli* (#). **(B)** SDS-PAGE (12.5% acrylamide) of intact, homogeneous *Ct*NAT10 protein produced in *Sf9* (*) and *E.Coli* (#). Polypeptides were visualized by staining with Coomassie Blue dye. The positions and sizes (in kDa) of marker proteins are indicated on the left. **(C)** Cartoon schematic of the NAT10-catalyzed RNA acetylation reaction. Created in BioRender. Marmorstein, R. (2026) (https://BioRender.com/9aow4sr)

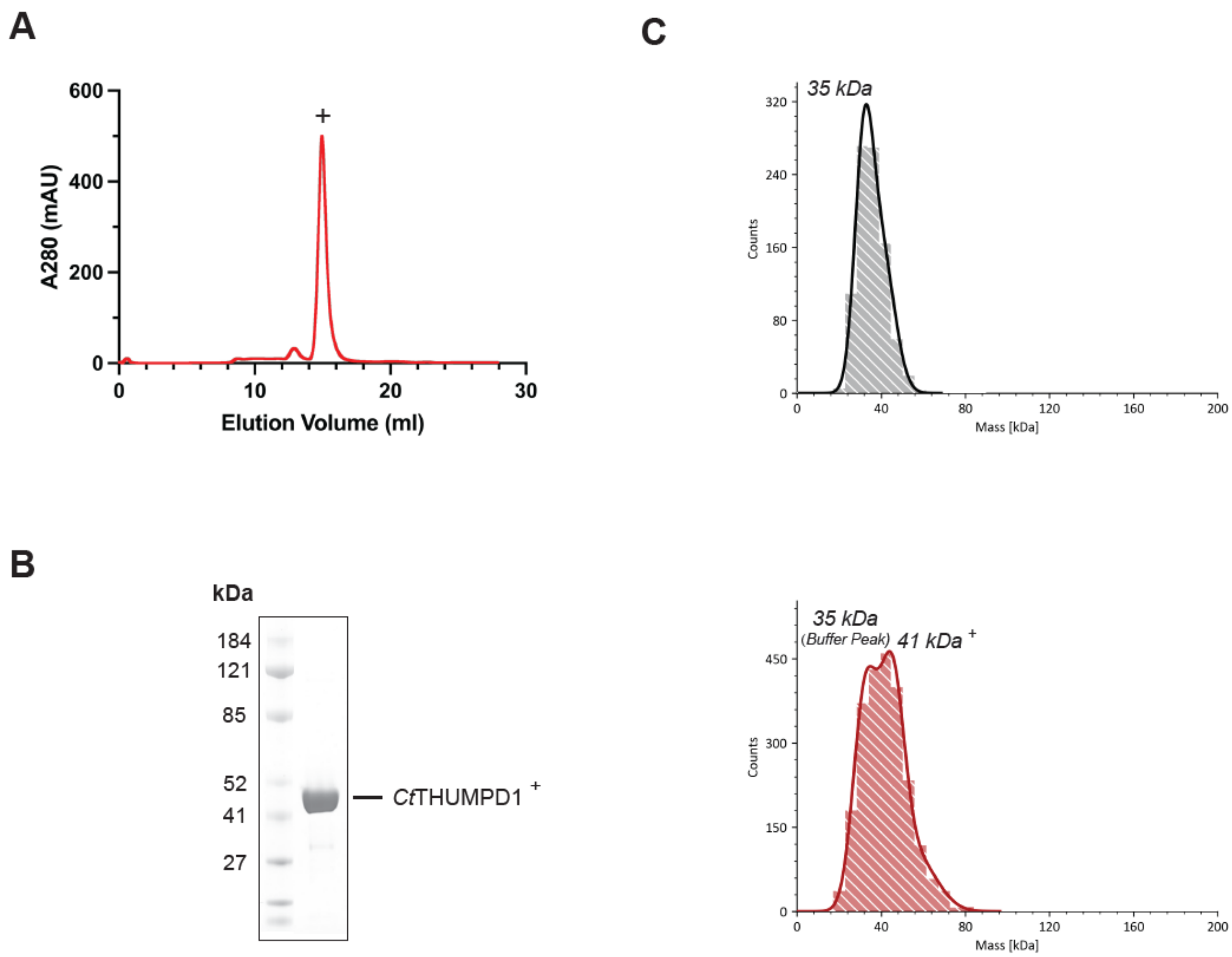

Supplemental Figure 3. *Ct*THUMPD1 production, purification and characterization. (A) Superdex-200 size-exclusion chromatogram of monomeric recombinant *Ct*THUMPD1 purified from *E.Coli* ($^{+}$). **(B)** SDS-PAGE (12.5% acrylamide) of intact, homogeneous *Ct*THUMPD1 protein produced in *E.Coli* ($^{+}$). Polypeptides were visualized by staining with Coomassie Blue dye. The positions and sizes (in kDa) of marker proteins are indicated on the left. **(C)** Mass photometry characterization of recombinant *Ct*THUMPD1. Histograms in panels show the particle counts of buffer (top) *Ct*THUMPD1 (bottom) at the indicated molecular mass. Solid lines are Gaussian fits to the peaks and the mass of the Gaussian fit are indicated above each peak. *Ct*THUMPD1 was mostly present as a monomer (theoretical MW of *Ct*THUMPD1 monomer: 40.2 kDa), and the monomer peak for *Ct*THUMPD1 sample is indicated in the bottom panel ($^{+}$).

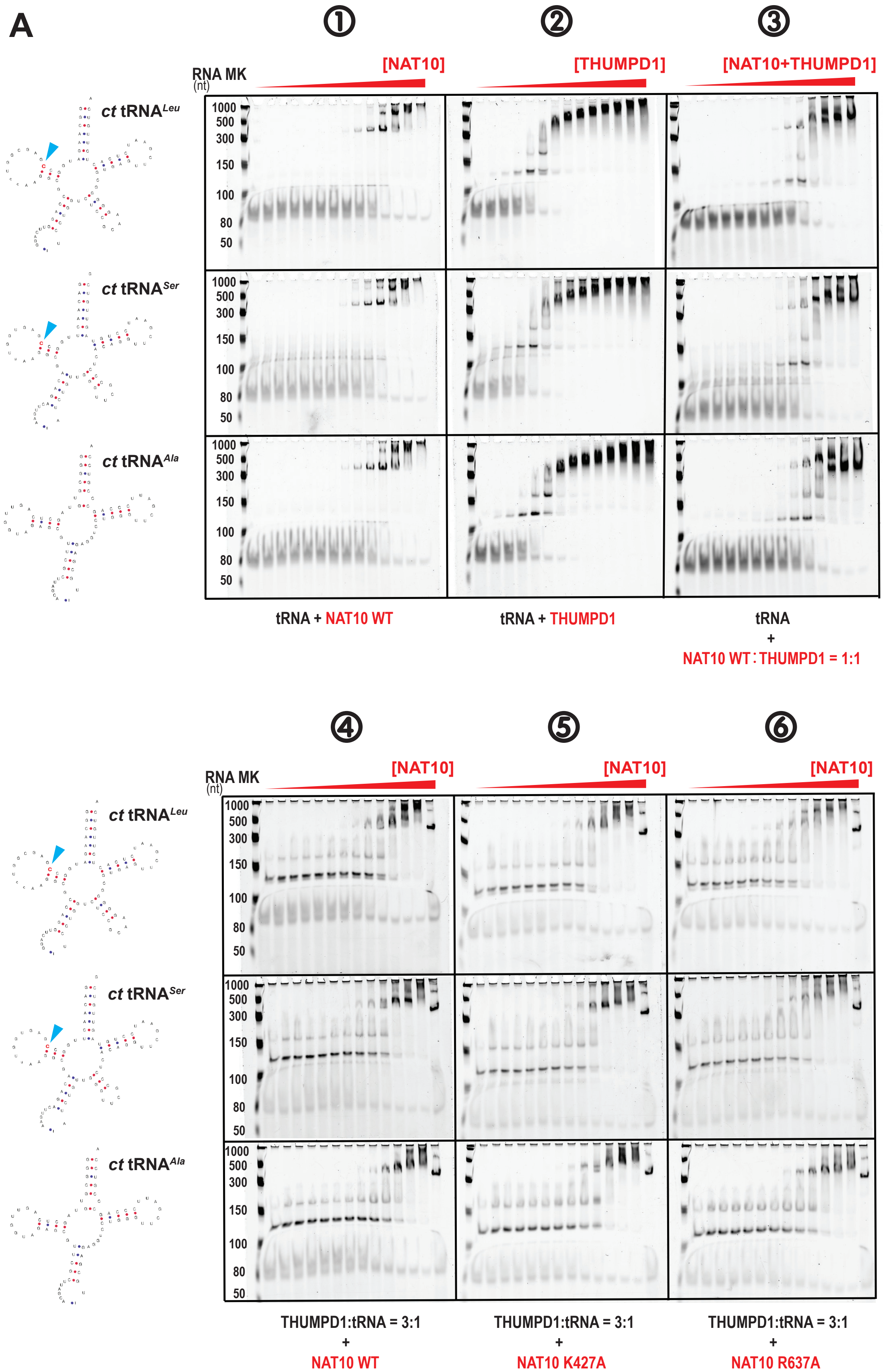

A
①
②
③
RNA MK
(nt)
[NAT10]
[THUMPD1]
[NAT10+THUMPD1]
ct tRNA^Leu
ct tRNA^Ser
ct tRNA^Ala
1000
500
300
150
100
80
50
tRNA + NAT10 WT
tRNA + THUMPD1
tRNA
+
NAT10 WT : THUMPD1 = 1:1
④
⑤
⑥
RNA MK
(nt)
[NAT10]
[NAT10]
[NAT10]
THUMPD1:tRNA = 3:1
+
NAT10 WT
THUMPD1:tRNA = 3:1
+
NAT10 K427A
THUMPD1:tRNA = 3:1
+
NAT10 R637A

B

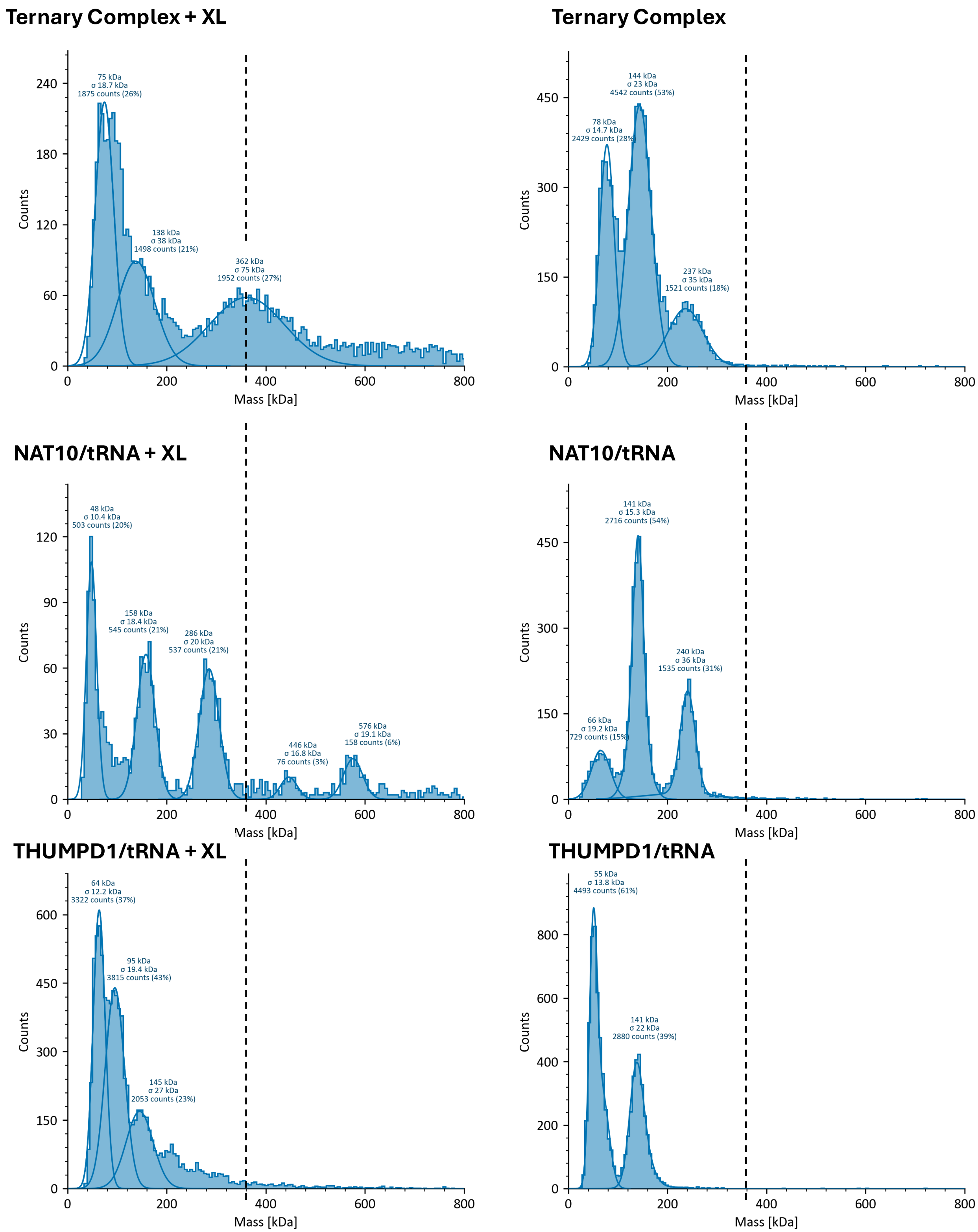
Ternary Complex + XL
75 kDa
σ 18.7 kDa
1875 counts (26%)
138 kDa
σ 38 kDa
1498 counts (21%)
362 kDa
σ 75 kDa
1952 counts (27%)
Counts
Mass [kDa]
Ternary Complex
144 kDa
σ 23 kDa
4542 counts (53%)
78 kDa
σ 14.7 kDa
2429 counts (28%)
237 kDa
σ 35 kDa
1521 counts (18%)
Counts
Mass [kDa]
NAT10/tRNA + XL
48 kDa
σ 10.4 kDa
503 counts (20%)
158 kDa
σ 18.4 kDa
545 counts (21%)
286 kDa
σ 20 kDa
537 counts (21%)
446 kDa
σ 16.8 kDa
76 counts (3%)
576 kDa
σ 19.1 kDa
158 counts (6%)
Counts
Mass [kDa]
NAT10/tRNA
141 kDa
σ 15.3 kDa
2716 counts (54%)
240 kDa
σ 36 kDa
1535 counts (31%)
66 kDa
σ 19.2 kDa
729 counts (15%)
Counts
Mass [kDa]
THUMPD1/tRNA + XL
64 kDa
σ 12.2 kDa
3322 counts (37%)
95 kDa
σ 19.4 kDa
3815 counts (43%)
145 kDa
σ 27 kDa
2053 counts (23%)
Counts
Mass [kDa]
THUMPD1/tRNA
55 kDa
σ 13.8 kDa
4493 counts (61%)
141 kDa
σ 22 kDa
2880 counts (39%)
Counts
Mass [kDa]

C

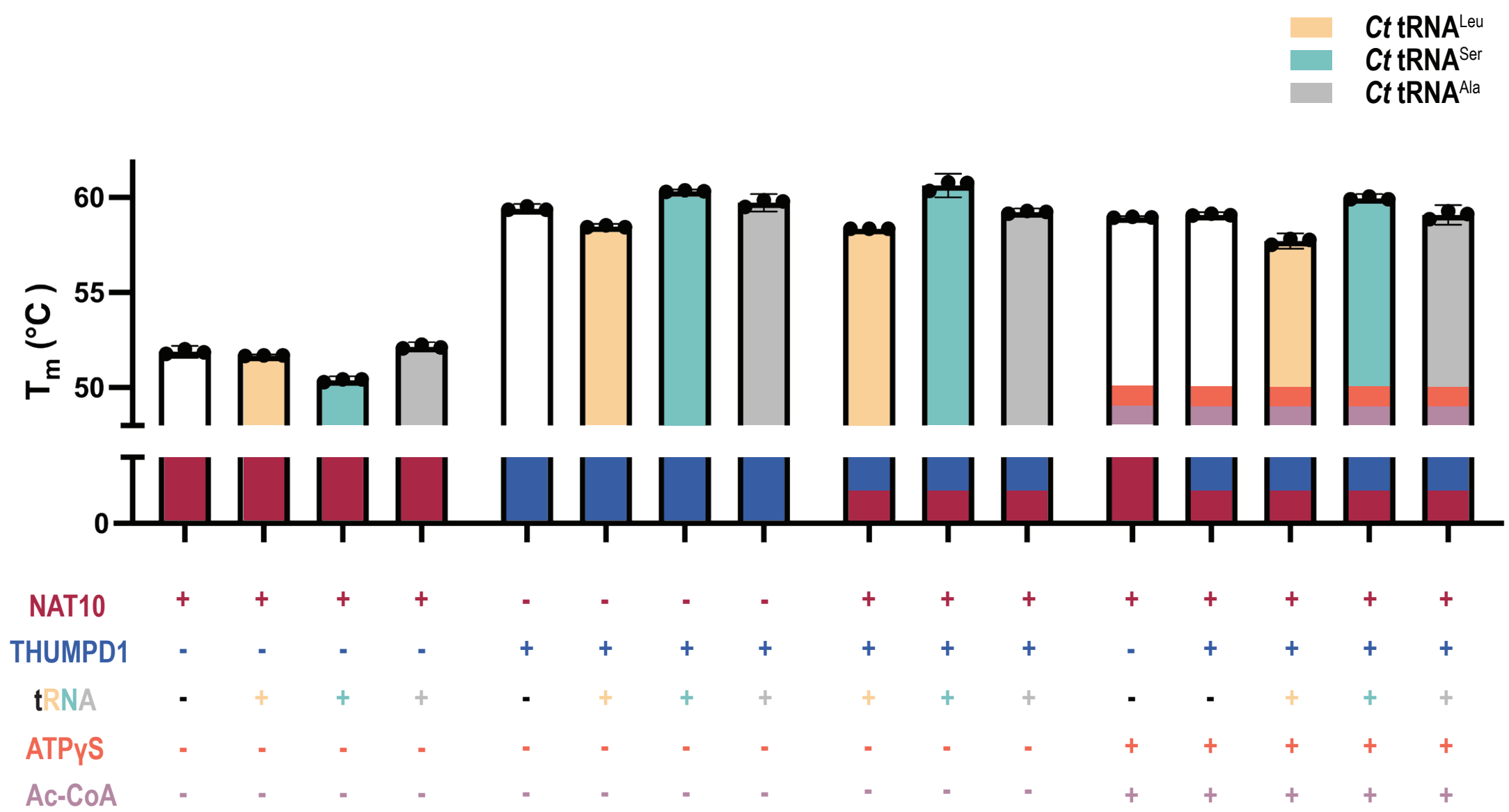

Supplemental Figure 4. Electrophoretic mobility shift assay (EMSA) of purified *Ct*NAT10 WT/alanine-mutants with cognate and non-cognate tRNA substrates, and thermal stability of *Ct*NAT10 in the presence of tRNA and THUMPD1. (A) EMSA was carried out with 6% polyacrylamide native gels and stained with SYBR™ Gold to visualize shifted tRNA substrates. **(Column ①)** tRNA input was fixed at 0.25 μM and *Ct*NAT10 WT was titrated at 0, 1.75, 3.5, 7, 14, 27, 55, 109, 219, 436, 875, 1750, 3500 and 7000 nM (from left to right). **(Column ②)** tRNA input was fixed at 0.25 μM and *Ct*THUMPD1 was titrated at 0, 42, 84, 168, 337, 673, 1347, 2695, 5391, 10780, 21563, 43125, 86250 and 172500 nM (from left to right). **(Column ③)** *Ct*NAT10 WT and *Ct*THUMPD1 proteins were pre-assembled in a 1:1 molar ratio, and incubated at 4 °C for 2 h. tRNA input was fixed at 0.25 μM and *Ct*NAT10 + *Ct*THUMPD1 complex was titrated at 0, 1.75, 3.5, 7, 14, 27, 55, 109, 219, 436, 875, 1750, 3500 and 7000 nM (from left to right). **(Column ④ - ⑥)** 0.25 μM of tRNA were pre-incubated with 0.75 μM of *Ct*THUMPD1 on ice for 2 h, then *Ct*NAT10 WT/alanine-mutants were titrated at 0, 1.75, 3.5, 7, 14, 27, 55, 109, 219, 436, 875, 1750, 3500 and 7000 nM (from left to right) into tRNA/*Ct*THUMPD1 complex. **(B)** Mass Photometry of Nat10 complexes under the highest protein titration conditions used in 4A. Samples were analyzed at concentrations diluted to 10 nM (right) or crosslinked with glutaraldehyde to trap complexes prior to dilution (left). The dotted line represents the calculated molecular weight for a NAT10/THUMPD1/tRNA complex of stoichiometry of 2:2:2. **(C)** Nano Differential Scanning Fluorimetry of *Ct*NAT10 (red) melting temperature in the absence and presence of cofactor *Ct*THUMPD1 (blue), Ac-CoA (purple), ATPγS (orange) and *Ct*

tRNA (Leu: yellow, Ser: cyan, Ala: grey). Each ligand was evaluated at concentrations sufficient to saturate *Ct*NAT10, as corroborated by the DSF results (**Figure 1C**) and EMSA results. Averaged values of three technical replicates with S.D. values are shown. Source data are provided as a **Source Data** file.

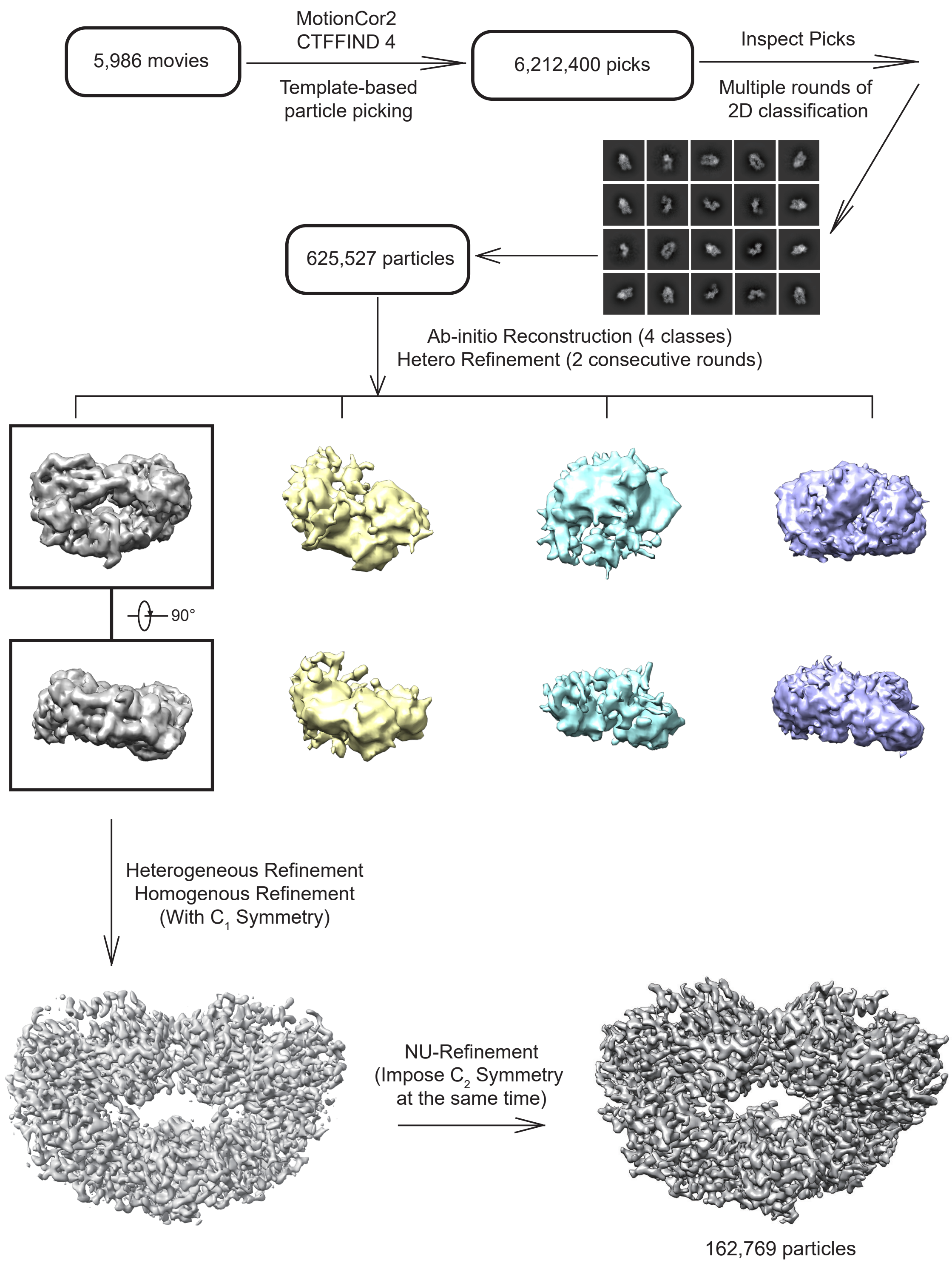

Supplemental Figure 5. Cryo-EM image processing workflow of *Ct*NAT10 structures. Workflow, 2D and 3D classification scheme for *Ct*NAT10$_{Sf9}$ /cytidine-amide-CoA/ADP is shown. The workflows for the other two structures are similar.

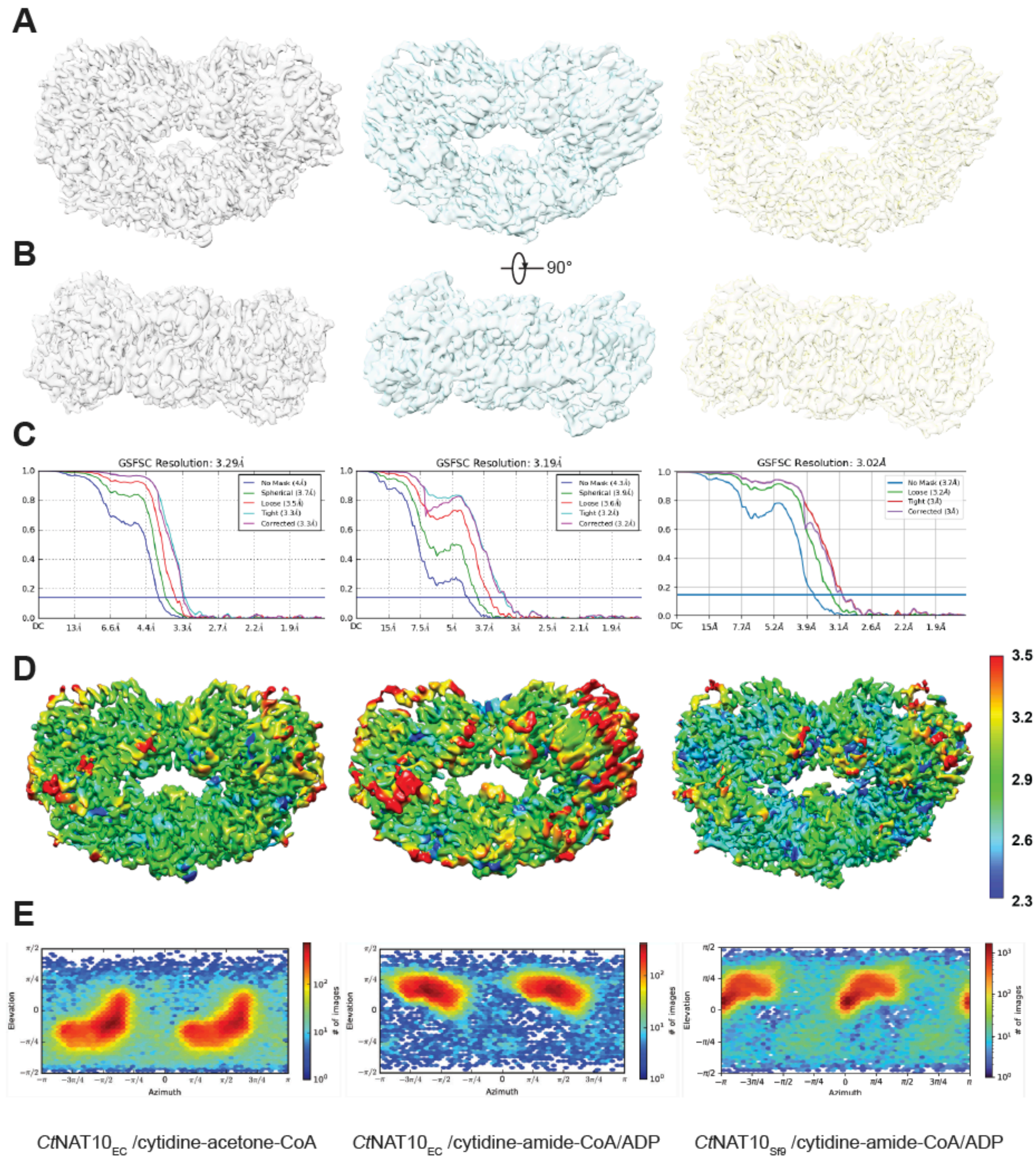

Supplemental Figure 6. Cryo-EM maps quality control and resolution estimation. **(A)** Front and **(B)** bottom view, **(C)** Fourier shell correlation (FSC) curves, **(D)** local resolution estimation and **(E)** angular distribution for the cryo-EM density of *E.coli* produced recombinant *Ct*NAT10/cytidine-acetone-CoA, *E.coli* produced recombinant *Ct*NAT10/cytidine-amide-CoA/ADP and *Sf9* produced recombinant *Ct*NAT10/cytidine-amide-CoA/ADP (from left to right), respectively. The volume threshold levels for displaying the three Cryo-EM maps are 0.264, 0.229 and 0.394, respectively. All FSC curve plots are generated by cryoSPARC.

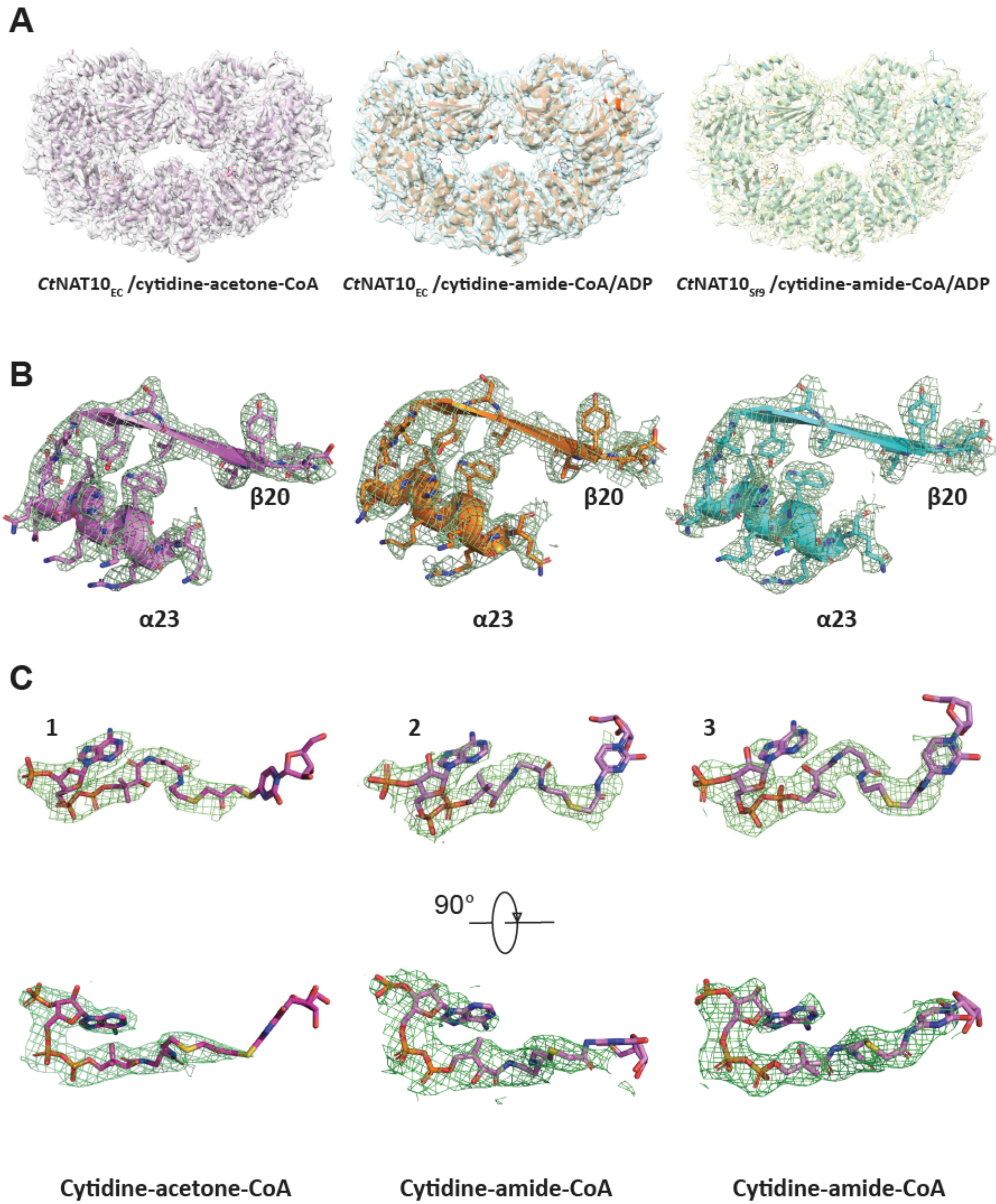
A
CtNAT10EC /cytidine-acetone-CoA
CtNAT10EC /cytidine-amide-CoA/ADP
CtNAT10Sf9 /cytidine-amide-CoA/ADP
B
β20
α23
β20
α23
β20
α23
C
1
2
3
90°
Cytidine-acetone-CoA
Cytidine-amide-CoA
Cytidine-amide-CoA

Supplemental Figure 7. Atomic models fitted into the cryo-EM maps. (A) Atomic models of *E.coli* produced recombinant *Ct*NAT10/cytidine-acetone-CoA (lavender), *E.coli* produced recombinant *Ct*NAT10/cytidine-amide-CoA/ADP (orange) and *Sf9* produced recombinant *Ct*NAT10/cytidine-amide-CoA/ADP (cyan) cryo-EM structures, were fitted into the corresponding cryo-EM maps using Chimera, respectively. **(B)** The "fit-in-map" of the catalytically important secondary structure elements β20-α23 (residue 720-741) for all three *Ct*NAT10 structures are shown, for demonstrating the cryo-EM map quality in detail. A contour level of 8 σ was applied to all the maps. **(C) and (D)** Cryo-EM density of ligands. Density of cytidine-CoA conjugates (C - 1, 2, 3) and ADP-(Mg^{2+}) (D - 1, 2), for three *Ct*NAT10 structures (the ligands are in the same column with the *Ct*NAT10 structure that they are bound to). A contour level of 7 σ is shown in C-1, a contour level of 10 σ is shown in D-1 and a contour level of 8 σ is shown in C-2, C-3 and D-2. All mesh of cryo-EM maps and corresponding fit-in model figures are depicted using Pymol. **(E)** Cytidine-amide-CoA and ADP-Mg^{2+} are shown fitted into the electron density map and represented as stick models. *Sf9* produced recombinant *Ct*NAT10 residues forming the binding site are displayed simultaneously with the ligands at the same contour level of 8 σ, illustrating the quality of the map fit and the interactions within the active site.

A

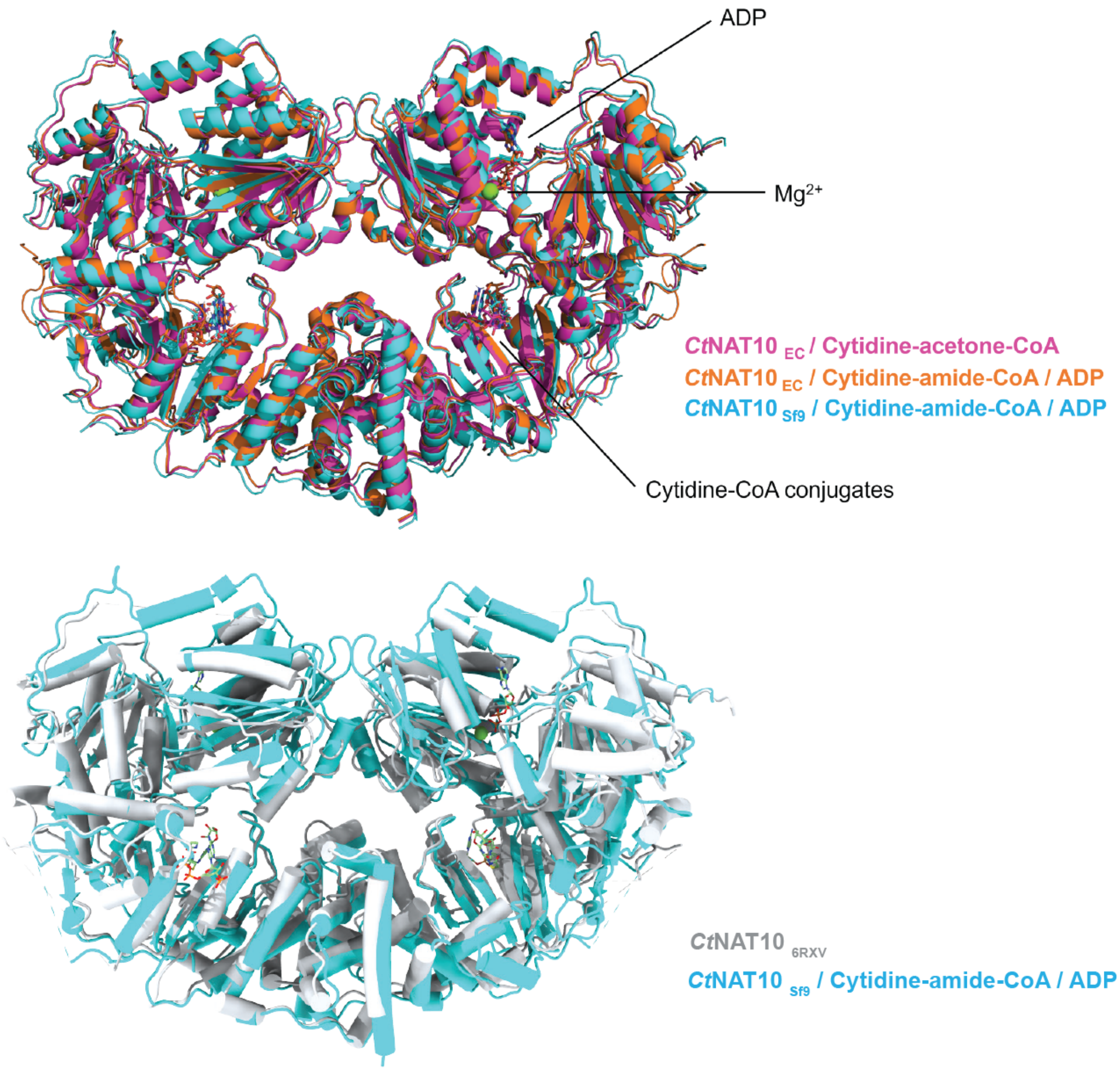

B

	RMSD when aligned to *Ct*NAT10$_{EC}$/cytidine-acetone-CoA	RMSD when aligned to *Ct*NAT10$_{EC}$ /cytidine-amide-CoA/	RMSD when aligned to *Ct*NAT10$_{Sf9}$ /cytidine-amide-CoA /ADP	RMSD when aligned to *Ct*NAT10 from 6RXV
*ct*NAT10$_{EC}$/cytidine -acetone-CoA	-	0.506	1.302	-
*ct*NAT10$_{EC}$ /cytidine-amide-CoA/ADP	0.506	-	1.151	-
*ct*NAT10$_{Sf9}$ /cytidine-amide-CoA /ADP	1.302	1.151	-	-
*Ct*NAT10 from 6RXV	-	-	-	1.185

Supplemental Figure 8. Comparison of *Ct*NAT10 cryo-EM structures. (A) Superimposition of the *E.coli* produced recombinant *Ct*NAT10/cytidine-acetone-CoA (magenta), *E.coli* produced recombinant *Ct*NAT10/cytidine-amide-CoA/ADP (orange), *Sf9* produced recombinant *Ct*NAT10/cytidine-amide-CoA/ADP (cyan) cryo-EM structures, and the ribosome-bound *Ct*NAT10 structure (PDB: 6RXV, grey). **(B)** Root-mean-squared deviations (RMSD, in Å) between the three recombinant *Ct*NAT10 cryo-EM structures and the ribosome-bound *Ct*NAT10 structure (PDB: 6RXV), calculated from Cα atom superimpositions.

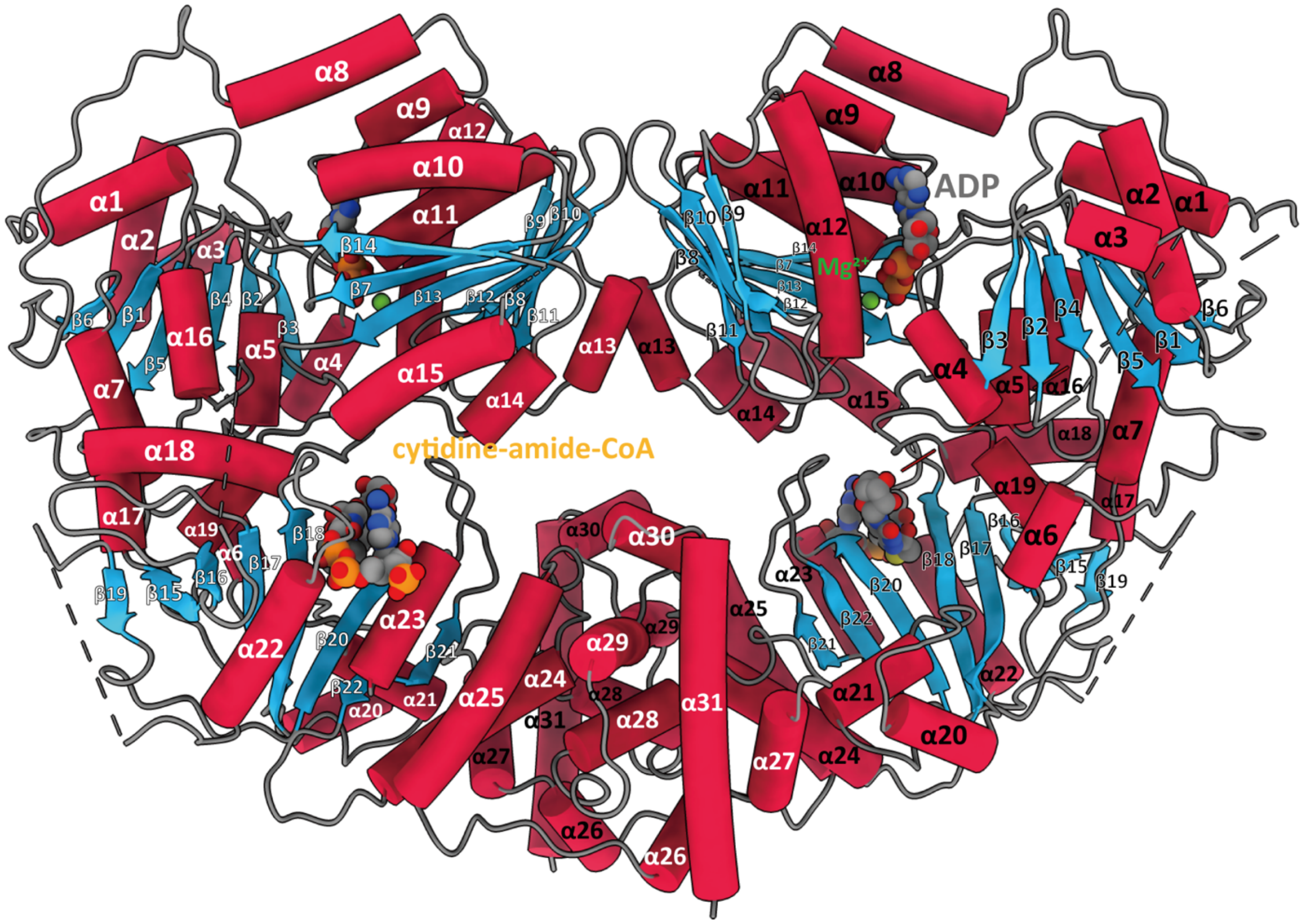

Supplemental Figure 9. Cartoon representation of *Ct*NAT10 structure with labelled secondary structures. Since the three *Ct*NAT10 structures reported in this paper superimposed well, here we use *Sf9* produced recombinant *Ct*NAT10/cytidine-amide-CoA/ADP structure as the representative. α-helix (crimson) and β-sheet (skyblue) are labelled and numbered in the order from N-terminus to C-terminus. One of the subunits of the two-fold symmetric *Ct*NAT10 dimer is labeled with black, whereas the other is labeled with white to facilitate visualization of the dimeric assembly.

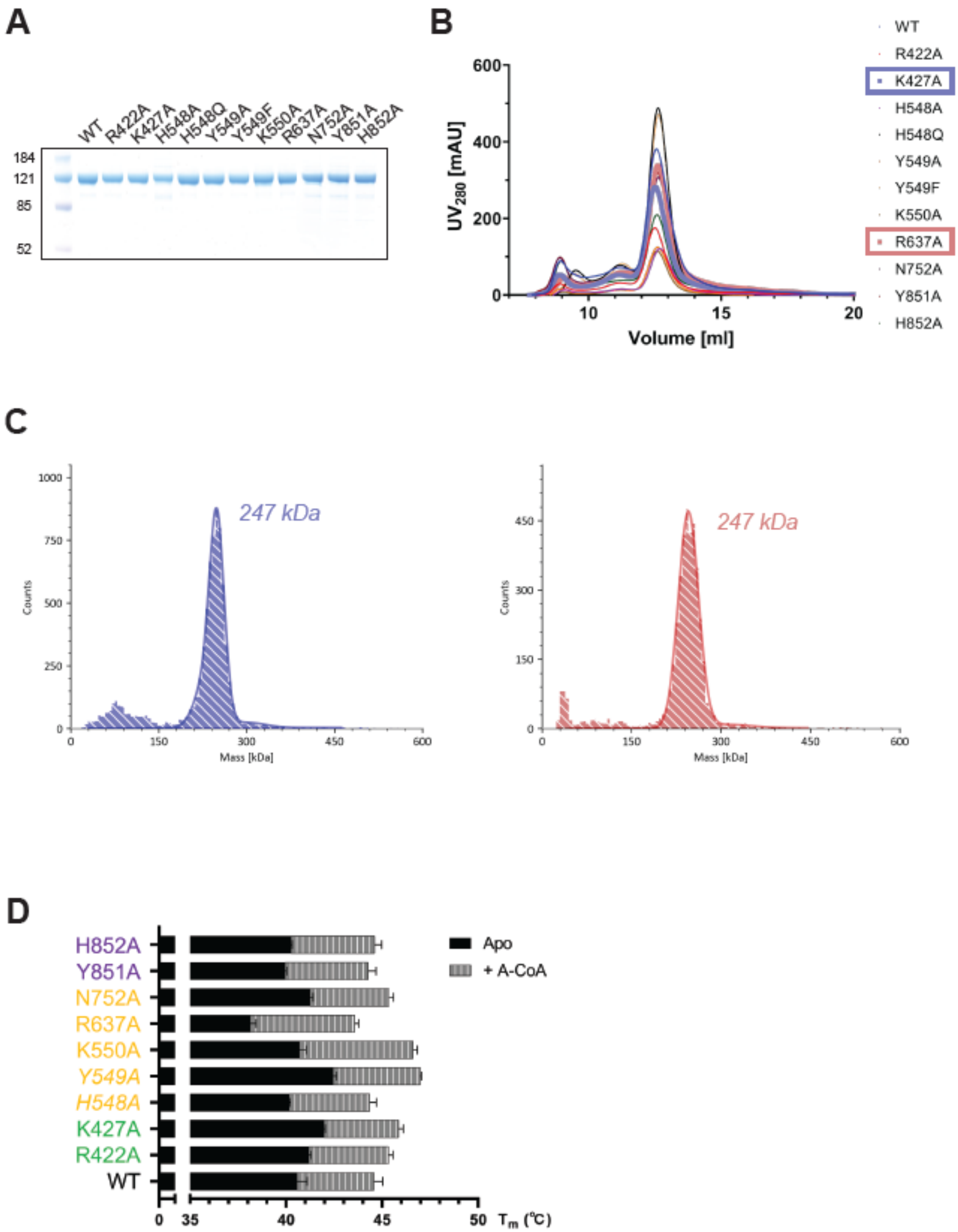
A
WT R422A K427A H548A H548Q Y549A Y549F K550A R637A N752A Y851A H852A
184
121
85
52
B
WT
R422A
K427A
H548A
H548Q
Y549A
Y549F
K550A
R637A
N752A
Y851A
H852A
UV280 [mAU]
Volume [ml]
C
247 kDa
Counts
Mass [kDa]
247 kDa
Counts
Mass [kDa]
D
H852A
Y851A
N752A
R637A
K550A
Y549A
H548A
K427A
R422A
WT
Apo
+ A-CoA
Tm (°C)

Supplemental Figure 10. Purification, integrity and thermal stability assessment of recombinant *Ct*NAT10 single-alanine mutant proteins. (A) SDS-PAGE (12.5% acrylamide) of intact, homogeneous *Ct*NAT10 mutant proteins produced in *Sf9*. Polypeptides were visualized by staining with Coomassie Blue dye. The positions and sizes (in kDa) of marker proteins are indicated on the left. **(B)** Superdex-200 size-exclusion chromatogram of dimeric N-terminally His-tagged *Ct*NAT10 single mutants purified from Sf9. **(C)** Mass photometry characterization of representative recombinant *Ct*NAT10 mutants. Histograms in panels show the particle counts of *Ct*K427A (left panel, blue) *Ct*R637A (right panel, salmon) at the indicated molecular mass. Solid lines are Gaussian fits to the peaks, and the mass of the Gaussian fit are indicated above each peak. **(D)** DSF evaluation of *Ct*NAT10 mutant constructs in the absence (solid black bar) and presence (patterned bar) of acetyl-CoA (A-CoA). The mutant names are color-coded according to the regions in **Figure 4D**. Averaged values of four technical replicates with S.D. values are shown for each dataset. Source data are provided as a **Source Data** file.

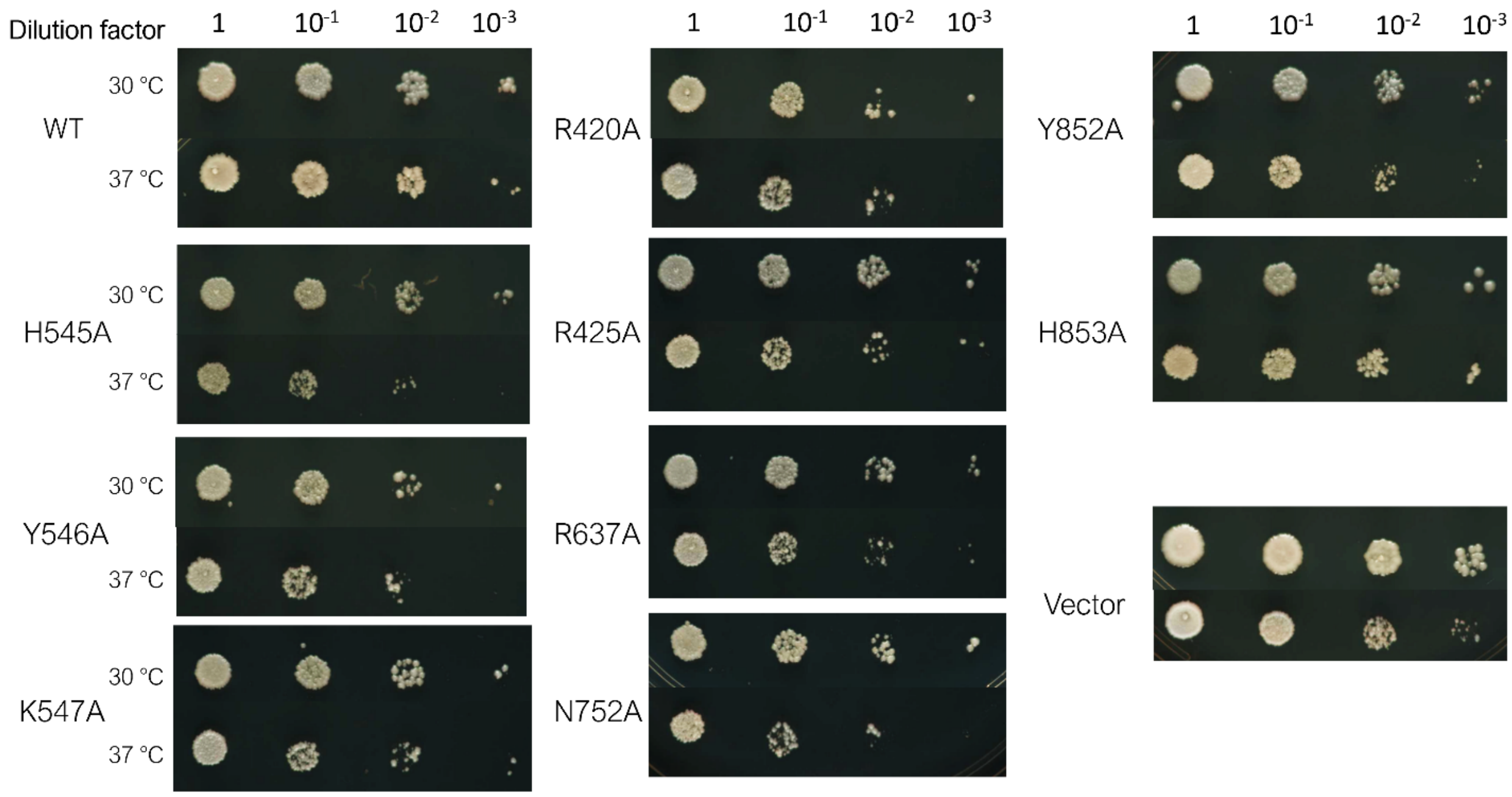

Supplemental Figure 11. Growth assay data for Figure 5. Strains were grown, diluted, plated on YPD plates, and grown at the indicated temperatures for 48 h. The mutant names are color-coded according to the regions in **Figure 4D**.

A

sample ID	Kre33 plasmid	Heat-dependent growth phenotype	% misincorporation at 18S rRNA h45	% misincorporation at tRNA^{ser} AGA
X01	WT	No	28	27
X02	H545A	Yes	1	0
X04	R420A	Yes	nd	0
X06	R637A	Yes	3	0
X09	Y852A	No	nd	28
X14	empty vector	Yes	0	0

B

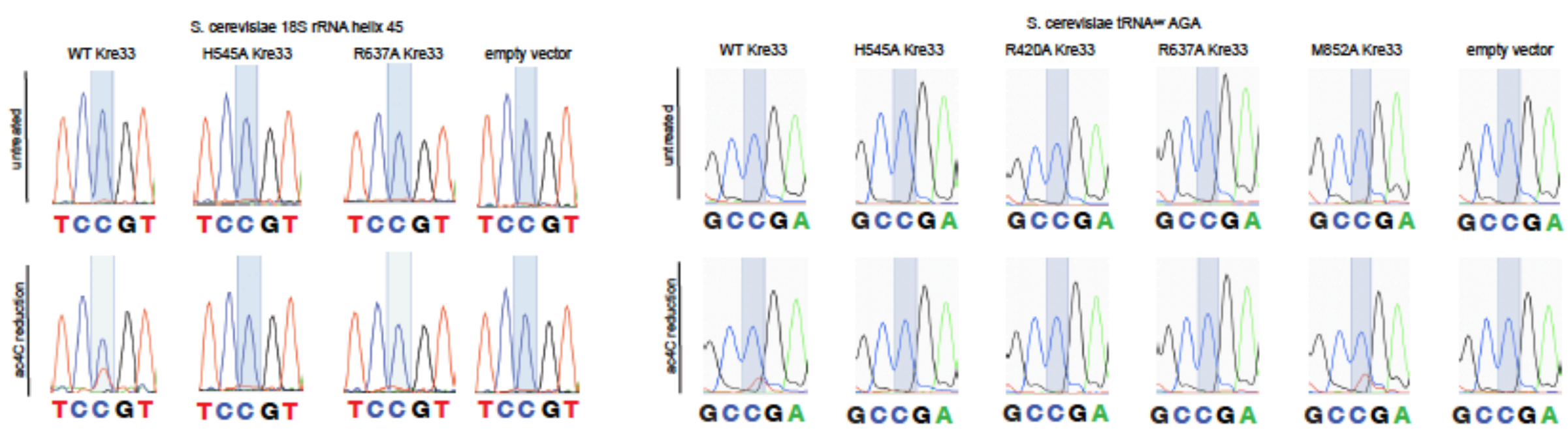

C

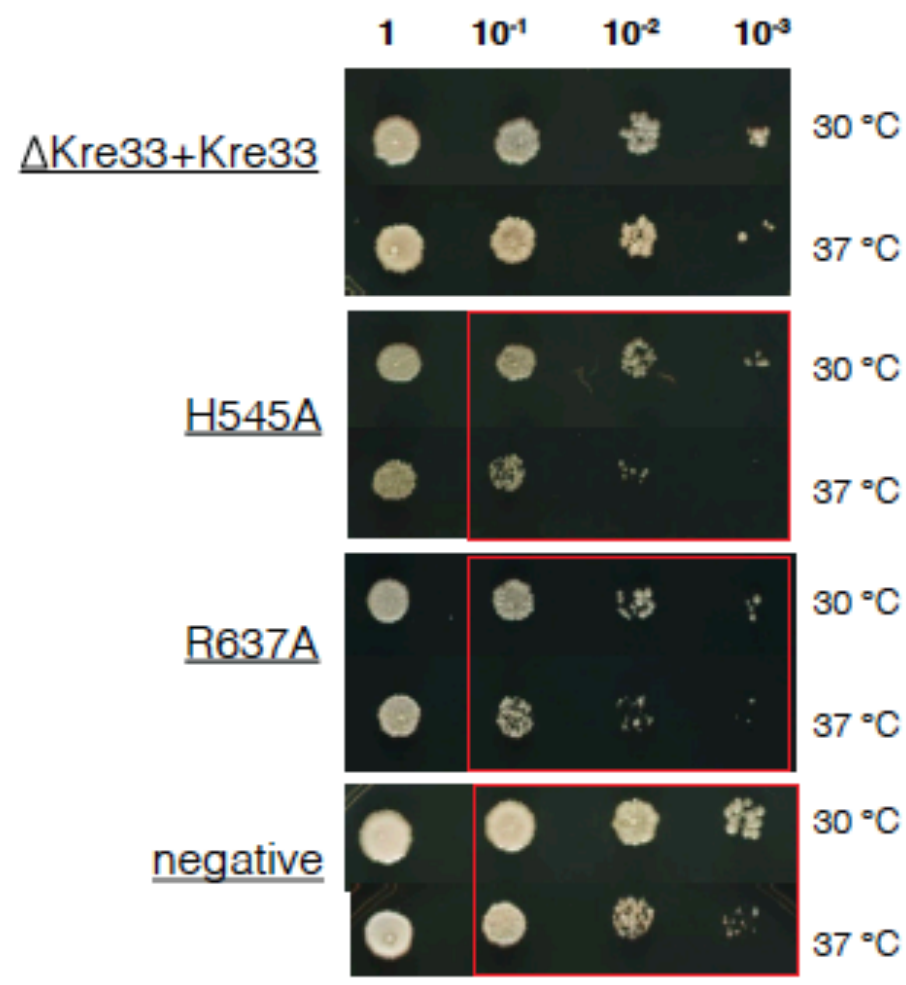

Supplementary Figure 12. Rescue of high-temperature growth defect correlates with rescue of RNA cytidine acetylation. (A) Strains were transformed as described in Figure 5A and total RNA was isolated by hot acidic phenol method. The presence of ac4C at 18S SSU helix 45 and tRNA^{ser} AGA were then assessed using a quantitative ac4C sequencing reaction described in methodology section[2]. ScKre33 mutants that successfully rescue exhibit no heat-dependent growth phenotype and also display rescue of acetylation. (B) Exemplary ac4C sequencing data. (C) ScKre33 mutants that do not rescue exhibit a reduced growth at higher temperatures and do not display rescue of helix 45 or tRNA^{ser} acetylation (H545A, R637A, empty vector) are marked with red boxes. nd = not determined.

Key Resource Tables

Table S1, RNA for EMSA and *In vitro* RNA acetylation assay.

RNA probe name	*Sequence	Manufacturer
	RNA fragments used in Figure 1B and 1C	
ct-$\mathrm{tRNA}^{Leu}_{CAG}$	GGCAAGAUGGCCGAGCGGUCCAAGGCGCCAGGUUCAGGUCCUGGUCUC CGCAAGGGGGCGUGAGUUCGAAUCUCACUCUUGUCA [2,3]	GeneScript
ct-$\mathrm{tRNA}^{Ser}_{TGA}$	GACAACUUGGCCGAGUGGUUAAGGCGACAGACUUGAAAUCUGUUGGGU UCGCCCUCACAGGUUCGAAUCCUGUAGUUGUCG [2,3]	GeneScript
sc-rRNA^{h45}	GAGGAACUAAAAGUCGUAACAAGGUUUCCGUAGGUGAACCUGCGGAAG GAUCAUUA [4]	GeneScript
ct-rRNA^{h45}	GAGGAAGUAAAAGUCGUAACAAGGUCUCCGUAGGUGAACCUGCGGAGG GAUCAU [5]	GeneScript
	RNA fragments used in Figure 1A, 4E and Figure S4	
Ct$\mathrm{tRNA}^{\mathrm{Leu}}$	GGCAAGAUGGCCGAGCGGUCCAAGGCGCCAGGUUCAGGUCCUGGUCUC CGCAAGGGGGCGUGAGUUCGAAUCUCACUCUUGUCA [2,3]	In house IVT
Ct$\mathrm{tRNA}^{\mathrm{Ser}}$	GACAACUUGGCCGAGUGGUUAAGGCGACAGACUUGAAAUCUGUUGGGU UCGCCCUCACAGGUUCGAAUCCUGUAGUUGUCG [2,3]	In house IVT
Ct$\mathrm{tRNA}^{\mathrm{Ala}}$	GGGCGUGUAGCUCAGUUGGUAGAGCGUUCGCUUAGCAUGCGAGAGGUC CUGGGUUCGAUUCCCAGCCCGUCCA [2,3]	In house IVT

*The reference of each RNA sequence is annotated.

Table S2. Yeast strains used in this study

Strain Name	Genotype	Description	Source
Y22944	BY4743 Mat a/α his3Δ1/his3Δ1 leu2Δ0/leu2Δ0 lys2Δ0/LYS2 MET15/met15Δ0 ura3Δ0/ura3Δ0 YNL132w::kanMX4/YNL132w	Heterozygous KRE33 diploid strain, not used in this study	Euroscarf
Δkre33 + pSH35-C	Haploid derivative of Y22944: Mat a his3Δ1 leu2Δ0 met15Δ0 ura3Δ0 YNL132w::kanMX4 [pSH35-C]	Strain with endogenous KRE33 deleted, maintained with plasmid expressing catalytically deficient Kre33-H545A (GCA codon)	Sharma et al., 2015

Table S3. Expression plasmids used in yeast for this study

Plasmid Name	Description	Selection Marker	Source
HQHQHH-Kre33 NM_01182970.1/pD1237	Wild-type ScKre33 expressed from TDH3 promoter and CYC1 terminator	HIS3	This study
HQHQHH-Kre33 H545A/pD1237	Catalytic mutant (GCT codon)	HIS3	This study
HQHQHH-Kre33 Y546A/pD1237	Active site mutant	HIS3	This study
HQHQHH-Kre33 K547A/pD1237	Basic patch 1 mutant	HIS3	This study
HQHQHH-Kre33 R637A/pD1237	Basic patch 1 mutant	HIS3	This study
HQHQHH-Kre33 N752A/pD1237	Basic patch 1 mutant	HIS3	This study
HQHQHH-Kre33 R420A/pD1237	Basic patch 3 mutant	HIS3	This study
HQHQHH-Kre33 K425A/pD1237	Basic patch 3 mutant	HIS3	This study
HQHQHH-Kre33 Y852A/pD1237	Basic patch 2 mutant	HIS3	This study
HQHQHH-Kre33 H853A/pD1237	Basic patch 2 mutant	HIS3	This study
pD1237	Empty vector control	HIS3	This study

Table S4, primary antibody used in human cell assays.

Antibody	Application	Manufacturer	Catalog	Concentration
LaminB1	WB	Abcam	ab16048	1:1000 (WB)
MMP3	WB	Abcam	ab52915	1:1000 (WB)
GAPDH	WB	Cell Signaling	5174S	1:2000 (WB)
NAT10	WB	Santa Cruz	SC-271770	1:1000 (WB)

Table S5, primer used in human cell assays.

Target	5' Sequence	3' Sequence
p16	ATGGAGCCTTCGGCTGACT	GTAACTATTCGGTGCGTTGGG

IL8	ACATGACTTCCAAGCTGGCC	CAGAAATCAGGAAGGCTGCC
MMP3	AAATTCCATGGAGCCAGG	AACTCCAACTGTGAAGATCC
NAT10	ATAGCAGCCACAAACATTCGC	ACACACATGCCGAAGGTATTG

REFERENCE

1. Robert, X. & Gouet, P. Deciphering key features in protein structures with the new ENDscript server. *Nucleic acids research* **42**, W320–W324 (2014).
2. Chan, P.P. & Lowe, T.M. GtRNAdb 2.0: an expanded database of transfer RNA genes identified in complete and draft genomes. *Nucleic acids research* **44**, D184–D189 (2016).
3. Chan, P.P. & Lowe, T.M. GtRNAdb: a database of transfer RNA genes detected in genomic sequence. *Nucleic acids research* **37**, D93–D97 (2009).
4. Ito, S. et al. A single acetylation of 18 S rRNA is essential for biogenesis of the small ribosomal subunit in Saccharomyces cerevisiae. *Journal of Biological Chemistry* **289**, 26201–26212 (2014).
5. Cheng, J., Kellner, N., Berninghausen, O., Hurt, E. & Beckmann, R. 3.2-Å-resolution structure of the 90S preribosome before A1 pre-rRNA cleavage. *Nature structural & molecular biology* **24**, 954–964 (2017).
