# Supplementary material for: Structure of the NAT10 acetyltransferase and mechanism of tRNA acetylation
Source: Nat Commun. 2026 Jun 20;17:7763. doi: 10.1038/s41467-026-74222-6 (PMC13433802; doi:10.1038/s41467-026-74222-6)
Supplement: Supplementary file 2 [file 41467_2026_74222_MOESM2_ESM.pdf]

nature portfolio

Corresponding author(s): Ronen Marmorstein, Jordan L. Meier

Last updated by author(s): Apr 1, 2026

Reporting Summary

Nature Portfolio wishes to improve the reproducibility of the work that we publish. This form provides structure for consistency and transparency in reporting. For further information on Nature Portfolio policies, see our Editorial Policies and the Editorial Policy Checklist.

Statistics

For all statistical analyses, confirm that the following items are present in the figure legend, table legend, main text, or Methods section.

n/a	Confirmed	
☐	☒	The exact sample size (*n*) for each experimental group/condition, given as a discrete number and unit of measurement
☐	☒	A statement on whether measurements were taken from distinct samples or whether the same sample was measured repeatedly
☐	☒	The statistical test(s) used AND whether they are one- or two-sided<br>*Only common tests should be described solely by name; describe more complex techniques in the Methods section.*
☒	☐	A description of all covariates tested
☒	☐	A description of any assumptions or corrections, such as tests of normality and adjustment for multiple comparisons
☐	☒	A full description of the statistical parameters including central tendency (e.g. means) or other basic estimates (e.g. regression coefficient) AND variation (e.g. standard deviation) or associated estimates of uncertainty (e.g. confidence intervals)
☐	☒	For null hypothesis testing, the test statistic (e.g. *F*, *t*, *r*) with confidence intervals, effect sizes, degrees of freedom and *P* value noted<br>*Give P values as exact values whenever suitable.*
☒	☐	For Bayesian analysis, information on the choice of priors and Markov chain Monte Carlo settings
☒	☐	For hierarchical and complex designs, identification of the appropriate level for tests and full reporting of outcomes
☒	☐	Estimates of effect sizes (e.g. Cohen's *d*, Pearson's *r*), indicating how they were calculated

Our web collection on statistics for biologists contains articles on many of the points above.

Software and code

Policy information about availability of computer code

Data collection	Thermo Scientific Titan Krios G3 was used for cryo-EM data collection; Typhoon™ laser-scanner platform was used for auto-radiography assay visualization; Applied Biosystem QuantStudio Real-Time PCR System was used for quantitative PCR measurements
Data analysis	MotionCor2, CTFFIND4, RELION 3.1.3, COOT 0.9.8.7, Chimera 1.17, ChimeraX 1.6.1 Phenix Phenix1.20.1-4487, Pymol 2.5.5, GraphPad prism 9

For manuscripts utilizing custom algorithms or software that are central to the research but not yet described in published literature, software must be made available to editors and reviewers. We strongly encourage code deposition in a community repository (e.g. GitHub). See the Nature Portfolio guidelines for submitting code & software for further information.

Data

Policy information about availability of data

All manuscripts must include a data availability statement. This statement should provide the following information, where applicable:

- Accession codes, unique identifiers, or web links for publicly available datasets
- A description of any restrictions on data availability
- For clinical datasets or third party data, please ensure that the statement adheres to our policy

The cryo-EM maps and atomic coordinates for E.coli produced recombinant CtNAT10/cytidine-acetone-CoA, E.coli produced recombinant CtNAT10/cytidine-amide-CoA/ADP and Sf9 produced recombinant CtNAT10/cytidine-amide-CoA/ADP have been deposited to the Electron Microscopy Data Bank (EMDB) (accession codes: 43996 [https://www.ebi.ac.uk/emdb/EMD-43996], 44038 [https://www.ebi.ac.uk/emdb/EMD-44038] and 44042 [https://www.ebi.ac.uk/emdb/EMD-44042],

respectively) and the Protein Data Bank (PDB) (accession codes: 9AYM [https://www.rcsb.org/structure/9AYM], 9B0E[https://www.rcsb.org/structure/9B0E] and 9B0I [https://www.rcsb.org/structure/9B0I], respectively). Other primary data files have been provided with the manuscript.

Research involving human participants, their data, or biological material

Policy information about studies with human participants or human data. See also policy information about sex, gender (identity/presentation), and sexual orientation and race, ethnicity and racism.

Reporting on sex and gender	N/A
Reporting on race, ethnicity, or other socially relevant groupings	N/A
Population characteristics	N/A
Recruitment	N/A
Ethics oversight	N/A

Note that full information on the approval of the study protocol must also be provided in the manuscript.

Field-specific reporting

Please select the one below that is the best fit for your research. If you are not sure, read the appropriate sections before making your selection.

☒ Life sciences ☐ Behavioural & social sciences ☐ Ecological, evolutionary & environmental sciences

For a reference copy of the document with all sections, see nature.com/documents/nr-reporting-summary-flat.pdf

Life sciences study design

All studies must disclose on these points even when the disclosure is negative.

Sample size	No statistical methods were used to predetermine sample size. The cryo-EM data for each sample were collected for at least one day until the data generated interpretable maps with sufficient resolutions reported in this study.
Data exclusions	To eliminate the poor particles or classes that did not yield high-quality 3D reconstructions, we carried out 2D and 3D classification for the original particle pictures. In EM field, this process is standard. The Methods section contains a description of the detail of these processes.
Replication	All biochemical and biophysical experiments were successfully reproduced. Cryo-EM data collection and analysis was performed once.
Randomization	no randomization required
Blinding	blinding was not required

Reporting for specific materials, systems and methods

We require information from authors about some types of materials, experimental systems and methods used in many studies. Here, indicate whether each material, system or method listed is relevant to your study. If you are not sure if a list item applies to your research, read the appropriate section before selecting a response.

Materials & experimental systems

n/a	Involved in the study	
☐	☒	Antibodies
☐	☒	Eukaryotic cell lines
☒	☐	Palaeontology and archaeology
☒	☐	Animals and other organisms
☒	☐	Clinical data
☒	☐	Dual use research of concern
☒	☐	Plants

Methods

n/a	Involved in the study	
☒	☐	ChIP-seq
☒	☐	Flow cytometry
☒	☐	MRI-based neuroimaging

Antibodies

Antibodies used	NAT10 (Santa Cruz Biotechnology sc-271770), GAPDH (Cell Signaling #5174S), MMP3 (Abcam #ab52915), Lamin B1 (Abcam ab16048)
Validation	NAT10 antibody was validated using NAT10 knockdown lines which showed a corresponding decrease upon immunoblot.

Eukaryotic cell lines

Policy information about cell lines and Sex and Gender in Research

Cell line source(s)	Spodoptera frugiperda (sf9) cell lines were used for recombinant protein expression; IMR90 Lung Fibroblast cells was used for senescence assays and was obtained from the Coriell Institute; S. cerevisiae strain, pSH35-c (A derivative pPK468 plasmid carrying kre33-H545A) was used for the yeast growth assays, pPK468 is a high copy number plasmid carrying URA3 marker, 2μ ori, amp (Source: Sunny Sharma, Rutgers University)
Authentication	Sf9 cells prepared in house from a master stock that was commercially bought, they were not authenticated experimentally for these studies.
Mycoplasma contamination	All cell lines are tested negative for mycoplasma contamination in 2022
Commonly misidentified lines (See ICLAC register)	Sf9 cells: Sf21, Hi5

Plants

Seed stocks	N/A
Novel plant genotypes	N/A
Authentication	N/A
