# Supplementary material for: Structure of the NAT10 acetyltransferase and mechanism of tRNA acetylation
Source: Nat Commun. 2026 Jun 20;17:7763. doi: 10.1038/s41467-026-74222-6 (PMC13433802; doi:10.1038/s41467-026-74222-6)
Supplement: Supplementary file 3 [file 41467_2026_74222_MOESM3_ESM.pdf]

nature portfolio

# Peer Review File

# Structure of the NAT10 Acetyltransferase and Mechanism of tRNA Acetylation

Corresponding Author: Dr Ronen Marmorstein

**This file contains all reviewer reports in order by version, followed by all author rebuttals in order by version.**

Version 0:

Reviewer comments:

Reviewer #1

(Remarks to the Author)
In this manuscript the authors describe efforts to solve the structure of a eukaryotic N4-cytidine acetyl transferase (NAT10) along with structure/activity relationship studies using mutations at functionally important sites identified by their structural studies. They use cryoEM to solve the structure of CtNAT10 bound to a cytidine-CoA bisubstrate probe molecule. These structures identified the binding site for ADP in the helicase domain, the binding site for CoA in the acetyl transferase domain, putative catalytic residues H548 and Y549 and positively charged patches on the protein surface as possible RNA binding sites. Their structures guided mutations in yeast Kre33 and human NAT10 and the resulting mutants were tested for temperature-dependent growth and senescence, respectively to probe the importance of specific residues in these phenotypes. The topic of defining the molecular basis for RNA modification enzymes is important. Their structures appear to be rigorously determined and follow-up experiments were well designed and executed. I have the following comments.

1) The putative “catalytic” residues H548 and Y549 were identified as conserved residues “near” the reactive cytidine. What role do the authors suggest these residues play in catalysis? Is there precedence in the study of other N-acetyl transferases for histidine or tyrosine catalytic residues? More discussion of the potential role of these residues in the acyl transfer reaction and mutations beyond alanine are justified here (e.g. Y549F, H548Q, etc).
2) While important electropositive patches are identified, the authors suggest no model for RNA binding. Can their results be used to generate a reasonable model for RNA substrate recognition that can be used to guide future experiments? Also, there appear to be other electropositive patches on the protein surface not probed by mutagenesis here (Figure 4D). Are the authors excluding these as possible sites for specific RNA substrate recognition?

Reviewer #2

(Remarks to the Author)
Please note to prepare this review we participated in the Nature Comms new Early Career Researcher review program. This report was jointly prepared by the lab head and a post-doc.

In this manuscript the authors investigate the molecular basis for RNA cytidine acetylation by the GNAT N-acetyltransferase 10 (NAT10). NAT10 is conserved across eukaryotes and has established roles in tRNA acetylation and rRNA acetylation as well as essential non-catalytic roles in promoting ribosome assembly. While there are structures of pre-ribosome bound Nat10, the molecular basis of cytidine specific RNA acetylation has remained unknown. To address this gap the authors purified recombinant CtNAT10 from both E. coli and insect cells and demonstrated that the recombinant protein is active and binds various RNAs and cytidine-CoA probes. Next the authors solved 3 cryo-EM structures of CtNat10 bound to different CoA conjugate probes. The structures support that Nat10 assembles into a heart shaped dimer with extensive dimerization interfaces. The structures also reveal the architecture of the ATP binding pocket (all structures contain ADP) and the cytidine-CoA probes. Based on electrostatic surface potential the authors proposed several RNA binding patches and confirmed the significance of these RNA binding patches along with two putative catalytic residues through a series of biochemical assays and rescue experiments in yeast. Finally, the authors investigated the role of NAT10 activity on senescence in IMR-90 cells. shRNA knockdown of NAT10 was shown to cause a reduction in senescence while overexpression of NAT10 leads to an increase in senescence. Experiments with tet inducible Het293T cell lines suggest that it’s the acetyltransferase activity of NAT10 that is responsible for promoting senescence as expression of catalytic

mutants does not lead to an increase in senescence markers.

In summary, this manuscript provides new insight into the structure and cellular roles of NAT10. We appreciate that the authors determined several NAT10 structures bound to CoA probes and complimented their structural work with in vitro and cellular assays in both yeast and human cell lines. However, several big questions remain about NAT10's catalytic activity. We still do not understand the molecular basis for cytidine-specificity or how NAT10 associates with RNA substrates. Additional work is needed to support the manuscripts title "Molecular Basis for RNA Cytidine Acetylation by Nat10".

Major Issues:
1. What does acetylation do to RNA? This is not described in the introduction or discussion and is important to establish significance. How might RNA acetylation promote stress responses (heat tolerance) and cellular senescence?

2. The authors produced recombinant CtNat10 from two different sources for structural studies but used the Sf9 expressed protein for biochemical assays. Some additional clarification on why this was done is needed. Are both recombinant proteins active? Does the Sf9 expressed protein behave differently because of potential post-translational modifications?

3. A major concern is a lack of comparison to previously determined structures of NAT10/Kre33. Is the observed CtNat10 dimer interface the same that is seen in Ct pre-90S structures (Cheng et al Mol Cell 2019). What about NAT10 pre-90S structures from S. cerevisiae and human? Neither the Results nor the Discussion make this comparison and we think it would be valuable information. The authors should also add a superposition of CtNat10 from ribosome bound structures to Figure S6.

4. Lines 170 – 171 and in Results: Please explain (near line 170 in Intro) why you were interested in comparing amide and acetone linkers. Interactions with the specific functional groups at the linker? The length/ number of bonds of the probe? It would be good to then follow up on this by adding a comment in the Results on what you learned by testing both linkers, wherever both linkers were tested, even if the result is "no discernable difference."

5. The authors suggest that dimerization of NAT10 is biologically relevant, and they propose that one dimer supports RNA binding for the catalytic active site of the other dimer. The authors should test this hypothesis by generating mutants that disrupt the dimerization interface and performing in vitro activity assays.

6. While the structures provide new insight into where ADP and CoA bind, the cryo-EM structures lack a cognate RNA substrate. Moreover, the cytidine nucleotide of the probe is poorly resolved in all of the structures. An RNA bound structure would provide significant new insight into understanding how NAT10 binds RNA as well as provide molecular details about the mechanism of RNA Cytidine acetylation. An RNA bound structure would also help test the authors hypothesis that dimerization supports RNA binding. The authors should also consider trying to capture a structure with ATP bound NAT10 via a non-hydrolysable analogue or NAT10 hydrolysis deficient mutant.

7. The authors make use of a yeast strain with a catalytically deficient Nat10 that displays growth defects at elevated temperatures, but we are confused by how these experiments were carried out. More experimental details are needed to address the issues below. Using an alternative strategy to knockdown endogenous Kre33 (such as with a Tet off promoter) would be a more straightforward way to look at the effects of Kre33 mutants on cell viability at elevated temperatures as opposed to using a cell line that already includes a mutation.
a. The authors must include a supplemental table of all yeast strains used in this study. This would help alleviate much of the confusion.
b. The authors cite Sharma et al NAR 2015 as the source of the yeast strain but there is no mention of a temperature sensitive Kre33 mutation in this paper. Is this not the correct source?
c. Were all the yeast plasmids transformed into the Kre33delta-H545A strain?
d. In the figures does H545A mean the parent strain or the Kre33delta-H545A strain transformed with a plasmid containing another copy of H545A?
e. The growth defects in Fig. S10 are minimal. It would be easier to compare if the authors put all the mutants on 1 plate and show the temperatures side by side as opposed to comparing each mutant to itself. The authors should also consider using a plate reader to determine the doubling time at 30 and 37 deg, as this would be a more precise way to compare minor growth defects.
f. Line 333-335. "WT-ScKre33 increases the growth at 37 while an empty vector does not (Figure 5B)." We do not see an obvious difference in growth between the WT and vector plates shown in Fig. S10.
g. Do the yeast kre33 plasmids listed in table S2 contain the endogenous kre33 promoter and terminator sequences or were they overexpressed?
h. Why does the final entry in table S2 read "plasmid vector control (Sckre33)". Is this an empty vector or a plasmid containing kre33?

Minor Issues:
1. It would be helpful to include a cartoon schematic of the acylation reaction as a supplemental figure to help readers understand how the synthesized probes mimic the NAT10 reaction.
2. Lines 71-72 – The authors should consider rephrasing this sentence. NAT10 is essential because of its role in ribosome assembly and not because of its catalytic activity.
3. Line 95: In what way might NAT10 be a viable therapeutic target? This should be linked to NAT10's known activities (ribosome biogenesis, stress response, senescence, aging, and/or cancer) a little more explicitly. As is, this paragraph is

gesturing a little vaguely at reasons why NAT10 might be important, in a way that actually makes the significance of the paper's results a little less accessible.
4. Line 166: For "bisubstrate probes" could you more explicitly describe which part of the probe goes into which active site/ interacts with which domain?
5. Line 174/ Figure 1C: Did you test thermal stability with acetone linker? Why was this excluded?
6. Lines 185 – 186, Supplemental Figure 3: Please add an image of the map after refinement steps but before C2 symmetry was applied, or note at exactly what step C2 symmetry was applied. Did the authors try masking half of the dimer and using symmetry expansion, or further processing what became their final map with 3D classification or 3D variability – have you ruled out the possibility that the two halves of the dimer are not perfectly symmetrical? Is it required that both ATP/ ADP and acetyl-CoA be bound in both dimers?
7. Line 210/ Figure 2D: Please label the peak that corresponds to the dimer as "dimer." Is the larger peak representative of a tetramer?
8. Line 338: In this line, please name the specific basic patch 2 mutants you are referring to. It is not straightforward to determine which mutants you mean here just by looking at Supplementary Figure 10.
9. Figure 4 and Supplemental Figure 10: Please label residues according to patch number. Color coding in Supplemental Figure 10 to be consistent with Figure 4 would be nice but labels alone would be sufficient (guaranteed to be clear even for those who are color blind).
10. Figure 4C: The green selection outline in 4C that highlights residues H548 and Y549 makes these atoms look green instead of yellow. Could you change the selection outline to either black or bolded yellow?
11. Supplemental Fig 2 – it would be very helpful to add the domain labels/boundaries onto the sequence alignment as well as indicate the catalytic residues and putative RNA binding patches.
12. Lines 573 – 576: At what point in this elution protocol does your product elute?
13. Line 631 – Were these RNAs folded or just resuspended in buffer?

Reviewer #3

(Remarks to the Author)
I co-reviewed this manuscript with one of the reviewers who provided the listed reports. This is part of the Nature Communications initiative to facilitate training in peer review and to provide appropriate recognition for Early Career Researchers who co-review manuscripts.

Reviewer #4

(Remarks to the Author)
In this study, Zhou and colleagues aim to structurally and functionally analyse the eukaryotic RNA N4-cytidine acetyltransferase, NAT10. NAT10 catalyses cytidine acetylation of different RNA substrates, including 18S rRNA and tRNAs. The role of NAT10 during ribosome biogenesis has recently been complemented by cryo-EM structures of 90S pre-ribosomes in complex with NAT10. These structures and previous structures of the bacterial homologue TmcA revealed the overall architecture and domain organization of dimeric NAT10. The specific functional role of ac4C in eukaryotic tRNAs has remained elusive and our understanding of how NAT10 targets specific tRNAs and positions in different RNA targets is very limited. The pathway seems to play a crucial role in biological/cellular functions (e.g. translation, senescence) and clinical data links mutations in the components to severe human pathologies. Hence, the study aims to answer interesting biological questions of a highly relevant and timely topic.

The authors purify eukaryotic NAT10 from the thermophilic fungus Chaetomium thermophilum and determine its single particle cryo-EM structure bound to a cytidine-CoA probe with and without ATP. They analyse the obtained structures and complement their work with functional analyses of structure-guided mutants. The structural work is carried out a state-of-the-art level and generally I don't see any major technical issues with any of the experimental approaches. However, I have major reservations about the relevance and impact of the obtained structural snapshots.
I appreciate the efforts of the authors to identify/map the respective regions in NAT10 that mediate tRNA binding – especially in the absence of a tRNA- and /or Tan1-bound structure. I was wondering whether the authors have tried to obtain such structures (with full tRNA or ASL) and whether they have any complementary data (SAXS, cross-linking MS, …) that would provide additional restraints and allow to generate a model of the complex during the catalytic step of the modification reaction. At the moment, it cannot be excluded that the complex undergoes major structural rearrangements during tRNA and/or Tan1 binding, whether the complex binds 1 or 2 tRNAs molecules at the same time and whether the mutated residues are anywhere close to the substrate. The lack of this kind of data represents a significant gap that clearly limits the impact of the study and might be more confusing than revealing – in particular, as the observed dimeric conformation has in principle been observed already in the pre-ribosome structures. I also think, that the results of the mutational analyses are rather confusing and misleading – I assume, that the role of the mutated residues can only be clarified/interpretated after obtaining a tRNA- and/or Tan1-bound structure of NAT10. At the moment this does not represent an informed structure guided approach.
Therefore, I am afraid that I am not able to support publication of the manuscript in its current form at Nature Communications. I encourage the authors to consider some of my comments for their future work and/or before finding a different target journal for the work.

Major
1. First, a more quantitative assay (e.g. SPR, MST, FP) to measure the RNA binding affinity is need to more quantitatively compare the various point mutants (Suppl. Fig. 9). For instance, the authors mention "Interestingly, none of the mutations

significantly compromised RNA binding capability (Supplemental Figure 9)" – at the moment, this could result from the identification of the wrong residues or the lack of sensitivity of the assay – or both. The authors should also include a non-substrate tRNA and another folded RNA as negative controls (Fig. 1B).

2. In general, the results from the used functional assays are highly inconsistent and present a rather confuse picture. The authors do mention this themselves - e.g. "Interestingly, one patch 3 mutant (hR421A/CtR422A) and patch 2 mutants (hY835A/CtY851A and hH836A/CtH852A) in both the human cell-based senescence assays and the yeast cell-based fitness assay scored as WT or near WT, but were either inactive or impaired in CtNAT10 biochemical assays, suggesting some differences between the in vitro and cellular systems." It seems very strange that mutations that were chosen due to there possible involvement in tRNA binding, do not "significantly compromised RNA (Supplemental Figure 9)" at all – but somehow they all affect the activity. Furthermore, in yeast mutants in "basic patches 1 and 3 failed to rescue the temperature sensitivity, while basic patch 2 mutants did show some rescue (Figure 5B and Supplemental Figure 10)" – in human cells, only one "patch 3 mutant and the active site mutants exhibited a markedly decreased p16 expression, while the rest of the mutants recapitulated the effect of WT-NAT10" – hence, the inconsistencies go far beyond what has been mentioned. I simply don't see that the authors managed to bring the structural and functional data together and this part is very confusing to the reader.

3. The authors need to show the comparison between the free and pre-ribosome bound Ct/ScNAT10 complexes. At the moment they state that their analyses shows the homodimer forms already in solution, but it would be enlightening to see if any (even small) structural changes can be observed between the free and pre-ribosome bound NAT10 structures. I am not sure whether the authors checked the deposited maps of the pre-ribosome structures for the presence of ATP, acetyl-CoA or other intermediates in the respective active sites – this needs to be analysed to allow for a fair comparison.

4. Towards a structural analyses of the tRNA-bound state, the authors should check the thermostability in the presence of ATP/ATP-analogues/acetyl-CoA and RNA substrate. They should also quantitatively measure the effect of ATP/ATP-analogues/acetyl-CoA on RNA binding.

5. Please provide the non-symmetrized reconstructions in the manuscript – this is particularly important as the lower signal of the ligands in the biding sites might result from lower overall occupancy or the presence of a ligand molecule in only one of the active sites at a time, which would have far-reaching functional implications – leading to the assumption of an asymmetric complex that can only modify one tRNA at a time.

6. I am not convinced that the obtained structures of the cytidine-CoA probes do represent catalytically relevant states at all and rather reflect the pre-reaction acetyl-CoA bound that has previously been reported already. The authors do not show whether there are any differences in the coordination of the probes and the previously determined structure of TmcA bound to acetyl-CoA and ADP – in my opinion the scientific insights are very limited. The author do mention that "Inspection of the active site proximal to the cytidine nucleotide reveals a somewhat open pocket with no residues in proximity to make direct contacts suggesting that a structural rearrangement may be required for N4-cytidine acetylation", but I think the current text of the manuscript overstates the novelty of the resolved structures. I believe the authors designed their study to observe specific reaction intermediates, but ended up with the already known states. The analyses of the fungal CtNAT10 bound to acetyl-CoA and ADP might be more relevant than the presented structures – however, also less exciting of course.

Minor

• It was not mentioned whether there was any specific reason of determining the different structures from bacterial or insect cell source. It might just have happened that way, but it would be nice to comment on why the authors went for both expression strategies and if there was any specific rationale.
• What happened in suppl. Figure 9a at concentration 0.81uM wt and R422A?
• Which steps of the processing (Suppl. Fig. 3) were performed with binned and unbinned particles. I would refer to "picks" rather than particles before the initial 2D classification step – I doubt the author excluded 5.500.000 real protein "particles".
• It is clear from the alignment that the residues that form the dimerization interface are not conserved/present in bacterial TmcA. It would be nice for the reader to show a structural representation of this analysis (similar to Fig. 2C).
• I don't understand the representation and description of Figure 2F – this needs to be improved. Could the authors speculate whether the difference in the position of the RNA binding domain between NAT10 and TmcA could be caused by crystal contacts in the bacterial structure or the homodimerization – I simply got confused by the figure and the interpretation – "Interestingly, the RNA binding domain of the symmetry related subunit superimposes much more closely to the position of the RNA binding domain of EcTmcA (Figure 2F), thus further supporting the biological importance of the dimeric state of NAT10." The position of the domain is similar in the pre-ribosome bound structure, right – please add a comment.
• "In the CtNAT10/cytidine-amide-CoA/ADP structure, the ADP and $Mg^{2+}$ are wedged in between the helicase and DUF domains, and most of the protein interactions are mediated by the α-helix bundle (α9-α12) from the helicase domain (Supplemental Figure 7)." – I think the reference to this figure is suboptimal and the information could be included in Figure 4B (or 4A).
• Figure 3A – ATP hydrolysis and magnesium coordination. In my opinion, the map interpretation is highly speculative and should be complemented by ATP hydrolysis measurements – or the section should at least be rephrased, as it is rather based on previous observations by others than the actual data of this study.

Version 1:

Reviewer comments:

Reviewer #2

(Remarks to the Author)
Please note to prepare this review we participated in the Nature Comms new Early Career Researcher review program.

The authors have adequately addressed the majority of our previous concerns. We appreciate the additional assays including the demonstration that THUMPD1 enhances activity towards cognate tRNAs and the new data that supports specificity of RNA acetylation reflects differential catalytic efficiency rather than differences in tRNA binding affinity. We also appreciate the improved description of the yeast-based assays.

Minor Issues:
1. We find Figure 1A hard to follow and do not agree with the authors designation of positive and negative controls. The positive control is not a control but rather the reaction being studied with all the components present. We suggest coming up with a better format for this figure – perhaps separating the end point and time courses rather than showing them side-by-side. Using the format to designate what components are present from Supplemental Fig. 4B would also be helpful.
2. In Figures 2-4 it is not always clear which of the three determined structures are shown in each panel. It would be helpful to readers to add the PDB codes to the figure legends to make this more clear. For example, in Fig. 3A is the ADP density from the Sf9 or EC structure? Are 3A and 3B from the same structure? If so it would be better to use the same color for ADP and Mg in both.

Reviewer #4

(Remarks to the Author)
I have carefully reviewed the revised manuscript, now entitled “Structure of the NAT10 Acetyltransferase and Mechanism of RNA Acetylation”.

The authors have performed additional experiments and carefully rephrased the manuscript. In addition, the focus shifted away from the activity of NAT10 on rRNA towards the role of NAT10 in tRNA acetylation.
I think the current text of the manuscript improved on down-toning previous overstatements about the novelty of the resolved structures. I recognize that the authors designed their study to observe specific reaction intermediates, but ended up with the already known states. I repeat myself from the first round of reviews – “The analyses of the fungal CtNAT10 bound to acetyl-CoA and ADP might be more relevant than the presented structures – however, also less exciting of course.” In my opinion, some interesting results were included, but they not necessarily validated previous claims/assumptions – rather the authors removed the previously acquired dataset and replaced it with another apparently more consistent one. For all of the above reasons, the number of presented results increased, but the consistency between the results did not necessarily follow this trend. In my opinion, the actual scientific advances described in the manuscript still does not justify publication in Nature Communications.

Major issues
1. The authors state that “While our cytidine-CoA probes were designed to capture a transition state intermediate, the structure as observed likely represents a pre-reaction state, since the cytidine portion is partially disordered and the essential catalytic residues (CtH548 and CtY549) are relatively far from the exocyclic N4 amine. This suggests that significant conformational rearrangements are required to bring the catalytic machinery into proper geometry for N4-cytidine acetylation.” Would the nanoDSF data not indicate the opposite, showing that neither tRNA nor THUMPD1 binding to CtNAT10 have significant effects on structure. Either one of the techniques is not appropriate or there something else that is incorrectly interpreted/analyzed.
2. It is interesting to see that the newly incorporated data on the tRNAs modification activity is much clearer than for the previously used rRNA. Considering that the set of mutants reacts so differently to the two types of RNAs, I was wondering what is so different with these substrates – the logic of basic patches binding RNAs should be valid for tRNAs as well as rRNAs. One question – what is the scientific reason for excluding the previous analyses/data for the rRNA?
3. The authors state correctly “This is consistent with these cellular phenotypes being associated with defects in tRNA acetylation.” Now I start to wonder, why NAT10 would be associated with ribosome biogenesis at all, if the phenotypes correlate to well only with the results from the tRNAs. Do the authors think that was a technical problem with the used rRNA and if they knew that probably a snoRNA is needed, why did they not try include it (or why did include the previous data into the first version of the manuscript)?
4. Foremost, the new data with THUMPD1 (Fig. 1) clearly ask for some interaction assays and at least a structural prediction of the complex. In addition, the use of different concentrations of NAT10 and THUMPD1 in the presented EMSAs (Supplemental Figure 4) are slightly confusing. Foremost, I was wondering how the authors interpret the results of the “complex” at high concentrations (e.g. 1750, 3500 and 7000 nM) – it looks like the complex gets smaller again. This leads me to the other question – do they form a complex at all or do they just bind the same tRNA molecule. The authors seem to assume this by stating – “This result underscores the rate acceleration conferred by THUMPD1 as well as the substrate specificity of the NAT10–THUMPD1 complex towards tRNA.” – however I am missing any experimental evidence for the presence of the “complex”. Here several question arise from the interesting data (e.g. do the basic patch mutants still interact with THUMPD1) and I am inclined to say that additional work on this complex would make a much more interesting story – I am sure the authors are also aware of this.
5. As the results from the mutants are now consistent between the in vitro modification activity and the cellular assays, it would be crucial to measure the actual tRNA modification levels in the yeast and human cells to directly correlate the phenotypes with decreased/unchanged tRNA modification levels.

Minor issues:
• “rRNA acetylation could also play an essential role in ribosome biogenesis. For this reason, we decided to replace our functional in vitro data with assaying tRNA acetylation (an activity known to be non-essential) in the presence of the THUMPD1 cofactor protein”…I don’t follow the logic in linking these two statements?

• The number of picks/initial particles is inconsistent between Table 1 and Supplemental Figure 5.
• Page 2 – "acetylation is catalyzed by a single" something weird with the font size
• The following part of the introduction is highly speculative – "In addition, the substrate-spectrum of NAT10 is unclear. While NAT10's ability to catalyze the deposition of ac4C in ribosomal RNA 108 (rRNA) and transfer RNA (tRNA) are well-validated, other protein and RNA substrates remain topics of inquiry. Outside of the association of tRNA acetylation with neurodevelopmental defects, what specific RNA substrates mediate individual disease phenotypes is largely unknown 32. Altogether, these data highlight a need for molecular-scale characterization data capable of providing domain and substrate specific insights into NAT10 functions." and I don't understand how the current study helps to resolve this issues – by deleting the rRNA data it becomes even more limited -

Other issues
I have also read the responses to the other reviewers and a couple of specific points caught my attention.
• "due to current restrictions at the NIH we are unable explore the alternative strategy suggested by the reviewers" – why would this ever be a reasonable explanation to not perform an experiment – worst case, you have to as somebody that does not have those restrictions to perform the experiment for you.
• "We unfortunately depleted our supply of the probe containing the acetone linker so could not test the thermal stability of the probe." This sounds like the authors are not able to challenge, reproduce or validate any data that is related to the probe. In this case, the data should probably be taken out of the manuscript.
• "For clarification, patch numbers have been added to Figure 4F and color-coded in Supplemental Figures 10." – Figure 4F does not exist and am not sure which panel in Supplemental Figure 10 is the relevant one?

Version 2:

Reviewer comments:

Reviewer #2

(Remarks to the Author)
The authors have addressed my previous concerns and I am supportive of publication.

Reviewer #4

(Remarks to the Author)
I have carefully reviewed the rerevised manuscript entitled "Structure of the NAT10 Acetyltransferase and Mechanism of RNA Acetylation". In the rerevised version, the authors have put additional efforts in addressing and answering all issues raised. I appreciate the newly provided data and the explanations and I support publication of the revised manuscript in Nature Communications.

As the focus shifted away from the activity of NAT10 on rRNA and towards the role of NAT10 in tRNA acetylation, I would suggest to change the title to "Structure of the NAT10 Acetyltransferase and Mechanism of tRNA Acetylation".

Finally, I would like to congratulate the authors for this comprehensive piece of work and I am looking forward to seeing the follow-up results on the newly defined NAT10–THUMPD1 complex – based on the finding described in this manuscript.

**Open Access** This Peer Review File is licensed under a Creative Commons Attribution 4.0 International License, which permits use, sharing, adaptation, distribution and reproduction in any medium or format, as long as you give appropriate credit to the original author(s) and the source, provide a link to the Creative Commons license, and indicate if changes were made.
In cases where reviewers are anonymous, credit should be given to 'Anonymous Referee' and the source.
The images or other third party material in this Peer Review File are included in the article's Creative Commons license, unless indicated otherwise in a credit line to the material. If material is not included in the article's Creative Commons license and your intended use is not permitted by statutory regulation or exceeds the permitted use, you will need to obtain permission directly from the copyright holder.
To view a copy of this license, visit https://creativecommons.org/licenses/by/4.0/

**Response to Reviewers**

Reviewer #1 (Remarks to the Author):

In this manuscript the authors describe efforts to solve the structure of a eukaryotic N4-cytidine acetyl transferase (NAT10) along with structure/activity relationship studies using mutations at functionally important sites identified by their structural studies. They use cryoEM to solve the structure of CtNAT10 bound to a cytidine-CoA bisubstrate probe molecule. These structures identified the binding site for ADP in the helicase domain, the binding site for CoA in the acetyl transferase domain, putative catalytic residues H548 and Y549 and positively charged patches on the protein surface as possible RNA binding sites. Their structures guided mutations in yeast Kre33 and human NAT10 and the resulting mutants were tested for temperature-dependent growth and senescence, respectively to probe the importance of specific residues in these phenotypes. The topic of defining the molecular basis for RNA modification enzymes is important. Their structures appear to be rigorously determined, and follow-up experiments were well designed and executed. I have the following comments.

1) The putative "catalytic" residues H548 and Y549 were identified as conserved residues "near" the reactive cytidine. What role do the authors suggest these residues play in catalysis? Is there precedence in the study of other N-acetyl transferases for histidine or tyrosine catalytic residues? More discussion of the potential role of these residues in the acyl transfer reaction and mutations beyond alanine are justified here (e.g. Y549F, H548Q, etc).

There is a strong precedence for histidine, tyrosine and glutamate residues, among others, playing important catalytic roles, as either general bases or acids, in protein N-acetyltransferases such as histone acetyltransferases (HATs) and N-terminal acetyltransferases (NATs). Given the proximity of H548 and Y549 to where chemistry is likely to occur, we hypothesized that they also play similar catalytic roles in Nat10. This point is now elaborated in the results section.

The relevant test is:

"Consistent with this possibility, the only two residues within ~13 Å of the cytidine N4 that could play a role in chemistry are His548 and Tyr549, which sit on a loop between helices α18 and α19 of the GNAT domain. This is consistent with the use of His, Tyr and Glu residues that are typically used as general base residues for catalysis by GNAT proteins [3,43]."

The reviewer brings up a good point that in order to make stronger argument for the catalytic roles of H548 and Y549, we should evaluate the more conservative H548Q and Y549F mutants to complement the H548A and Y549A mutants that we have already evaluated. We have now prepared the H548Q and Y549F mutants and demonstrated that, like the H548A and Y549A mutants, these mutants result in background levels of catalytic activity towards cognate tRNA substrates (**Figure 4E**), thus more strongly supporting the catalytic roles of these residues.

2) While important electropositive patches are identified, the authors suggest no model for RNA binding. Can their results be used to generate a reasonable model for RNA substrate recognition that can be used to guide future experiments? Also, there appear to be other electropositive patches on the protein surface not probed by mutagenesis here (Figure 4D). Are the authors excluding these as possible sites for specific RNA substrate recognition?

We had attempted to produce alpha-fold models of Nat10 bound to tRNA and rRNA substrates but were unable to produce solutions with high confidence. So instead, we decided to probe two regions implicated by this study, or previously implicated to be involved in RNA binding: a region proximal to the position of the cytidine base for acetylation (patch 1) or a basic patch on the putative RNA binding domain (patch2). We also probed a basic patch on the opposing subunit of the dimer to test the hypothesis that the RNA reaches over to the opposing subunit. This rationale is now explained in the results section.

The relevant test is:

"To gain further insights into potential RNA binding models, we attempted to generate AlphaFold models of *Ct*NAT10 bound to various substrates combinations, including THUMPD1, tRNA, and rRNA (data no shown). However, we were unable to obtain high-confidence predictions, as indicated by chain-pair Predicted Aligned Error (PAE) values generally above 5 Å, except for the *Ct*NAT10 dimer itself. Consequently, we considered these computational models unsuitable for guiding further experiments."

To biochemically probe for potential RNA binding surfaces on *Ct*NAT10, we targeted three electropositive patches…"

Reviewer #2 (Remarks to the Author):

Please note to prepare this review we participated in the Nature Comms new Early Career Researcher review program. This report was jointly prepared by the lab head and a post-doc.

In this manuscript the authors investigate the molecular basis for RNA cytidine acetylation by the GNAT N-acetyltransferase 10 (NAT10). NAT10 is conserved across eukaryotes and has established roles in tRNA acetylation and rRNA acetylation as well as essential non-catalytic roles in promoting ribosome assembly. While there are structures of pre-ribosome bound Nat10, the molecular basis of cytidine specific RNA acetylation has remained unknown. To address this gap the authors purified recombinant CtNAT10 from both E. coli and insect cells and demonstrated that the recombinant protein is active and binds various RNAs and cytidine-CoA probes. Next the authors solved 3 cryo-EM structures of CtNat10 bound to different CoA conjugate probes. The structures support that Nat10 assembles into a heart shaped dimer with extensive dimerization interfaces. The structures also reveal the architecture of the ATP binding pocket (all structures contain ADP) and the cytidine-CoA probes. Based on electrostatic surface potential the authors proposed several RNA binding patches and confirmed the significance of these RNA binding patches along with two putative catalytic residues through a series of biochemical assays and rescue experiments in yeast. Finally, the authors investigated the role of NAT10 activity on senescence in IMR-90 cells. shRNA knockdown of NAT10 was shown to cause a reduction in senescence while overexpression of NAT10 leads to an increase in senescence. Experiments with tet inducible Het293T cell lines suggest that it's the acetyltransferase activity of NAT10 that is responsible for promoting senescence as expression of catalytic mutants does not lead to an increase in senescence markers.

In summary, this manuscript provides new insight into the structure and cellular roles of NAT10. We appreciate that the authors determined several NAT10 structures bound to CoA probes and complimented their structural work with in vitro and cellular assays in both yeast

and human cell lines. However, several big questions remain about NAT10's catalytic activity. We still do not understand the molecular basis for cytidine-specificity or how NAT10 associates with RNA substrates. Additional work is needed to support the manuscripts title "Molecular Basis for RNA Cytidine Acetylation by Nat10".

The review makes a fair point that the manuscript does not fully support the title, so we have changed the title to more accurately reflect the findings of the manuscript to "Structure of the NAT10 Acetyltransferase and Mechanism of RNA Acetylation."

While we also agree with the review that many questions remain about how NAT10 acetylates RNA, we believe that current study makes significant contributions to the field. The major findings include **(1) The first structure of NAT10 in its catalytically competent state.** Previous structural studies captured NAT10 bound to pre-ribosomes in a non-catalytic context related to RNA assembly functions. In contrast, our study reveals NAT10 in its active, engaged conformation, with bisubstrate probes occupying both cytidine and CoA binding sites. **(2) Structure-guided identification and validation of catalytic residues.** We use our structural knowledge to guide functional studies directly demonstrating that NAT10 acetyltransferase activity is required for thermoadaptation and cellular senescence. **(3) Adapter-dependent tRNA substrate discrimination (data new to this revision).** Biochemical reconstitution demonstrates that THUMPD1 specifically enhances NAT10 activity towards cognate ($tRNA_{Ser/Leu}$) but not non-cognate ($tRNA_{Ala}$) substrates. **(4) The specificity of RNA acetylation reflects differential catalytic efficiency rather than binding affinity (data new to this revision).** We demonstrate that NAT10 binds cognate and non-cognate tRNAs with similar affinity, but selectively acetylates only cognate substrates, a mechanism of RNA modification enzyme specificity that may apply broadly across this enzyme family. We have attempted to better highlight these novel findings in the revised manuscript.

Major Issues:

1. What does acetylation do to RNA? This is not described in the introduction or discussion and is important to establish significance. How might RNA acetylation promote stress responses (heat tolerance) and cellular senescence?

NAT10 appears to implement a variety of catalytic and non-catalytic functions, and their specific roles in heat tolerance and cellular senescence have yet to be conclusively determined. Two of the most conclusive properties are the ability to stabilize RNA duplexes and modulate tRNA stability and stress via ribosome pausing, which we now directly describe in the revised introduction.

The relevant text is:

"Biophysical analyses have shown that N4-acetylation of cytidine thermodynamically stabilizes duplex RNA, including within tRNA D-arm surrogates [21]. Consistent with this, disruption of eukaryotic tRNA acetylation has been found to decrease Leu/Ser tRNA stability, increase ribosome pausing, and activate stress response pathways associated with ribosome collisions [22,23]."

2. The authors produced recombinant CtNat10 from two different sources for structural studies but used the Sf9 expressed protein for biochemical assays. Some additional clarification on why this was done is needed. Are both recombinant proteins active? Does the Sf9 expressed protein behave differently because of potential post-translational modifications?

In the revised manuscript, we now more clearly describe that active CtNAT10 protein could be expressed in both E. coli and insect cell systems and was used interchangeably for structural studies. Since the insect cell protein showed slightly higher activity. it was used for all activity assays.

In the methods section, we also indicate that both E.coli and insect cell produced proteins were subjects to LC-MS/MS and showed no notable differences in PTMs.

3. A major concern is a lack of comparison to previously determined structures of NAT10/Kre33. Is the observed CtNat10 dimer interface the same that is seen in Ct pre-90S structures (Cheng et al Mol Cell 2019). What about NAT10 pre-90S structures from S. cerevisiae and human? Neither the Results nor the Discussion make this comparison and we think it would be valuable information. The authors should also add a superposition of CtNat10 from ribosome bound structures to Figure S6.

We agree with the reviewer that a comparison of our NAT10 structure with those captured bound to the pre-90S ribosome is warranted and we have now described this comparison in the discussion and include superpositions in **Supplemental Figure 8**.

4. Lines 170 – 171 and in Results: Please explain (near line 170 in Intro) why you were interested in comparing amide and acetone linkers. Interactions with the specific functional groups at the linker? The length/ number of bonds of the probe? It would be good to then follow up on this by adding a comment in the Results on what you learned by testing both linkers, wherever both linkers were tested, even if the result is “no discernable difference.”

To explain our rationale for testing different linkers in the cytidine-CoA bisubstrate probes we have revised the Results section.

The relevant test is:

“Bisubstrate probes, designed to simultaneously occupy cofactor and substrate sites have proven valuable tools for studying acetyltransferase enzyme function.[36-38] To test whether this strategy could be potentially extended to RNA acetyltransferases, we designed and synthesized two compounds as possible mimics to the transition state, cytidine-acetone-CoA and cytidine-amide-CoA (**Figure 1D**), whose structures differ only in the presence of an acetone or amide linker, respectively. The cytidine-amide-CoA probe (46.4 ± 0.1 °C) demonstrated thermal stabilization comparable to acetyl-CoA, suggesting a similar mode of interaction and a minimal contribution of the cytidine to binding energy (**Figure 1C**).”

We also have added the requested comment on the bisubstrate inhibitors to the Results section.

The relevant test is:
“Interestingly, our structural and biochemical analyses revealed no significant differences in binding mode or protein stabilization between the amide and acetone linkers, suggesting the nature of the connecting region has minimal impact on probe recognition.  Given the structural similarity between the two probe-bound complexes, all subsequent analyses were performed using the *Ct*NAT10/cytidine-amide-CoA/ADP structure.”

5. The authors suggest that dimerization of NAT10 is biologically relevant, and they propose that one dimer supports RNA binding for the catalytic active site of the other dimer. The authors should test this hypothesis by generating mutants that disrupt the dimerization interface and performing in vitro activity assays.

This is an interesting proposal by the reviewer. However, we reasoned that deletion of the regions that stabilize dimerization (including the putative RNA binding domain) would also destabilize the protein and disrupt the integrity of the active site, so any effect on acetyltransferase activity might be difficult to interpret. We therefore chose to instead prepare site directed mutations in the putative RNA binding domain that we found to perturb acetyltransferase activity and phenotypes in yeast and human cells (**Figures 4E, 5C and 6E**). Interestingly, a recent study reporting on programable RNA acetylation with CRISPR-Cas13 report on the preparation of human NAT10 C-terminal deletion mutant that removes the nuclear localization signal and most of the putative RNA binding domain and dimerization interface (https://doi.org/10.1038/s41589-025-01922-3) resulting in the preparation of a protein with hyperactive activity towards mRNA, which is not recognized as a major NAT10 target, further implicating NAT10 dimerization and the putative RNA binding region for normal cognate acetyltransferase activity. This recent study is now described in the Discussion section.

6. While the structures provide new insight into where ADP and CoA bind, the cryo-EM structures lack a cognate RNA substrate. Moreover, the cytidine nucleotide of the probe is poorly resolved in all of the structures. An RNA bound structure would provide significant new insight into understanding how NAT10 binds RNA as well as provide molecular details about the mechanism of RNA Cytidine acetylation. An RNA bound structure would also help test the authors hypothesis that dimerization supports RNA binding. The authors should also consider trying to capture a structure with ATP bound NAT10 via a non-hydrolysable analogue or NAT10 hydrolysis deficient mutant.

Although capturing and RNA bound structure is indeed a goal of ours, we believe that this goes beyond the scope of the current study. We believe that determining the structure if NAT10 bound to bisubstrate probes and the associated biochemical and cellular data now provided in the revised manuscript (see our response to the summary of the reviewer above) provides new knowledge regarding the acetylation activity of NAT10. Previous studies of NAT10 bound to pre-ribosomes, instead focused on the RNA assembly activity of NAT10.

We would like to emphasize that in the revised manuscript, we have now expanded the scope of our current studies to specifically probe tRNA acetylation by NAT10 and the role played by the THUMPD1 cofactor in this process (see revised **Figures 1A, 1B; Figure 4E; Supplemental Figure 3; Supplemental Figure 4**). We hope that this additional data also convinces the reviewer of the significant new information provided by this study.

7. The authors make use of a yeast strain with a catalytically deficient Nat10 that displays growth defects at elevated temperatures, but we are confused by how these experiments were carried out. More experimental details are needed to address the issues below. Using an alternative strategy to knockdown endogenous Kre33 (such as with a Tet off promoter) would be a more straightforward way to look at the effects of Kre33 mutants on cell viability at elevated temperatures as opposed to using a cell line that already includes a mutation.

Unfortunately, due to current restrictions at the NIH we are unable explore the alternative strategy suggested by the reviewers; however, we appreciate the request to clarify our

experimental procedure and have described our revisions to address the requested questions below.

a. The authors must include a supplemental table of all yeast strains used in this study. This would help alleviate much of the confusion.

We have added tables listing all yeast strains (**Table 2**) and related plasmid (**Table 3**) used in this study, including their genotypes and sources.

**Table S2. Yeast strains used in this study**

| **Strain Name** | **Genotype** | **Description** | **Source** |
|---|---|---|---|
| Y22944 | BY4743 Mat a/α his3Δ1/his3Δ1 leu2Δ0/leu2Δ0 lys2Δ0/LYS2 MET15/met15Δ0 ura3Δ0/ura3Δ0 YNL132w::kanMX4/YNL132w | Heterozygous KRE33 diploid strain, not used in this study | Euroscarf |
| Δkre33 + pSH35-C | Haploid derivative of Y22944: Mat a his3Δ1 leu2Δ0 met15Δ0 ura3Δ0 YNL132w::kanMX4 [pSH35-C] | Strain with endogenous KRE33 deleted, maintained with plasmid expressing catalytically deficient Kre33-H545A (GCA codon) | Sharma et al., 2015 |

**Table S3. Plasmids used in this study**

| **Plasmid Name** | **Description** | **Selection Marker** | **Source** |
|---|---|---|---|
| HQHQHH-Kre33 NM_01182970.1/pD1237 | Wild-type ScKre33 expressed from TDH3 promoter and CYC1 terminator | HIS3 | This study |
| HQHQHH-Kre33 H545A/pD1237 | Catalytic mutant (GCT codon) | HIS3 | This study |
| HQHQHH-Kre33 Y546A/pD1237 | Active site mutant | HIS3 | This study |
| HQHQHH-Kre33 K547A/pD1237 | Basic patch 1 mutant | HIS3 | This study |
| HQHQHH-Kre33 R637A/pD1237 | Basic patch 1 mutant | HIS3 | This study |
| HQHQHH-Kre33 N752A/pD1237 | Basic patch 1 mutant | HIS3 | This study |
| HQHQHH-Kre33 R420A/pD1237 | Basic patch 3 mutant | HIS3 | This study |
| HQHQHH-Kre33 K425A/pD1237 | Basic patch 3 mutant | HIS3 | This study |
| HQHQHH-Kre33 Y852A/pD1237 | Basic patch 2 mutant | HIS3 | This study |
| HQHQHH-Kre33 H853A/pD1237 | Basic patch 2 mutant | HIS3 | This study |

| Plasmid Name | Description | Selection Marker | Source |
|---|---|---|---|
| pD1237 | Empty vector control | HIS3 | This study |

b. The authors cite Sharma et al NAR 2015 as the source of the yeast strain but there is no mention of a temperature sensitive Kre33 mutation in this paper. Is this not the correct source?

This is correct, the parent strain used in our experiments is derived from the work of Sharma et al. 2015. In this strain the endogenous KRE33 gene has been deleted and replaced with a kanamycin resistance marker (YNL132w::kanMX4), and the strain was rescued with a plasmid (pSH35-C) carrying the catalytically deficient kre33-H545A allele under control of a constitutive TDH3 promoter and a URA3 selection marker.

c. Were all the yeast plasmids transformed into the Kre33delta-H545A strain?

Yes, all yeast plasmids were transformed into the Kre33delta-H545A strain (Δkre33 + pSH35-C). This strain has the endogenous KRE33 deleted from the genome and as described above expresses the the catalytically deficient kre33-H545A allele under control of a constitutive TDH3 promoter and a URA3 selection marker.

d. In the figures does H545A mean the parent strain or the Kre33delta-H545A strain transformed with a plasmid containing another copy of H545A?

Yes, in the figures, H545A refers to the Kre33delta-H545A parent strain transformed with a plasmid containing another copy of H545A. We deliberately transformed with a plasmid containing the H545A allele (rather than leaving this strain with just an empty vector) to maintain consistency across our experimental conditions, where all strains were transformed with a plasmid expressing a Kre33 variant. Of note, we can confirm the plasmid-expressed version since the parent strain has a GCA codon for Ala at position 545, whereas the transformed plasmid contains a GCT codon for Ala at the same position. This approach confirmed our expectation that the catalytically deficient H545A mutant would not rescue the temperature-sensitive phenotype. We have clarified this distinction in the revised Methods section (see response to h below).

e. The growth defects in Fig. S10 are minimal. It would be easier to compare if the authors put all the mutants on 1 plate and show the temperatures side by side as opposed to comparing each mutant to itself. The authors should also consider using a plate reader to determine the doubling time at 30 and 37 deg, as this would be a more precise way to compare minor growth defects.

While the clear rescue of growth at elevated temperature by wild-type KRE33 but not catalytically inactive variants validates the utility of this experimental system, we completely agree that the growth defects are not quantitative and as such have attempted to describe them using qualitative nomenclature (++, +, etc) in revised **Figure 5**.

f. Line 333-335. “WT-ScKre33 increases the growth at 37 while an empty vector does not (Figure 5B).” We do not see an obvious difference in growth between the WT and vector plates shown in Fig. S10.

We acknowledge that the growth difference between WT and vector control at 37°C in the originally presented Figure S10 may not be immediately obvious. They are more apparent at the 10^-2 and 10^-3 dilutions.

g. Do the yeast kre33 plasmids listed in table S2 contain the endogenous kre33 promoter and terminator sequences or were they overexpressed?

The yeast KRE33 plasmids listed in Table S2 do not contain the endogenous KRE33 promoter and terminator sequences. Instead, the KRE33 open reading frame (ORF) is expressed under the control of the strong constitutive TDH3 promoter and CYC1 terminator, resulting in overexpression of the protein.

h. Why does the final entry in table S2 read "plasmid vector control (Sckre33)". Is this an empty vector or a plasmid containing kre33?

We have corrected the final entry in Table S2 to read "Empty vector control" rather than "plasmid vector control (ScKre33)". This is indeed an empty vector that does not contain KRE33.

We have also improved the experimental description to go with these changes:

"**Yeast cell transformation.** Transformation of the parent strain (Δkre33 + pSH35-C, which harbors a genomic KRE33 deletion complemented by a catalytically deficient H545A allele on a URA3-marked plasmid) was performed with each HIS3-marked plasmid construct as previously described[71]. Briefly, a single colony of the parent strain was inoculated in 5 ml of YPD medium and incubated on a rotary shaker at 200 rpm at 30 °C for 16-20 h. To a flask containing 50 ml YPD medium, 2.5 x $10^8$ cells (a suspension containing 1 x $10^7$ cells $ml^{-1}$ will give an $OD_{600}$ of 1) were added to obtain a titer of 5 x $10^6$ cells $ml^{-1}$, and the flask was incubated at 30 °C, 200 rpm until the titer reached at least 2 x $10^7$ cells $ml^{-1}$. Cells were harvested by centrifuging for 5 min at 3000 g. The pellet was washed 2 times by resuspending in 25 ml sterile water and centrifuging for 5 min at 3000 g. The pellet was then resuspended in 1 ml of sterile water and transferred into a 1.5 ml Eppendorf tube. Water was removed by centrifuging for 30 sec at 13000 g, and the pellet was resuspended again in 1 ml of water. From this, 100 µl ($10^8$ cells) was pipetted into 1.5 ml Eppendorf tubes, one for each transformation, and centrifuged at 13000 g for 30 sec to remove the supernatant. The transformation mix (240 µl of PEG3350 (50% w/v), 36 µl of 1M LiAc, 50 µl single-stranded salmon sperm carrier DNA (2 mg ml^-1^), and 34 µL plasmid DNA (~500 ng) in sterile water) was added to each cell pellet and mixed by vortexing. A transformation was done without plasmid as a control. After incubation at 42°C for 40 min, cells were pelleted, resuspended in 200 µl YPD medium, and spread on SC-His plates to select for transformants carrying the plasmid of interest. Plates were incubated at 30 °C incubator for 3-4 days until colonies appeared. All transformations (WT KRE33, H545A, and other mutants) were performed using the same parent strain, allowing direct comparison between KRE33 variants. Successful transformation was confirmed by PCR amplification and Sanger sequencing of the mutated region of each plasmid, which was isolated using the Zymoprep yeast plasmid miniprep II kit (Zymo Research). For H545A transformants, genomic and plasmid-based H545A alleles were distinguished by PCR based on the presence of different codons for alanine 545 (GCA in the genomic copy versus GCT in the plasmid)."

Minor Issues:

1. It would be helpful to include a cartoon schematic of the acylation reaction as a supplemental figure to help readers understand how the synthesized probes mimic the NAT10 reaction.

We have now included a cartoon schematic of the acetylation reaction in **Supplemental Figure 2C**.

2. Lines 71-72 – The authors should consider rephrasing this sentence. NAT10 is essential because of its role in ribosome assembly and not because of its catalytic activity.

We have rephrased this sentence to more accurately reflect the current understanding of NAT10's essential functions:

The relevant text is:

" Previous mutational analysis in yeast indicate an essential role for NAT10's helicase domain, potentially due to its role in ribosome assembly. In contrast, the catalytic RNA acetyltransferase domain is required for optimal fitness at elevated temperatures, which is attributed to its role in tRNA modification [7,23,27-29]."

3. Line 95: In what way might NAT10 be a viable therapeutic target? This should be linked to NAT10's known activities (ribosome biogenesis, stress response, senescence, aging, and/or cancer) a little more explicitly. As is, this paragraph is gesturing a little vaguely at reasons why NAT10 might be important, in a way that actually makes the significance of the paper's results a little less accessible.

We thank the reviewer for suggesting this clarification. We have revised the Introduction section to more explicitly link structural characterization of eukaryotic NAT10 to therapeutic applications.

The relevant text is:

"While the role of NAT10 in disease and related data raise the possibility of targeting NAT10 for therapy, defining the druggable role of NAT10-catalyzed acetylation remains challenging. Eukaryotic NAT10 enzymes are large (~120 kDa) multidomain proteins. In addition to the Gcn5-related N-acetyltransferase (GNAT) domain directly responsible for $ac^4C$ formation, NAT10 also possesses an N-terminal ATP-dependent helicase domain, a C-terminal RNA binding domain, and several other regions of notable sequence homology. These regions interact with ribosome biogenesis factors and may contribute to the non-catalytic role of NAT10, the proper assembly and maturation of the 90S pre-ribosome [24-26]. Furthermore, mutation of NAT10's helicase and acetyltransferase domains cause distinct growth defects in *S. cerevisiae* [7,23,27-29]. Of note, many studies of NAT10 in human cells have exclusively used genetic techniques that target the entire gene, leaving the specific contribution of NAT10's acetyltransferase activity to phenotypes unknown. A putative small molecule inhibitor of NAT10 has been reported but is unsuitable for use as a chemical probe due to its pleiotropic off-target effects [33]. In addition, the substrate-spectrum of NAT10 is unclear. While NAT10's ability to catalyze the deposition of $ac^4C$ in ribosomal RNA (rRNA) and transfer RNA (tRNA) are well-validated, other protein and RNA substrates remain topics of inquiry. Altogether, these data highlight a need for molecular-scale characterization data capable of providing domain and substrate-specific insights into NAT10 functions."

4. Line 166: For “bisubstrate probes” could you more explicitly describe which part of the probe goes into which active site/ interacts with which domain?

We now include a description in the Results section of the rational for the probes used for these studies:

The relevant test is:

“Bisubstrate probes, designed to simultaneously occupy cofactor and substrate sites have proven valuable tools for studying acetyltransferase enzyme function.[37-39] To test whether this strategy could be potentially extended to RNA acetyltransferases, we designed and synthesized two compounds as possible mimics to the transition state, cytidine-acetone-CoA and cytidine-amide-CoA (**Figure 1D**),”

5. Line 174/ Figure 1C: Did you test thermal stability with acetone linker? Why was this excluded?

We unfortunately depleted our supply of the probe containing the acetone linker so could not test the thermal stability of the probe. We do not believe that omission of this data significantly detracts from the main findings of this study.

6. Lines 185 – 186, Supplemental Figure 3: Please add an image of the map after refinement steps but before C2 symmetry was applied, or note at exactly what step C2 symmetry was applied. Did the authors try masking half of the dimer and using symmetry expansion, or further processing what became their final map with 3D classification or 3D variability – have you ruled out the possibility that the two halves of the dimer are not perfectly symmetrical? Is it required that both ATP/ ADP and acetyl-CoA be bound in both dimers?

We thank the reviewer for raising this important point. We have added to new **Supplemental Figure 5** an image of the reconstruction obtained after the homogeneous refinement steps but prior to the application of C2 symmetry. To clarify our processing strategy: throughout the entire refinement workflow, the particle images were kept intact, without any special treatments such as masking of half-particles or symmetry expansion. C2 symmetry was imposed only at the final non-uniform refinement step. This decision was based on a detailed inspection of the homogeneous refinement map in C1, which revealed no significant differences between the two halves of the dimer in the densities for ATP/ADP and the bisubstrate probes. On this basis, we applied C2 symmetry in the final step to obtain an improved overall map. Furthermore, the C1 reconstruction indicates that both ATP/ADP and acetyl-CoA are bound simultaneously in both monomers. This point is made in the methods section.

7. Line 210/ Figure 2D: Please label the peak that corresponds to the dimer as “dimer.” Is the larger peak representative of a tetramer?

This has been labeled in the revised figure panel.

8. Line 338: In this line, please name the specific basic patch 2 mutants you are referring to. It is not straightforward to determine which mutants you mean here just by looking at Supplementary Figure 10.

The specific mutants are now named in the revised text.

9. Figure 4 and Supplemental Figure 10: Please label residues according to patch number. Color coding in Supplemental Figure 10 to be consistent with Figure 4 would be nice but labels alone would be sufficient (guaranteed to be clear even for those who are color blind).

For clarification, patch numbers have been added to **Figure 4F** and color-coded in **Supplemental Figures 10**.

10. Figure 4C: The green selection outline in 4C that highlights residues H548 and Y549 makes these atoms look green instead of yellow. Could you change the selection outline to either black or bolded yellow?

Residues H548 and Y549 are now rendered in yellow stick to more clearly highlight that they reside in the yellow GNAT domain.

11. Supplemental Fig 2 – it would be very helpful to add the domain labels/boundaries onto the sequence alignment as well as indicate the catalytic residues and putative RNA binding patches.

We have now added the domain label boundaries and indicate the catalytic residues/putative RNA binding residues to the alignment.

12. Lines 573 – 576: At what point in this elution protocol does your product elute?

This is now indicated in the methods section.

13. Line 631 – Were these RNAs folded or just resuspended in buffer?

It is now indicated in the methods section that the RNA probes were all reannealed before utilized for EMSA.

Reviewer #3 (Remarks to the Author):

I co-reviewed this manuscript with one of the reviewers who provided the listed reports. This is part of the Nature Communications initiative to facilitate training in peer review and to provide appropriate recognition for Early Career Researchers who co-review manuscripts.

Reviewer #4 (Remarks to the Author):

In this study, Zhou and colleagues aim to structurally and functionally analyse the eukaryotic RNA N4-cytidine acetyltransferase, NAT10. NAT10 catalyses cytidine acetylation of different RNA substrates, including 18S rRNA and tRNAs. The role of NAT10 during ribosome biogenesis has recently been complemented by cryo-EM structures of 90S pre-ribosomes in complex with NAT10. These structures and previous structures of the bacterial homologue TmcA revealed the overall architecture and domain organization of dimeric NAT10. The specific functional role of ac4C in eukaryotic tRNAs has remained elusive and our understanding of how NAT10 targets specific tRNAs and positions in different RNA targets is

very limited. The pathway seems to play a crucial role in biological/cellular functions (e.g. translation, senescence) and clinical data links mutations in the components to severe human pathologies. Hence, the study aims to answer interesting biological questions of a highly relevant and timely topic.

The authors purify eukaryotic NAT10 from the thermophilic fungus Chaetomium thermophilum and determine its single particle cryo-EM structure bound to a cytidine-CoA probe with and without ATP. They analyse the obtained structures and complement their work with functional analyses of structure-guided mutants. The structural work is carried out a state-of-the-art level and generally I don't see any major technical issues with any of the experimental approaches. However, I have major reservations about the relevance and impact of the obtained structural snapshots.

I appreciate the efforts of the authors to identify/map the respective regions in NAT10 that mediate tRNA binding – especially in the absence of a tRNA- and /or Tan1-bound structure. I was wondering whether the authors have tried to obtain such structures (with full tRNA or ASL) and whether they have any complementary data (SAXS, cross-linking MS, …) that would provide additional restraints and allow to generate a model of the complex during the catalytic step of the modification reaction. At the moment, it cannot be excluded that the complex undergoes major structural rearrangements during tRNA and/or Tan1 binding, whether the complex binds 1 or 2 tRNAs molecules at the same time and whether the mutated residues are anywhere close to the substrate. The lack of this kind of data represents a significant gap that clearly limits the impact of the study and might be more confusing than revealing – in particular, as the observed dimeric conformation has in principle been observed already in the pre-ribosome structures. I also think, that the results of the mutational analyses are rather confusing and misleading – I assume, that the role of the mutated residues can only be clarified/interpretated after obtaining a tRNA- and/or Tan1-bound structure of NAT10. At the moment this does not represent an informed structure guided approach.

Therefore, I am afraid that I am not able to support publication of the manuscript in its current form at Nature Communications. I encourage the authors to consider some of my comments for their future work and/or before finding a different target journal for the work.

As described in response to the summary of reviewer 2 above, we believe that capturing and NAT10 complex with tRNA and/or Tan1/Thumpd1 is of significant interest to us but goes beyond the scope of this current study. While previous structural and biochemical studies have focused on the structure/function of Nat10 in the context of pre-ribosome biogenesis, this study focuses instead on its acetyltransferase activity, and we have now added additional biochemical data that specifically addresses tRNA acetylation and the functional role of Tan1/Thumpd1. We hope that together, this persuades the reviewer of the significant new information provided by this study.

The major findings include **(1) The first structure of NAT10 in its catalytically competent state.** Previous structural studies captured NAT10 bound to pre-ribosomes in a non-catalytic context related to RNA assembly functions. In contrast, our study reveals NAT10 in its active, engaged conformation, with bisubstrate probes occupying both cytidine and CoA binding sites. **(2) Structure-guided identification and validation of catalytic residues.** We use our structural knowledge to guide functional studies directly demonstrating that NAT10 acetyltransferase activity is required for thermoadaptation and cellular senescence. **(3) Adapter-dependent tRNA substrate discrimination (<u>data new to this revision</u>).**

Biochemical reconstitution demonstrates that THUMPD1 specifically enhances NAT10 activity towards cognate ($tRNA_{Ser/Leu}$) but not non-cognate ($tRNA_{Ala}$) substrates. **(4) The specificity of RNA acetylation reflects differential catalytic efficiency rather than binding affinity** (**data new to this revision**). We demonstrate that NAT10 binds cognate and non-cognate tRNAs with similar affinity, but selectively acetylates only cognate substrates, a mechanism of RNA modification enzyme specificity that may apply broadly across this enzyme family. We have attempted to better highlight these novel findings in the revised manuscript.

Major

1. First, a more quantitative assay (e.g. SPR, MST, FP) to measure the RNA binding affinity is need to more quantitatively compare the various point mutants (Suppl. Fig. 9). For instance, the authors mention "Interestingly, none of the mutations significantly compromised RNA binding capability (Supplemental Figure 9)" – at the moment, this could result from the identification of the wrong residues or the lack of sensitivity of the assay – or both. The authors should also include a non-substrate tRNA and another folded RNA as negative controls (Fig. 1B).

This point is well taken. Based on this comment, we repeated the RNA binding studies comparing tRNA binding using gel shift and incorporating a control non-cognate tRNA substrate ($tRNA^{Ala}$) for comparison with the cognate $tRNA^{Ser}$ and $tRNA^{Leu}$ substrates. The *Ct*NAT10 protein demonstrated comparable binding to both cognate and non-cognate substrates. Based on these results we conclude that the binding of NAT10 to tRNA (at least in the absence of Tan1/THUMPD1) is non-specific.

We then carried out tRNA-binding experiments in the presence of THUMPD1 with both NAT10 WT and 2 selected basic-patch mutants. Since gel shift experiments revealed multiple shifted bands, we decided to use gel shift rather than FP in order to deconvolute the different complex species (likely not resolved in the FP experiment). These experiments revealed that NAT10 binding to tRNA is comparable between cognate and non-cognate tRNA (while acetylation only occurs with the cognate $tRNA^{Ser}$ and $tRNA^{Leu}$ substrates) indicating that discrimination of RNA acetylation occurs at a step after tRNA binding. These new results are now described in Results section and shown in new **Supplemental Figure 4**.

2. In general, the results from the used functional assays are highly inconsistent and present a rather confuse picture. The authors do mention this themselves - e.g. "Interestingly, one patch 3 mutant (hR421A/CtR422A) and patch 2 mutants (hY835A/CtY851A and hH836A/CtH852A) in both the human cell-based senescence assays and the yeast cell-based fitness assay scored as WT or near WT, but were either inactive or impaired in CtNAT10 biochemical assays, suggesting some differences between the in vitro and cellular systems." It seems very strange that mutations that were chosen due to there possible involvement in tRNA binding, do not "significantly compromised RNA (Supplemental Figure 9)" at all – but somehow they all affect the activity. Furthermore, in yeast mutants in "basic patches 1 and 3 failed to rescue the temperature sensitivity, while basic patch 2 mutants did show some rescue (Figure 5B and Supplemental Figure 10)" – in human cells, only one "patch 3 mutant and the active site mutants exhibited a markedly decreased p16 expression, while the rest of the mutants recapitulated the effect of WT-NAT10" – hence, the inconsistencies go far beyond what has been mentioned. I simply don't see that the authors managed to bring the structural and functional data together and this part is very confusing to the reader.

The reviewer brings up a very good point about the lack of agreement between our functional in vitro and cellular data. For our initial submission, the functional in vitro data was carried out usng an 18S rRNA substrate, believed to be dependent on the presence of a snoRNA, which was not included in our in vitro assay. rRNA acetylation could also play an essential role in ribosome biogenesis. For this reason, we decided to replace our functional in vitro data with assaying tRNA acetylation (an activity known to be non-essential) in the presence of the THUMPD1 cofactor protein. These studies are now shown in revised **Figure 4E**, and reveals patch 1 and 3 mutants inhibit tRNA acetylation, while patch 2 mutants have wild-type tRNA acetylation activity. These results are now consistent with the findings of the cellular yeast thermoadaptation (**Figure 5C**) and human cellular senescence data (**Figure 6E**). This is consistent with these cellular phenotypes being associated with defects in tRNA acetylation.

3. The authors need to show the comparison between the free and pre-ribosome bound Ct/ScNAT10 complexes. At the moment they state that their analyses shows the homodimer forms already in solution, but it would be enlightening to see if any (even small) structural changes can be observed between the free and pre-ribosome bound NAT10 structures. I am not sure whether the authors checked the deposited maps of the pre-ribosome structures for the presence of ATP, acetyl-CoA or other intermediates in the respective active sites – this needs to be analysed to allow for a fair comparison.

In **Supplemental Figure 8**, we now show a superposition of our CtNAT10 structure with the pre-ribosome-bound Nat10 structures, revealing no significant structural changes. We have also downloaded the cryo-EM maps of the pre-ribosome/Nat10 structures and do not see evidence for bound ATP, CoA or other cofactors. This is now described in the results section.

4. Towards a structural analyses of the tRNA-bound state, the authors should check the thermostability in the presence of ATP/ATP-analogues/acetyl-CoA and RNA substrate. They should also quantitatively measure the effect of ATP/ATP-analogues/acetyl-CoA on RNA binding.

To probe if tRNA and THUMPD1 binding to *Ct*NAT10 have significant effects on *Ct*NAT10 structure, we evaluated the thermal stability of *Ct*NAT10 as a function of tRNA and THUMPD1 binding (+/- cofactor binding) using a nano differential scanning fluorimetry (nanoDSF) assay This revealed no significant thermal stability change of *Ct*NAT10 when bound to tRNA and THUMPD1(in the presence or absence of cofactors), suggesting that binding of the THUMPD1 cofactor and tRNA substrate does not significantly alter the structure of *Ct*NAT10, thus suggesting that they likely bind to the enzyme surface. This new data is now shown in the Supplemental **Figure 4B** and described in the Results section.

5. Please provide the non-symmetrized reconstructions in the manuscript – this is particularly important as the lower signal of the ligands in the biding sites might result from lower overall occupancy or the presence of a ligand molecule in only one of the active sites at a time, which would have far-reaching functional implications – leading to the assumption of an asymmetric complex that can only modify one tRNA at a time.

This important point is addressed under Reviewer 2, Minor points, #6. Specifically, we have added to Supplemental **Figure 5** an image of the reconstruction obtained after the homogeneous refinement steps but prior to the application of C2 symmetry. To clarify our processing strategy: throughout the entire refinement workflow, the particle images were kept intact, without any special treatments such as masking of half-particles or symmetry

expansion. C2 symmetry was imposed only in the final non-uniform refinement step. This decision was based on a detailed inspection of the homogeneous refinement map in C1, which revealed no significant differences between the two halves of the dimer in the densities for ATP/ADP and the bisubstrate probes. On this basis, we applied C2 symmetry in the final step to obtain an improved overall map. Furthermore, the C1 reconstruction indicates that both ATP/ADP and acetyl-CoA are bound simultaneously in both monomers. This process is described in the Methods section.

6. I am not convinced that the obtained structures of the cytidine-CoA probes do represent catalytically relevant states at all and rather reflect the pre-reaction acetyl-CoA bound that has previously been reported already. The authors do not show whether there are any differences in the coordination of the probes and the previously determined structure of TmcA bound to acetyl-CoA and ADP – in my opinion the scientific insights are very limited. The author do mention that “Inspection of the active site proximal to the cytidine nucleotide reveals a somewhat open pocket with no residues in proximity to make direct contacts suggesting that a structural rearrangement may be required for N4-cytidine acetylation”, but I think the current text of the manuscript overstates the novelty of the resolved structures. I believe the authors designed their study to observe specific reaction intermediates, but ended up with the already known states. The analyses of the fungal CtNAT10 bound to acetyl-CoA and ADP might be more relevant than the presented structures – however, also less exciting of course.

We agree with the reviewer that the structures that we captured likely represent the pre-reaction state and have modified the language in the revised Discussion section to reflect this.

The relevant text is:

“While our cytidine-CoA probes were designed to capture a transition state intermediate, the structure as observed likely represents a pre-reaction state, since the cytidine portion is partially disordered and the essential catalytic residues (*Ct*H548 and *Ct*Y549) are relatively far from the exocyclic N4 amine. This suggests that significant conformational rearrangements are required to bring the catalytic machinery into proper geometry for N4-cytidine acetylation.”

Minor

• It was not mentioned whether there was any specific reason of determining the different structures from bacterial or insect cell source. It might just have happened that way, but it would be nice to comment on why the authors went for both expression strategies and if there was any specific rationale.

In the revised manuscript, we now more clearly describe that active CtNAT10 protein could be expressed in both E. coli and insect cell systems, was used interchangeably for structural studies. Since the insect cell protein showed slightly higher activity. it was used for all activity assays.

• What happened in suppl. Figure 9a at concentration 0.81uM wt and R422A?

We have removed the in vitro rRNA functional data and replaced it with in vitro tRNA functional data as described in response to point 2 above.

• Which steps of the processing (Suppl. Fig. 3) were performed with binned and unbinned

particles. I would refer to “picks” rather than particles before the initial 2D classification step – I doubt the author excluded 5.500.000 real protein “particles”.

The raw super-resolution data were subjected to 2x binning during motion correction. After particle extraction, the particles were further binned by an additional factor of 2, and these binned particles were used for all subsequent processing steps up to and including heterogeneous refinement, from which a reasonable volume class was obtained. Following this, the particles were re-extracted without binning and unbinned particle stack were then used for all high-resolution refinement steps, including homogeneous refinement and final non-uniform refinement (Nu-refinement), to obtain the final high-resolution maps. This is now elaborated in the Methods section. This is now described in the Methods section.

We also accepted the suggestion of the reviewer and changed the “particles” to “picks” to ensure the rigor of the description.

• It is clear from the alignment that the residues that form the dimerization interface are not conserved/present in bacterial TmcA. It would be nice for the reader to show a structural representation of this analysis (similar to Fig. 2C).

A structural alignment of CtNat10 and TmcA, with a closeup for the putative RNA-binding domain) is now shown more clearly in revised **Figure 2F**.

• I don’t understand the representation and description of Figure 2F – this needs to be improved. Could the authors speculate whether the difference in the position of the RNA binding domain between NAT10 and TmcA could be caused by crystal contacts in the bacterial structure or the homodimerization – I simply got confused by the figure and the interpretation – “Interestingly, the RNA binding domain of the symmetry related subunit superimposes much more closely to the position of the RNA binding domain of EcTmcA (Figure 2F), thus further supporting the biological importance of the dimeric state of NAT10.” The position of the domain is similar in the pre-ribosome bound structure, right – please add a comment.

We agree that rendering in **Figure 2F** of the previous submission was confusing, and we have tried to simplify it in the revised figure panel. We believe that if TmcA was a functional dimer, that it would be unlikely for crystal packing forces to disrupt such a dimer, arguing for its monomeric functional state. As mentioned above, the pre-ribosome NAT10 dimer structure is essentially identical.

• “In the CtNAT10/cytidine-amide-CoA/ADP structure, the ADP and Mg2+ are wedged in between the helicase and DUF domains, and most of the protein interactions are mediated by the α-helix bundle (α9-α12) from the helicase domain (Supplemental Figure 7).” – I think the reference to this figure is suboptimal and the information could be included in Figure 4B (or 4A).

The reviewer brings up a good point and we have revised **Figure 3C** to more clearly lustrate this point.

• Figure 3A – ATP hydrolysis and magnesium coordination. In my opinion, the map interpretation is highly speculative and should be complemented by ATP hydrolysis measurements – or the section should at least be rephrased, as it is rather based on previous observations by others than the actual data of this study.

We appreciate the reviewer's comment and have revised the main text accordingly to clarify that the interpretation is based on previous structural observations rather than direct biochemical evidence from this study. The reason we modeled ADP + $Mg^{2+}$ instead of ATP is: (1) this assignment is consistent with prior observations in the TmcA crystal structure; and (2) when attempting to fit ATP into the density, we were unable to obtain a chemically reasonable geometry during refinement, whereas ADP + $Mg^{2+}$ yielded a satisfactory fit.

**RESPONSE TO EDITOR AND REVIEWERS**

**Editorial request**

You will see that, while the reviewers find that your revisions improved the manuscript, some important points remain to be addressed. Editorially, we would not require additional work on the NAT10–THUMPD1 complex, but please address concerns regarding the presence of the NAT10–THUMPD1 complex (Reviewer #4 major issue 4). Please revise your manuscript, addressing all the remaining issues raised by the reviewers.

Thank you for the opportunity to submit a revised manuscript. In the revised manuscript, we have addressed the criticisms of the reviewers as outlined below:

**REVIEWER COMMENTS**

**Reviewer #2 (Remarks to the Author):**

Please note to prepare this review we participated in the Nature Comms new Early Career Researcher review program.

The authors have adequately addressed the majority of our previous concerns. We appreciate the additional assays including the demonstration that THUMPD1 enhances activity towards cognate tRNAs and the new data that supports specificity of RNA acetylation reflects differential catalytic efficiency rather than differences in tRNA binding affinity. We also appreciate the improved description of the yeast-based assays.

Minor Issues:
1. We find Figure 1A hard to follow and do not agree with the authors designation of positive and negative controls. The positive control is not a control but rather the reaction being studied with all the components present. We suggest coming up with a better format for this figure – perhaps separating the end point and time courses rather than showing them side-by-side. Using the format to designate what components are present from Supplemental Fig. 4B would also be helpful.

We have reorganized **Figure 1A** to separate out the endpoint (left) and time course (right), and used the format to designate what components are present, to be consistent with **Supplemental Figure 4B** (now changed to **Supplemental Figure 4C**), as suggested by the reviewer.

2. In Figures 2-4 it is not always clear which of the three determined structures are shown in each panel. It would be helpful to readers to add the PDB codes to the figure legends to make this more clear. For example, in Fig. 3A is the ADP density from the Sf9 or EC structure? Are 3A and 3B from the same structure? If so it would be better to use the same color for ADP and Mg in both.

This has now been clarified in the corresponding figure legends. Since **Figures 3A** and **3B** come from the same Sf9 structure, we have also unified the color scheme of the two figures as suggested by the reviewer.

**Reviewer #4 (Remarks to the Author):**

I have carefully reviewed the revised manuscript, now entitled "Structure of the NAT10 Acetyltransferase and Mechanism of RNA Acetylation".

The authors have performed additional experiments and carefully rephrased the manuscript. In addition, the focus shifted away from the activity of NAT10 on rRNA towards the role of NAT10 in tRNA acetylation.

I think the current text of the manuscript improved on down-toning previous overstatements about the novelty of the resolved structures. I recognize that the authors designed their study to observe specific reaction intermediates, but ended up with the already known states. I repeat myself from the first round of reviews – "The analyses of the fungal CtNAT10 bound to acetyl-CoA and ADP might be more relevant than the presented structures – however, also less exciting of course." In my opinion, some interesting results were included, but they not necessarily validated previous claims/assumptions – rather the authors removed the previously acquired dataset and replaced it with another apparently more consistent one. For all of the above reasons, the number of presented results increased, but the consistency between the results did not necessarily follow this trend. In my opinion, the actual scientific advances described in the manuscript still does not justify publication in Nature Communications.

We appreciate the reviewer's comments but would like to stress that we have made several novel and significant findings in the revised manuscript including (1) The first structure of NAT10 in the absence of the ribosome and in its catalytically competent state; (2) Structure-guided identification and validation of catalytic residues; (3) Adapter-dependent tRNA substrate discrimination; and (4) The specificity of tRNA acetylation reflects differential catalytic efficiency rather than binding affinity. The latter two conclusions are new to the revised manuscript.

We chose to replace the biochemical data on rRNA acetylation with tRNA because it allowed us to specifically address the role of THUMPD1 cofactor protein on RNA acetylation, which we believe added novelty to the study as described above.

Major issues

1. The authors state that "While our cytidine-CoA probes were designed to capture a transition state intermediate, the structure as observed likely represents a pre-reaction state, since the cytidine portion is partially disordered and the essential catalytic residues (CtH548 and CtY549) are relatively far from the exocyclic N4 amine. This suggests that significant conformational rearrangements are required to bring the catalytic machinery into proper geometry for N4-cytidine acetylation." Would the nanoDSF data not indicate the opposite, showing that neither tRNA nor THUMPD1 binding to CtNAT10 have significant effects on structure. Either one of the techniques is not appropriate or there something else that is incorrectly interpreted/analyzed.

We apologize for the lack of clarity in our description of what we believe the structure and nanoDSF data reveal about the conformational changes that likely occur to facilitate RNA acetylation by NAT10. Since the structure of NAT10 bound to the cytidine-CoA probes shows that the essential catalytic residues of NAT10 are too far from the exocyclic N4 amine, we conclude that the structure is not a catalytically competent state, and therefore likely requires some structural rearrangement for catalysis. The nanoDSF data also demonstrate that tRNA

and THUMPD1 binding are likely insufficient to induce the required conformational changes. We propose that tRNA, THUMPD1, acetyl-CoA binding, and ATP binding and hydrolysis are required for the structural changes required for catalysis. This point is now described in the discussion section.

2. It is interesting to see that the newly incorporated data on the tRNAs modification activity is much clearer than for the previously used rRNA. Considering that the set of mutants reacts so differently to the two types of RNAs, I was wondering what is so different with these substrates – the logic of basic patches binding RNAs should be valid for tRNAs as well as rRNAs. One question – what is the scientific reason for excluding the previous analyses/data for the rRNA?

For the revised biochemical studies, we were concerned, as were the reviewers, that studying NAT10 binding to RNA in the absence of the required cofactors, THUMPD1 for tRNA binding/acetylation and snoRNA for rRNA binding/acetylation, might represent non-specific activity. We therefore decided to study a system that would allow us to prepare a biochemically well-folded and active cofactor. We therefore redid our biochemical in vitro studies with tRNA and THUMPD1 because we could confidently prepare a well-folded THUMPD1 cofactor protein that was active for tRNA binding (**Supplemental Figure 4**). We were concerned that the preparation of a properly folded and active snoRNA cofactor for NAT10-mediated rRNA acetylation could not be assured without the added presence of snoRNA chaperone proteins.

3. The authors state correctly "This is consistent with these cellular phenotypes being associated with defects in tRNA acetylation." Now I start to wonder, why NAT10 would be associated with ribosome biogenesis at all, if the phenotypes correlate to well only with the results from the tRNAs. Do the authors think that was a technical problem with the used rRNA and if they knew that probably a snoRNA is needed, why did they not try include it (or why did include the previous data into the first version of the manuscript)?

Although our cellular data is consistent with the role of the RNA acetylation activity of NAT10 in the observed cellular phenotypes, we do not distinguish whether this is due to tRNA acetylation, rRNA acetylation or both. This point has been clarified in the revised text.

The role of NAT10 in ribosome biogenesis is beyond the scope of this study. Nonetheless, given that the ribosome biogenesis function of NAT10 does not depend on its catalytic activity, this suggests that its ribosome biogenesis activity is not sufficient to maintain the fungal thermoadaptation and mammalian cellular senescence processes studied here. This point is described in the revised discussion section.

4. Foremost, the new data with THUMPD1 (Fig. 1) clearly ask for some interaction assays and at least a structural prediction of the complex. In addition, the use of different concentrations of NAT10 and THUMPD1 in the presented EMSAs (Supplemental Figure 4) are slightly confusing. Foremost, I was wondering how the authors interpret the results of the "complex" at high concentrations (e.g. 1750, 3500 and 7000 nM) – it looks like the complex gets smaller again. This leads me to the other question – do they form a complex at all or do they just bind the same tRNA molecule. The authors seem to assume this by stating – "This result underscores the rate acceleration conferred by THUMPD1 as well as the substrate specificity of the NAT10–THUMPD1 complex towards tRNA." – however I am missing any experimental evidence for the presence of the "complex". Here several question arise from the interesting data (e.g. do the basic patch mutants still interact with THUMPD1) and I am inclined to say that

additional work on this complex would make a much more interesting story – I am sure the authors are also aware of this.

We agree with the review that the proteins and their stoichiometries that are present in tRNA shifted bands shown in **Supplemental Figure 4B** cannot be reliably determined, which is why we have not attempted to identify the components of the shifted bands in this figure panel. We only use this figure panel to demonstrate that the pattern of shifted bands does not depend on the presence of cognate tRNA, as the patterns look similar for the cognate ($tRNA^{Leu}$ and $tRNA^{Ser}$) and non-cognate ($tRNA^{Ala}$) tRNA (**Panel A**); and independent of catalytic activity, illustrating two mutants that show background levels of catalytic activity (NAT10-K427A and NAT10-637A). We chose a stoichiometry of THUMPD1 to tRNA of 3:1 to ensure that the level of THUMPD1 was not limiting. Together, these results suggest that the orientation of the tRNA binding site on NAT10, rather than affinity driven by NAT10 and THUMPD1, is the rate-determining step for catalysis. These points are now clarified in the revised text.

The reviewer raises a very good point, demonstrating that tRNA incubated with NAT10 and THUMPD1 indeed forms a complex that contains both proteins. To demonstrate this, we conducted an additional experiment in which we performed mass photometry of the complex, with and without THUMPD1 or NAT10 at concentrations comparable to those shown at the highest concentrations titrated in **Supplemental Figure 4A**. Since Mass photometry experiments can only be carried out at low nanomolar concentrations, prior to diluting for mass photometry, we crosslinked the samples with glutaraldehyde to prevent dissociation of the complexes formed at the higher concentrations. These experiments are now shown in new **Supplemental Figure 4B** and demonstrate that a complex of molecular weight of 362 KDa is formed when NAT10, tRNA and THUMPD1 are present, but not when either NAT10 or THUMPD1 are omitted. This molecular weight is most consistent with a calculated molecular weight of a NAT10/THUMPD1/tRNA complex with a stoichiometry of 2/2/2. Notably, this complex is not formed in the uncrosslinked sample, suggesting that this complex does not form in the low nanomolar concentration range. These new results are now discussed in the Results section entitled Electropositive patches on the NAT10 surface participate in RNA acetylation.

5. As the results from the mutants are now consistent between the in vitro modification activity and the cellular assays, it would be crucial to measure the actual tRNA modification levels in the yeast and human cells to directly correlate the phenotypes with decreased/unchanged tRNA modification levels.

We wish to clarify that the biochemical in vitro studies focused on tRNA acetylation, rather than rRNA acetylation, because we were confident that we could prepare well-folded THUMPD1 cofactor protein that was active for tRNA-binding (**Supplemental Figure 4**). As described above, reparation of well- folded and active snoRNA cofactor, without the presence of snoRNA chaperones, for NAT10-mediated rRNA acetylation was not assured. To directly address the reviewer's request, we have now measured ac4C levels in yeast at both 18S rRNA helix 45 (Sc-h45) and $tRNA^{Ser}_{AGA}$ using quantitative nucleotide-resolution $ac^4C$ sequencing across strains expressing WT *Sc*Kre33, catalytic mutant ScH545A, patch 1 mutant ScR637A, patch 3 mutant ScR420A, patch 2 mutant ScY852A, and empty vector (**Figure 5C, Supplemental Figure 12**). The results are now fully concordant across all three levels of analysis. At both the rRNA and tRNA substrates, only the WT and patch 2 mutant ScY852A (which retains in vitro

acetyltransferase activity) were able to restore ac4C to WT levels. By contrast, the inactive mutants ScH545A (active site), and ScR637A and ScR420A (patch 1 and 3) failed to rescue ac$^4$C at either substrate. This pattern mirrors both the in vitro biochemical activity data and the yeast growth rescue phenotype: mutants that rescue growth restore ac$^4$C, and mutants that fail to rescue growth do not. This three-way concordance between in vitro acetyltransferase activity, cellular RNA modification levels, and organismal fitness, directly addresses the reviewer's concern and demonstrates that NAT10's catalytic activity is the functional determinant of yeast thermoadaptation.

The revised text reads **"**To confirm that rescue of growth correlated with rescue of RNA acetylation, we applied nucleotide resolution ac$^4$C sequencing to total RNA extracted from yeast strains expressing WT *Sc*Kre33 or key mutants.[45] Assessing 18S rRNA helix 45 (*Sc*-h45), we observed efficient rescue of ac$^4$C1773 by the WT plasmid, while neither the catalytic mutant ScH545A nor the patch 1 mutant ScR637A had any effect. To directly test whether these phenotypes also correlated with tRNA acetylation - the substrate for which in vitro activity was characterized – we extended this analysis to $tRNA^{Ser}_{AGA}$ across WT, ScH545A, ScR637A, ScR420A, and ScY852A strains. Only WT and the patch 2 mutant ScY852A restored tRNA ac4C to levels comparable to WT, while the active site mutant ScH545A and the patch 1 and 3 mutants ScR637A and ScR420A did not (**Figure 5C and Supplemental Figure 12**). The concordant effects of active site and basic patch mutations on biochemical activity, cellular ac$^4$C, and organismal fitness establish that the acetyltransferase activity of NAT10 is the functional determinant of yeast thermoadaptation, and directly link the biochemically characterized residues to modification of both tRNA and rRNA substrates in a living cell.

Minor issues:

• "rRNA acetylation could also play an essential role in ribosome biogenesis. For this reason, we decided to replace our functional in vitro data with assaying tRNA acetylation (an activity known to be non-essential) in the presence of the THUMPD1 cofactor protein"…I don't follow the logic in linking these two statements?

As described in addressing point 2 above, we believe that the tRNA/THUMPD1 system offered a more robust in vitro system to study the acetylation activity of NAT10 in the presence of its cofactor protein.  The current literature suggests that while rRNA acetylation may occur during ribosome biogenesis, it is not strictly required for this process. The appropriate references are now cited.

• The number of picks/initial particles is inconsistent between Table 1 and Supplemental Figure 5.

We have now corrected this error by changing the value in Table 1.

• Page 2 – "acetylation is catalyzed by a single" something weird with the font size

This has been fixed.

• The following part of the introduction is highly speculative – "In addition, the substrate-spectrum of NAT10 is unclear. While NAT10's ability to catalyze the deposition of ac4C in ribosomal RNA (rRNA) and transfer RNA (tRNA) are well-validated, other protein and RNA

substrates remain topics of inquiry. Outside of the association of tRNA acetylation with neurodevelopmental defects, what specific RNA substrates mediate individual disease phenotypes is largely unknown 32. Altogether, these data highlight a need for molecular-scale characterization data capable of providing domain and substrate specific insights into NAT10 functions." and I don't understand how the current study helps to resolve this issues – by deleting the rRNA data it becomes even more limited -

In the revised introduction, we now modify this section to reference studies that demonstrate that the spectrum of NAT10 substrates may include mRNA and proteins, and state "Altogether, these data highlight a need for data capable of relating structure and biochemical activity on NAT10 acetylation to specific cellular phenotypes," which now more specifically highlights what is addressed in this study.

Other issues
I have also read the responses to the other reviewers and a couple of specific points caught my attention.

• "due to current restrictions at the NIH we are unable explore the alternative strategy suggested by the reviewers" – why would this ever be a reasonable explanation to not perform an experiment – worst case, you have to as somebody that does not have those restrictions to perform the experiment for you.

We appreciate the reviewer's comment and acknowledge that we could have addressed this suggestion more directly. Regarding the use of a Tet-off promoter to repress endogenous *Sc*Kre33 to assess the effects of Kre33 mutants on temperature-sensitive growth: we agree this is a useful approach in principle. However, even if performed, this experiment would not change the conclusions of the paper for the following reasons. First, the strain background we employ (Δkre33 + pSH35-ScH545A) is a well-validated system that has been used previously to study Kre33 function (Sharma et al., NAR, **2015**). Our validation data (**Figure 5B**) confirms that WT Kre33 rescues the heat-sensitive phenotype, while the catalytic dead H545A variant does not, establishing the expected controls. Second, as described above, we have now extended this analysis with quantitative $ac^4C$ sequencing, demonstrating that rescue of growth correlates precisely and completely with rescue of both rRNA and tRNA acetylation across all mutants tested (**Supplemental Figure 12**). The concordance between in vitro activity, cellular modification levels, and organismal phenotype makes the mechanistic interpretation robust regardless of the specific strain background used to assess growth. We have reviewed the manuscript to ensure the rationale for the chosen experimental system is clearly explained and why the conclusions are well-supported by the totality of the data.

• "We unfortunately depleted our supply of the probe containing the acetone linker so could not test the thermal stability of the probe." This sounds like the authors are not able to challenge, reproduce or validate any data that is related to the probe. In this case, the data should probably be taken out of the manuscript.

We wish to clarify that we could resynthesize the probe containing the acetone linker. However, given that our structures show that the probes containing the amide and acetone linkers are essentially superimposable, our expectation is that the thermal melt curve with probe containing the acetone linker will be similar to that containing the amide linker.

• "For clarification, patch numbers have been added to Figure 4F and color-coded in

Supplemental Figures 10.” – Figure 4F does not exist and am not sure which panel in Supplemental Figure 10 is the relevant one?

We apologize for the error caused by reorganizing the figure panels. The patch numbers have been added to **Figure 4D** and color-coded in Supplemental **Figure 10D**.

**RESPONSE TO REVIEWER AND EDITOR**

**REVIEWER COMMENTS**

**Reviewer #2**
The authors have addressed my previous concerns and I am supportive of publication.

We thank the reviewer for their helpful comments throughout the review process.

**Reviewer #4**
I have carefully reviewed the rerevised manuscript entitled “Structure of the NAT10 Acetyltransferase and Mechanism of RNA Acetylation”. In the rerevised version, the authors have put additional efforts in addressing and answering all issues raised. I appreciate the newly provided data and the explanations and I support publication of the revised manuscript in Nature Communications.

As the focus shifted away from the activity of NAT10 on rRNA and towards the role of NAT10 in tRNA acetylation, I would suggest to change the title to “Structure of the NAT10 Acetyltransferase and Mechanism of tRNA Acetylation”.

Finally, I would like to congratulate the authors for this comprehensive piece of work and I am looking forward to seeing the follow-up results on the newly defined NAT10–THUMPD1 complex – based on the finding described in this manuscript.

We thank the reviewer for their helpful comments throughout the review process.

We have changed the title as suggested by the reviewer.

**EDITORIAL COMMENTS**

We have revised the manuscript according to the Author Checklist as described in the checklist
